# Machine Learning and Deep Learning for Early Disease Detection Across Modalities: A Comprehensive Review of Methods, Applications, and Clinical Translation

**DOI:** 10.3390/diagnostics16193121

**Published:** 2026-09-25

**Authors:** Ahlem Aziz, Seydi Kacmaz, Azam Isam Abdulkareem Aladwani

**Affiliations:** 1 Electrical and Electronics Engineering Department, Karabük University, 78050 Karabük, Türkiye; 2 Department of Electrical and Electronics Engineering, Faculty of Engineering, Gaziantep University, 27310 Gaziantep, Türkiye; seydikacmaz@gantep.edu.tr; 3 Department of Drone Techniques Engineering, Technical College of Mosul, Northern Technical University, Mosul 41001, Iraq; azzam.esam@ntu.edu.iq

**Keywords:** early disease detection, machine learning, deep learning, medical imaging, biosignals, electronic health records, omics, wearable sensors, multimodal learning, external validation, clinical translation

## Abstract

Early disease detection is not a single classification problem. It includes population screening, presymptomatic risk estimation, identification of early pathological change, and detection of disease before conventional clinical recognition. Machine learning and deep learning systems approach these targets through medical images, physiological signals, longitudinal health records, molecular measurements, wearable sensors, and multimodal combinations. This survey organizes the evidence around the complete decision pathway linking the clinical target, data generation process, preprocessing, representation, model, validation design, reported performance, methodological limitation, and level of clinical translation. The analysis distinguishes early detection from ordinary diagnosis, prognosis, and progression prediction, as merging these tasks can make apparently strong results clinically misleading. Across modalities, deep learning is most useful when spatial, temporal, or cross-modal representations must be learned from sufficiently large and representative data. Traditional machine learning remains competitive when cohorts are modest, clinically meaningful variables are available, or transparent feature-level reasoning is required. The evidence also shows that performance is strongly influenced by patient-level separation, cohort construction, prevalence, label definition, missingness, external validation, calibration, and the handling of uncertain cases; therefore, high discrimination on an internal split cannot establish clinical utility. The survey further examines the gap between retrospective model development and deployment, emphasizing temporal and external validation, prospective workflow evaluation, decision thresholds, false negative consequences, demographic robustness, interpretability, and post-deployment monitoring. The resulting synthesis provides a modality-aware and clinically grounded framework for deciding not only which algorithms perform well but also what their results mean and what evidence remains necessary before they can support early clinical action.

## 1. Introduction

Machine learning (ML) and deep learning (DL) are now being applied to disease-related data generated before, during, and after clinical diagnosis; however, these applications do not all answer the same clinical question. A model that separates confirmed cases from healthy controls measures diagnostic discrimination under the sampled conditions. A screening model instead operates before definitive diagnosis, and must control the downstream burden created by false positives. A risk model estimates the probability of a future state over a defined horizon, whereas a prognostic model estimates outcomes after disease is already established. Treating these targets as interchangeable obscures the actual value and risk of the model.

The difference is visible even within a single disease domain. Deep-LIBRA estimates breast density from digital mammograms and evaluates the resulting representation in an independent breast cancer case–control dataset rather than claiming direct tumor detection [1]. Its development involved 15,661 images from 4437 women, followed by independent testing on 6368 mammograms from 414 cases and 1178 controls [1]. The resulting AUC of 0.612 is modest compared with many image classification reports, yet measures risk discrimination on a harder and clinically different task [1]. This example illustrates why a lower value obtained on an independent risk assessment target may carry more relevant evidence than near-perfect accuracy from a restricted case–control classification experiment.

The same distinction applies to longitudinal prediction. The IMBALMED framework evaluates both contemporaneous Alzheimer disease classification and four early-detection horizons extending from 12 to 48 months [2]. Its explicit separation of diagnostic and future-oriented targets permits the results to be interpreted according to the decision being supported [2]. By contrast, a study that labels established Alzheimer disease from structural magnetic resonance imaging cannot be reclassified as early detection merely because earlier treatment would be desirable.

Data modality further changes what can be inferred. Imaging captures spatial manifestations that may already be anatomically visible, whereas electroencephalography, electrocardiography, speech, and motion measurements encode physiological dynamics. Electronic health records (EHRs) supply longitudinal trajectories shaped by both disease and healthcare utilization. Molecular data may reveal biological change before a macroscopic lesion becomes visible, but high dimensionality and small cohorts create a severe risk of optimistic feature selection. Wearables increase temporal coverage, yet repeated measurements from the same participant make subject-level separation essential. while multimodal learning can combine complementary evidence, gains may reflect sample selection or information availability rather than fusion itself.

Recent studies demonstrate both the opportunity and the interpretive difficulty. A temporal diabetic retinopathy system combined 25,000 retinal images with up to 20 years of EHR history from 5000 patients [3]. The multimodal model reported an AUROC of 0.988, whereas the temporal EHR-only model reported 0.881 [3]. This comparison suggests that retinal appearance adds strong information; however, independent-institution validation was not reported for that evaluation, so the magnitude of transportable benefit remains unresolved [3]. A coronary artery disease model that integrates coronary computed tomography angiography, clinical variables, laboratory results, and electrocardiography obtained an internal AUC of 0.89 and an external AUC of 0.83 [4]. This decline under external validation is not a failure; rather, it is evidence that the internal estimate did not fully represent deployment variation.

Therefore, validation design is part of the result rather than a secondary reporting detail. An ECG-based CKD model was developed using 72,618 ECGs from 49,632 patients, internally evaluated on 16,955 non-overlapping patients, and externally evaluated on 10,476 patients from a community hospital [5]. Its AUC decreased from 0.885 internally to 0.861 externally, providing a direct estimate of transport beyond the development setting [5]. The model also identified elevated incident-CKD risk among patients without baseline CKD, which moves the contribution from static classification toward clinically relevant risk stratification [5]. Nevertheless, retrospective association with later outcomes does not by itself show that model-guided screening improves patient outcomes.

This survey evaluates ML and DL for early disease detection through the following chain:

Clinical target → modality → preprocessing and representation → model → validation → performance → limitation → clinical translation.

Because the included studies use different diseases, cohorts, endpoints, prevalence levels, and test designs, the survey does not rank algorithms by pooled accuracy; instead, it asks whether the learning method is appropriate for the available data, whether the evaluation isolates independent patients and sites, whether the reported metric matches the decision, and whether the evidence supports the claimed level of clinical use. Its added value is a common evidence framework that links six data modalities, major ML and DL method families, disease-oriented applications, validation maturity, and clinical translation without treating different benchmark values as a single leaderboard. The synthesis further distinguishes classification from segmentation, conventional from generative augmentation, and architectural novelty from evidence of transport or clinical utility.

**Figure 1:**
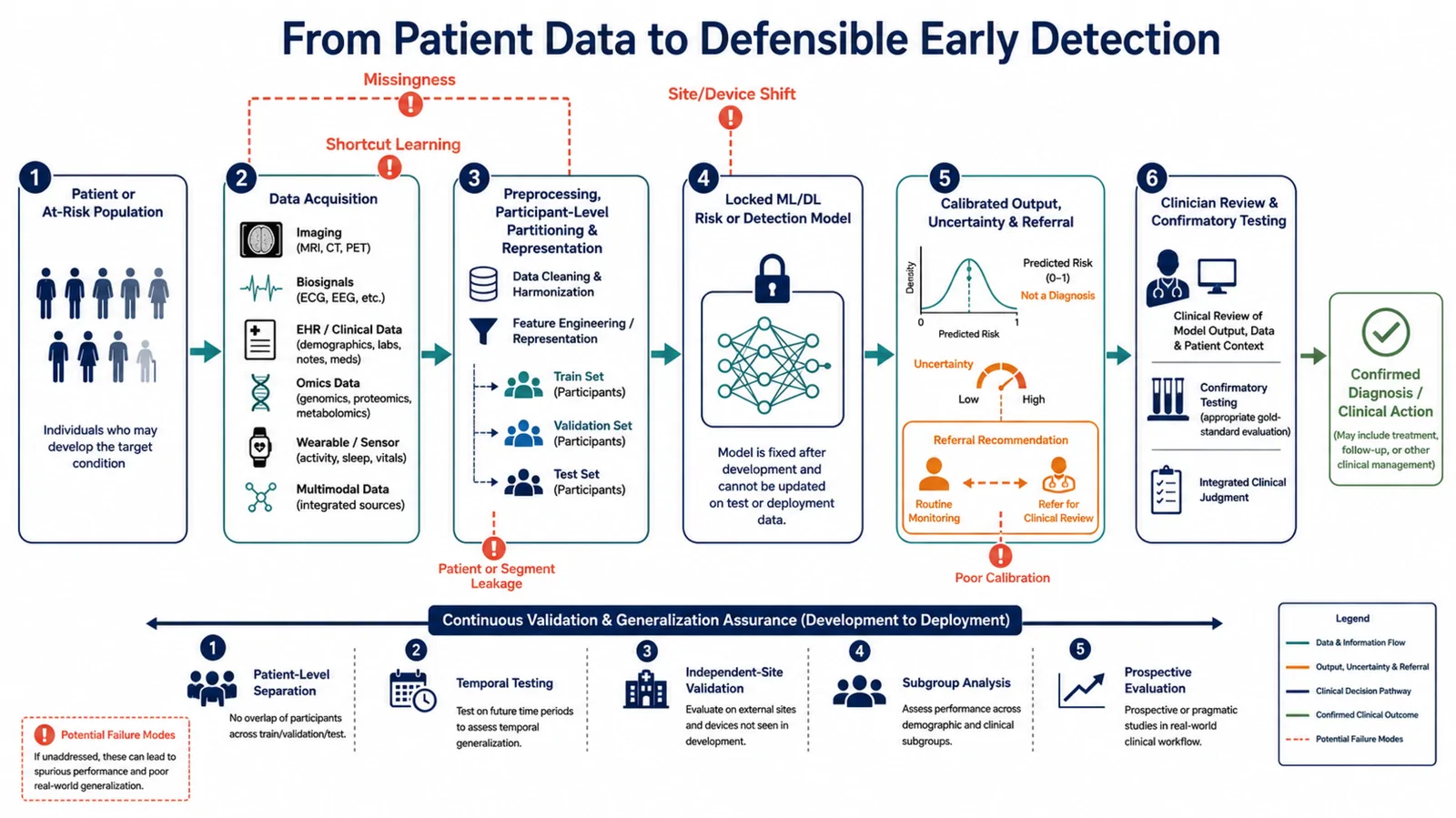
The early-detection evidence chain. A model score becomes clinically meaningful only after its target, validation setting, uncertainty, referral rule, and confirmatory pathway are specified.

Figure 1 summarizes this evidence chain and shows why model performance must be interpreted together with the clinical target, validation setting, uncertainty, and downstream action.

## 2. Positioning Relative to Previous Surveys

Previous reviews provide valuable depth within individual diseases, but their disease-specific scopes cannot serve as direct substitutes for a cross-disease and cross-modality synthesis. One recent oncology review covered imaging, multi-omics, and clinical reports across several cancer types while also spanning diagnosis, prognosis, and therapy [6]. While its breadth within oncology is useful for method mapping, combining pre-diagnostic detection with post-diagnostic tasks limits direct comparison of evidence maturity across endpoints. The multi-cancer screening review in [7] focused more specifically on biomarker and imaging strategies for detecting multiple malignancies, highlighting generalizability, false positive burden, affordability, regulation, and accessibility; however, it did not cover comparable evidence chains in cardiovascular, neurological, renal, metabolic, respiratory, or infectious disease contexts.

Disease-focused surveys also reveal modality-specific issues that a broad synthesis must retain. A pancreatic cancer review organized evidence across imaging, biomarkers, and EHR-based triage, identifying limited external validation and sparse analysis of clinical utility as continuing barriers [8]. A Parkinson’s disease review covering MRI, gait, handwriting, speech, EEG, and multimodal fusion nonetheless remains organized around the clinical problem of Parkinson’s disease recognition [9]. Thus, these works are used as contextual evidence inside the relevant disease and modality sections.

The contribution of the present survey does not lie in the number of diseases or algorithms listed but in the consistent interpretation of different studies under the same sequence of questions asking what was predicted, from which information, at what time, under which sampling process, with what independence between development and evaluation, and with what evidence of clinical usefulness. Unlike disease-specific surveys, the present framework follows comparable evidence questions across imaging, biosignals, EHR and tabular data, omics, wearables, and multimodal systems. Unlike method-centered surveys, it evaluates CNNs, recurrent models, transformers, attention, generative augmentation, traditional ML, and fusion methods in relation to task definition and validation design rather than architectural category alone. Specialized reviews are not assigned artificial negative marks for topics outside their intended scope.

## 3. Foundations of Early Disease Detection

### 3.1. Operational Definitions

This survey uses the operational categories in Table 1. The categories are assigned from the actual experimental endpoint rather than from motivational language in the title or introduction. Thus, a paper may be relevant to the broader diagnostic literature, but excluded from strict early-detection comparisons.

### 3.2. The Unit of Independence

The statistical unit used to split the data determines whether test performance represents unseen patients or merely unseen samples. Repeated images, signal windows, visits, or augmented variants from one person share biological and acquisition-specific information. If these correlated samples appear in both training and testing, the model can recognize participant-specific structure without learning a transportable disease signal.

Liu and colleagues used cross-subject and nested cross-validation for EEG-based detection of mild cognitive impairment [10]. Their model achieved 90.97% accuracy, 86.87% sensitivity, 94.76% specificity, and an AUC of 0.950 under this design [10]. The subject-level structure makes the estimate more defensible than a random split of EEG segments, although a public single-dataset evaluation still cannot measure site or device shifts.

A voice-based Parkinson’s study used subject-level nested cross-validation while applying oversampling only within the training folds [11]. Accuracy ranged from 0.827 to 0.868 across the evaluated configurations, while the best sex-specific models differed in both classifier and selected acoustic features [11]. These values are lower than many reports based on random recordings, yet the design better isolates performance on unseen participants. The study also shows that leakage control and demographic stratification can change the scientific conclusion from which model is most accurate to which signal is reproducible for which subgroup.

### 3.3. Discrimination, Calibration, Utility, and Outcome

Discrimination measures how well scores rank positive and negative cases. Calibration measures whether predicted probabilities correspond to observed risk. Clinical utility depends on the consequences of acting at a threshold. Improved patient outcome requires evidence that using the model changes care beneficially. These four properties are related but not equivalent.

An opportunistic Alzheimer detection study compared three ensemble decision strategies across three structural-MRI architectures and evaluated both balanced accuracy and calibration error [12]. Weighted averaging produced the best combination for two architectures, but even the improved balanced accuracy ranged from 0.371 to 0.608 across architectures [12]. The calibration errors also remained architecture-dependent after ensembling [12]. This result is important because it resists the common assumption that ensembling automatically makes a model clinically reliable.

Uncertainty can also be connected to workflow rather than being reported as an isolated statistic. A self-supervised diabetic retinopathy system used Monte Carlo dropout to refer uncertain predictions for expert review [13]. The referral module captured 75% of misclassified cases, while the system reported a macro-F1 score of 98.1% and an AUC of 99.2% across test folds [13]. Patient-level separation and an independent private held-out set strengthen the evaluation, but a referral percentage does not reveal clinical value unless referral volume, missed disease cost, and specialist capacity are assessed at the intended prevalence.

**Figure 2:**
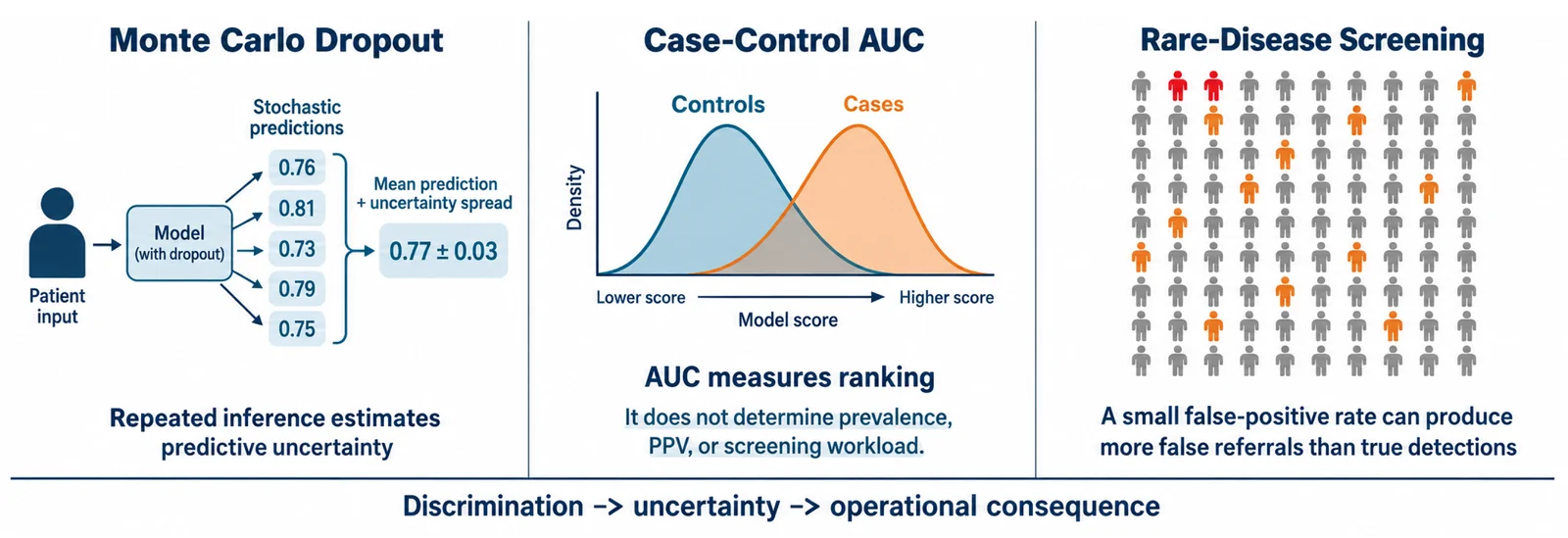
Three distinct evaluation concepts. Repeated stochastic inference can support uncertainty-aware referral, case–control AUC measures ranking under sampled cases and controls, and rare disease screening requires prevalence-aware interpretation of false positives and predictive values. Navy and blue denote model outputs, uncertainty summaries, and the control distribution; orange denotes cases in the case–control panel and false-positive referrals in the screening panel; red identifies true disease cases; and gray represents unaffected individuals. The colors are schematic and do not indicate comparative performance.

Monte Carlo dropout retains dropout during inference and repeats the same prediction several times. Variation across the resulting scores provides an operational approximation of predictive uncertainty, but the estimate is useful only if uncertainty separates difficult or shifted cases and supports a prespecified referral rule. A case–control AUC answers a different question: it measures ranking between sampled cases and controls, and is insensitive to the prevalence imposed by that sampling design; as such, it does not determine the positive predictive value or workload of population screening. Rare disease evaluation makes this distinction especially important, as even a small false positive rate can generate many more false referrals than true detections when prevalence is low. Figure 2 contrasts these three evaluation concepts and their operational implications.

## 4. Data Modalities and Their Clinical Meaning

**Figure 3:**
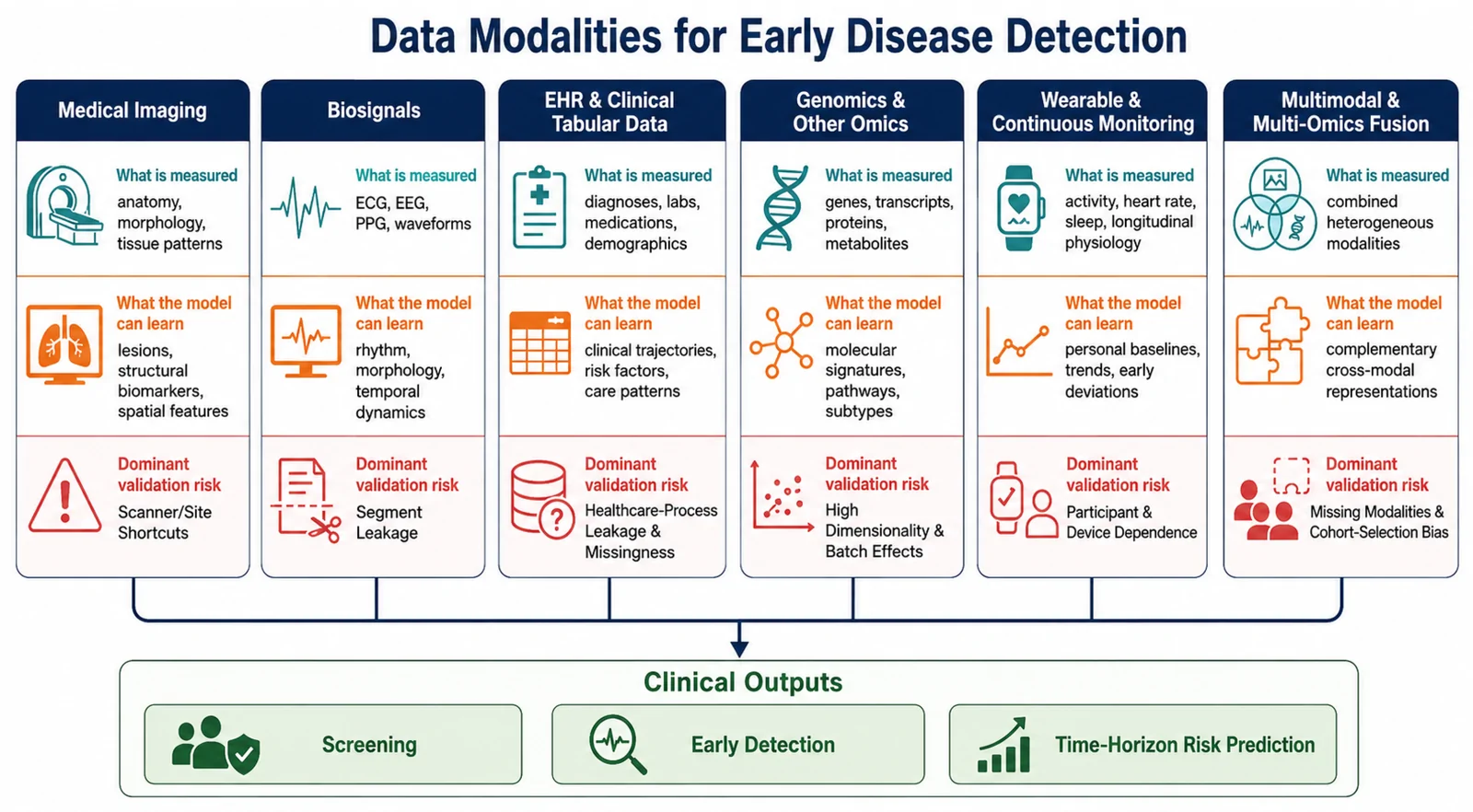
Clinical meaning and principal validation risk of the six data modality families considered in this survey.

**Figure 4:**
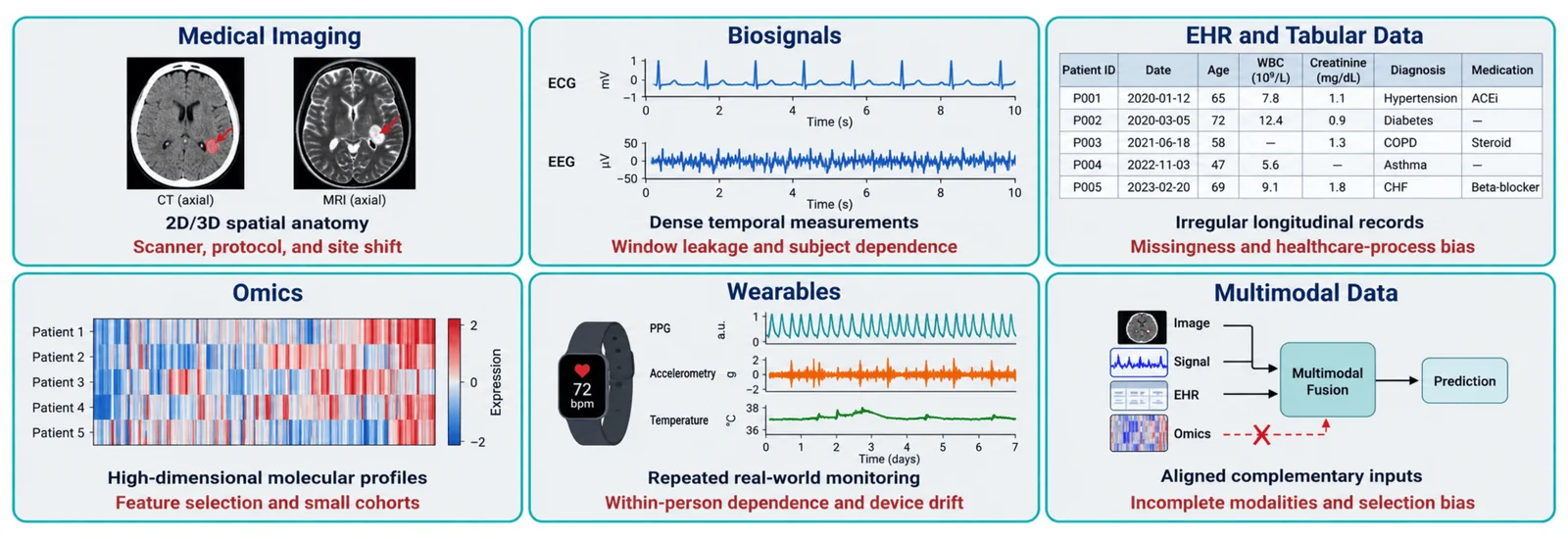
Representative schematic examples of the six modality families. Imaging preserves spatial anatomy; biosignals and wearables contain temporal dependence; EHRs are irregular and incomplete; omics data are high dimensional; and multimodal systems require aligned, jointly available inputs. Cyan outlines separate the modality panels, navy text identifies modality classes, and red text highlights major methodological risks. The red arrows in the CT and MRI examples indicate illustrative regions of interest rather than confirmed diagnoses. Blue traces represent ECG and EEG signals; teal, orange, and green traces distinguish PPG, accelerometry, and temperature measurements, respectively. In the omics heatmap, blue indicates lower standardized expression, white indicates values near zero, and red indicates higher standardized expression. Black arrows indicate the ordinary flow of available inputs, whereas the red dashed pathway and cross in the multimodal panel illustrate a missing modality and the need for missing-modality handling before fusion. Colors do not indicate comparative performance. No real patient records are displayed.

Figure 3 organizes the six modality families by their clinical meaning and principal validation risk, whereas Figure 4 illustrates the corresponding data structures. The figures serve complementary purposes, with the first presenting an interpretive taxonomy and the second a schematic data example.

### 4.1. Medical Imaging

Medical imaging supplies spatially organized measurements whose diagnostic meaning depends on anatomy, acquisition protocol, reconstruction, and selection of the region presented to the model. DL is well suited to learning hierarchical spatial representations, but can also exploit scanner signatures, annotations, cropping conventions, or acquisition artifacts. External evaluation across institutions is particularly important when the intended use extends beyond a single archive.

Deep-LIBRA separates breast segmentation from radiomic estimation of dense tissue, combining DL with engineered superpixel and radiomic analysis [1]. The method’s density estimates correlated strongly with an expert reader on independent data, with a Spearman coefficient of 0.90 [1]. Its adjusted case–control AUC of 0.612 exceeded the evaluated alternative density methods, for which the AUCs ranged from 0.528 to 0.599 [1]. The contribution consists of a reproducible intermediate phenotype evaluated against both expert assessment and downstream risk discrimination.

Imaging results become harder to interpret when the target is defined using the same visual evidence available to the model. A segmentation or lesion classification system may accurately reproduce labels derived from images without demonstrating earlier recognition than clinical interpretation. The study must specify whether AI detects a pre-diagnostic signal, assists routine interpretation, grades an established lesion, or predicts future disease.

### 4.2. Biosignals and Physiological Measurements

Biosignals encode temporal and frequency-domain dynamics that may not be visible in static clinical variables. Their density creates multiple windows per participant, which increases the apparent sample size without increasing the number of independent people. Validation must preserve participant independence before any windowing, augmentation, or feature selection.

STCWTNet transforms multichannel EEG into a sparse three-dimensional matrix that preserves electrode topology before applying spatiotemporal learning and channel-wise attention [10]. An attention-driven PageRank procedure was added to map learned channel relationships to interpretable brain region patterns [10]. Ablation evidence supports the contribution of the architectural modules, but biological agreement of attention maps does not independently validate the causal relevance of the highlighted regions.

Voice analysis illustrates why conventional ML can remain appropriate. The Parkinson’s voice study compared random forests, neural networks, naive Bayes, logistic regression, AdaBoost, and radial-basis SVM models after Boruta or minimum-redundancy maximum-relevance feature selection [11]. Sex-specific models selected different acoustic feature families, indicating that a single pooled representation could obscure subgroup-dependent signal structure [11]. The study’s strongest contribution is its control of subject leakage and imbalance.

### 4.3. Electronic Health Records and Clinical Tabular Data

EHR models learn from measurements generated by both disease and clinical care. Test ordering, visit frequency, missingness, and referral patterns can become predictors. A model may provide good performance while actually detecting the healthcare process surrounding suspected disease instead of the biological precursor. Temporal alignment must ensure that every feature was available before the intended prediction time.

The temporal diabetic retinopathy study generated features from longitudinal EHR trajectories extending up to 20 years [3]. Its EHR-only AUROC of 0.881 indicates that longitudinal clinical history contained substantial information even without the retinal image [3]. Combining EHR history with fundus imaging increased AUROC to 0.988, but the study requires external evaluation to determine whether this gain persists under different documentation and imaging practices [3].

An explainable liver fibrosis model used readily available demographic, anthropometric, and clinical variables for population pre-screening [14]. XGBoost obtained a test AUC of 0.816 in the UK Biobank-derived evaluation along with external AUCs of 0.746 in NHANES and 0.793 in an independent real-world cohort [14]. The multi-cohort decline quantifies dataset shift, although the use of different reference definitions across cohorts complicates direct equivalence of the estimates.

A multicenter pulmonary embolism study compared fourteen algorithms using physical examination findings and routine blood tests from 8757 patients [15]. XGBoost achieved an external validation AUC of 0.8875 and was compared with conventional Wells score assessment [15]. The clinically meaningful comparator and independent evaluation strengthen the result, but retrospective sampling among patients already suspected of pulmonary embolism limits inference about population screening before suspicion.

### 4.4. Omics and Molecular Measurements

Omics data can capture molecular variation before a macroscopic phenotype becomes detectable. The central difficulty is that the number of molecular variables often greatly exceeds the number of independent participants. Feature selection, normalization, batch correction, and hyperparameter tuning must be confined to the training data.

In [16], a transformer architecture integrated cell-free DNA and cell-free RNA sequencing for preterm birth risk prediction. The individual cfDNA and cfRNA models achieved test AUCs of 0.822 and 0.851, whereas joint integration achieved 0.890 [16]. Because all three estimates came from the same task and test design, the improvement provides a more interpretable fusion comparison than cross-study accuracy ranking. External validation was not established in the analyzed evidence, so the results support within-cohort multimodal complementarity rather than population transportability.

### 4.5. Wearable and Continuous Monitoring Data

Wearable sensors replace sparse clinic snapshots with repeated measurements across daily contexts. This improves temporal coverage but introduces device placement, adherence, firmware, environment, and participant behavior effects. Repeated observations also make random sample splits particularly vulnerable to identity leakage.

The WATCH-PD study illustrates how year-long multicenter consumer sensor data can support digital phenotyping while still requiring participant-level validation and cautious interpretation of screening generalizability [17]. A detailed analysis is provided in the dedicated wearable neurological subsection.

### 4.6. Multimodal Data Characteristics and Integration Requirements

Multimodal learning is justified when modalities contribute complementary information available at the intended decision point. Fusion is not automatically beneficial, as requiring every modality can reduce the eligible cohort and select patients with more intensive care requirements. Therefore, missing-modality behavior is a part of clinical validity.

The coronary artery disease framework in [4] combined CCTA, ECG, clinical variables, and laboratory measurements through weighted fusion. Its external AUC of 0.83 remained below the internal value of 0.89, while the reported ability to tolerate up to 50% missing data addressed a practical deployment constraint [4]. The result supports multimodal robustness more strongly than a complete-case experiment would, although the exact missingness mechanisms must match those encountered in use.

The IMBALMED Alzheimer framework combined clinical assessments, neuroimaging phenotypes, biospecimens, and participant characteristics through classifier diversity and class-balancing strategies [2]. The evaluation separated binary diagnosis, ternary diagnosis, and four future-oriented early detection tasks [2]. External evaluation using a later ADNI phase supported the 12-month task, but using another phase of the same overarching research program may not reproduce the institutional and demographic shift of a fully independent health system.

The diabetic retinopathy fusion study offers a different form of complementarity, since longitudinal EHR features encode systemic disease history while fundus images encode current retinal manifestation [3]. The difference between the EHR-only and fused AUROCs suggests that the modalities answer overlapping but nonidentical questions [3]. A clinically useful deployment design should determine whether EHR risk first triggers imaging or whether both modalities are always available, as these workflows imply different populations, costs, and error consequences.

## 5. Traditional Machine Learning Methods

Traditional ML remains scientifically important in early detection because many clinically realistic datasets contain hundreds or thousands of patients rather than millions of independent observations. Logistic regression, support vector machines, decision trees, random forests, gradient boosting, nearest-neighbor classifiers, and shallow neural networks can operate on compact representations with substantially fewer free parameters than large DL architectures. Their usefulness depends less on their age than on whether the representation preserves information relevant to the target without importing leakage from preprocessing or feature selection.

### 5.1. Feature Engineering as an Explicit Clinical Hypothesis

Feature engineering converts raw measurements into variables that encode a hypothesis about disease manifestation. In ECG analysis, these variables may include intervals, waveform morphology, spectral energy, or heart rate variability. In voice analysis, they may include cepstral, wavelet, perturbation, and prosodic measurements. In tabular clinical data, feature engineering may construct trajectories, ratios, rates of change, or clinically interpretable risk factors. This explicitness can be advantageous when the cohort is small and when clinical review of the decision pathway is required.

The value of explicit features is demonstrated by the subject-level Parkinson voice analysis, where Boruta and minimum-redundancy maximum-relevance selection were evaluated across multiple feature counts before comparing six classifiers [11]. The strongest configurations differed between sex-agnostic, sex-aware, male-only, and female-only cohorts [11]. This variation implies that the feature selection problem formed part of the biological and demographic question.

The liver fibrosis study provides a second example in which XGBoost was trained on readily obtainable anthropometric and clinical variables rather than latent image features [14]. External AUCs of 0.746 in NHANES and 0.793 in a real-world cohort remained clinically plausible rather than approaching the near-perfect results reported in many small benchmark datasets [14]. The reduction from internal performance exposes the cost of transport and makes the model easier to position as a pre-screening instrument.

Feature construction can also use longitudinal history. The EHR component of the temporal diabetic retinopathy framework derived thousands of variables from changing clinical measurements over time [3]. Its AUROC of 0.881 indicates that the engineered temporal representation contained information not captured by a single contemporaneous record [3]. The critical question is whether these trajectories were fully available at the intended prediction time, because using measurements recorded after referral would convert early prediction into retrospective reconstruction.

### 5.2. Linear Models, Support Vector Machines, and Tree Ensembles

Linear models provide a valuable reference because they test whether a complex nonlinear architecture is needed. A well-regularized logistic model can be competitive when the signal is approximately additive or when sample size limits reliable interaction learning. While its coefficients are not necessarily causal explanations, they can offer a transparent functional form and a defensible baseline.

Support vector machines remain suitable for moderately sized high-dimensional feature spaces. The externally validated complete blood count study compared six previously developed COVID-19 models across two hospitals and found that the SVM produced an average AUC of 97.5%, sensitivity of 87.5%, and specificity of 94% [18]. The same model was also reported as the best calibrated among the evaluated alternatives [18]. This result is more informative than a within-site algorithm contest because it evaluates whether a compact hematological representation travels between hospitals.

Tree ensembles are effective when clinical variables exhibit nonlinear thresholds, interactions, and missingness patterns. The multicenter pulmonary embolism study compared fourteen algorithms and selected XGBoost using an external cohort rather than internal fit alone [15]. The resulting external AUC of 0.8875 was compared with the Wells score, providing a clinically recognizable baseline [15]. The study sampled patients already suspected of pulmonary embolism, so its result supports triage within a diagnostic pathway rather than population-wide prediction.

Rare disease analysis shows why high accuracy cannot be the only criterion. The multicenter tyrosinemia study contained 219 longitudinal records from 35 patients, including only eight with hepatocellular carcinoma [19]. XGBoost identified alanine transaminase as a potential precursor of alpha-fetoprotein alteration, but the reported AUROC of 0.73 reflects the uncertainty inherent in the small cohort [19]. The study is valuable as hypothesis generation, while the limited number of independent patients prevents a strong deployment claim.

### 5.3. Imbalance, Resampling, and Thresholds

Early detection datasets are often imbalanced because disease prevalence is lower than the prevalence created in retrospective case–control benchmarks. Resampling can improve optimization, but applying it before the data split duplicates information across training and evaluation. The appropriate procedure is to split independent patients first and perform all resampling only inside the training folds.

The Parkinson’s voice study applied adaptive synthetic sampling within training folds and retained subject-level nested cross-validation [11]. This design separates the benefit of balancing from leakage through synthetic neighbors. Matthews correlation coefficients remained moderate despite accuracy results ranging from 0.827 to 0.868, illustrating how a balance-sensitive metric can reveal limitations hidden by accuracy [11].

Decision thresholds should be chosen according to the clinical action. A population screening threshold may prioritize sensitivity and negative predictive value, whereas a confirmatory model may prioritize specificity and positive predictive value. Threshold selection on the test set inflates performance and makes utility appear greater than it is. A valid design chooses the threshold in training or validation data and preserves the test set for a single final evaluation.

### 5.4. When Traditional ML Is Preferable

Traditional ML is preferable when the cohort is small, variables are clinically meaningful, and the expected relationship can be represented without extensive spatial or temporal learning. It also provides an essential baseline when DL is proposed for tabular data. If a transformer or multilayer network does not outperform a tuned gradient-boosting model under the same patient split, then the more complex architecture has not demonstrated added predictive value.

Traditional ML can also support low-resource deployment. The premature brain injury study compared fourteen algorithms before selecting CatBoost and reducing the model to seven features [20]. The final model achieved an AUC of 0.8229 on an independent test set and was accompanied by a deployable prediction tool [20]. Although the study was described as a prospective cohort, its evidence remains a model development evaluation rather than a prospective test of model-guided care.

## 6. Deep Learning and Representation Learning

DL changes early detection pipelines by learning representations jointly with the prediction objective. This is particularly useful for images, waveforms, longitudinal sequences, and multimodal inputs for which the informative structure cannot be summarized easily by a small set of handcrafted features. The same flexibility increases the opportunity to learn acquisition artifacts, patient identity, site signatures, or label generation shortcuts.

### 6.1. Convolutional Architectures

Convolutional neural networks (CNNs) encode local spatial or temporal structure through shared filters. Their inductive bias is well matched to radiographs, histopathology patches, dermoscopic images, retinal photographs, and one-dimensional physiological signals. Transfer learning reduces optimization difficulty when labeled medical cohorts are small, but pretraining does not correct sampling bias or label error.

The large number of imaging studies synthesized later in this survey shows that CNN performance is often reported on public datasets with repeated reuse of the same images. Accuracy on these datasets can indicate technical progress under a fixed benchmark, but cannot establish robustness to scanner, site, or population shift. Near-perfect values should receive particular scrutiny when patient-level splitting, independent testing, and tuning boundaries are not reported.

MammoDG directly addresses cross-domain mammography by combining cross-view interaction with instance-level contrastive regularization [21]. Its contribution targets the degradation caused by imaging protocol and center variation. The study is methodologically closer to the deployment problem than, for instance, an architecture evaluated on a random split of one mammography archive.

DualA-Net addresses a different question by using a dual-branch encoder, multi-scale skip connections, and an adaptive receptive-field decoder for medical image segmentation [22]. Its evaluation across five datasets representing different segmentation tasks supports technical versatility and efficiency, but does not constitute independent external clinical validation. The citation is retained because segmentation is a recurrent intermediate operation in early detection pipelines; the study is not presented as direct evidence that the architecture improves screening decisions or patient outcomes.

### 6.2. Recurrent and Temporal Models

Recurrent neural networks, long short-term memory networks, and gated recurrent units model ordered observations. They are applicable to ECG sequences, EEG recordings, EHR trajectories, wearable time series, and repeated laboratory measurements. The principal risk is that a model may learn the observation process rather than the disease process, especially when measurement frequency changes after clinical suspicion.

Temporal models should be evaluated using a prediction index time. Features recorded after that index must be excluded, and patients should be separated before creating overlapping windows. Temporal validation should train on earlier patients and evaluate on later patients when the intended use will encounter changes in practice. Random cross-validation cannot reproduce such drift.

The temporal diabetic retinopathy framework is notable because its EHR branch represents long-term changes rather than a static visit [3]. The multimodal branch then combines this history with retinal imaging [3]. The design supports a clinically plausible distinction between identifying high-risk patients from records and assessing current retinal manifestation from images, although external temporal transport remains untested in the analyzed evidence.

### 6.3. Transformers and Attention

Transformers model long-range dependencies through attention. In imaging, attention can connect distant anatomical regions or image patches. In biosignals, it can relate separated time points or channels. In multimodal systems, cross-attention can condition one modality on another. These capabilities are useful only when cohort size and regularization are sufficient to estimate the additional parameters.

STCWTNet combines a topology-aware spatiotemporal module with channel-wise transformer encoding for MCI detection from EEG [10]. Its transformation of electrodes into a topology-preserving sparse matrix gives the attention mechanism a physiological organization rather than treating channels as an arbitrary sequence [10]. Nested cross-subject evaluation supports internal subject generalization, but a new EEG system or clinical center would still test a different source of variation.

The preterm birth study used transformer-based integration of cfDNA and cfRNA sequence information [16]. The fused AUC of 0.890 exceeded the cfDNA value of 0.822 and the cfRNA value of 0.851 under the same test setting [16]. This within-study comparison supports complementary molecular information, but does not necessarily establish that the transformer would be superior to a well-tuned classical integrator.

The evidence does not support a universal advantage of transformers over CNNs. CNNs impose locality and translation-related inductive biases that are efficient for many medical images and one-dimensional signals, whereas transformers can model longer-range interactions but generally introduce greater data, regularization, and computational demands. An attention module added to a CNN should also be distinguished from a transformer architecture, as attention can reweight features without replacing convolutional representation learning. Consequently, claims of superiority are credible only when CNN, transformer, and hybrid alternatives are trained on the same cohort, use the same split and preprocessing, and receive comparable tuning effort. Improvements observed across different datasets cannot isolate the contribution of architectural complexity.

### 6.4. Self-Supervision, Transfer Learning, and Generative Augmentation

Self-supervised learning uses unlabeled data to learn representations before task-specific training. This is valuable in medical domains where acquisition is abundant but expert labels are limited. The benefit must be separated from differences in pretraining data, augmentation, model size, and tuning effort.

The uncertainty-aware diabetic retinopathy study used a two-stage self-supervised warm start followed by a binary-to-five-class curriculum [13]. Its reported 2.1 percentage-point improvement over ImageNet initialization provides a direct pretraining baseline [13]. Patient-level separation and independent private testing improve credibility, while the extremely high fold-average performance still requires prevalence-aware evaluation in an actual screening population.

Conventional augmentation applies label-preserving transformations such as rotation, reflection, cropping, scaling, intensity perturbation, or signal-domain noise. Its validity depends on whether the transformation remains physiologically or anatomically plausible for the clinical target. Generative augmentation instead learns a data distribution and produces or reconstructs observations through models such as GANs or VAEs. GANs can create visually sharp samples through adversarial training, whereas VAEs learn a probabilistic latent representation that may also support dimensionality reduction or generation. These approaches should not be treated as interchangeable forms of sample enlargement.

Generative augmentation introduces risks that conventional transformations do not fully share. A generator can reproduce site artifacts, memorize training examples, collapse toward a narrow subset of appearances, or create structures that appear realistic but are clinically inconsistent. Leakage occurs if the generator is fitted prior to patient-level partitioning or if synthetic descendants of held-out observations enter training. A performance increase on the source dataset only establishes optimization benefit; further diversity audits, nearest-neighbor checks, patient-level isolation, and evaluation on an independent real cohort are needed to support generalization claims.

The lightweight domain-generalized segmentation work in [23] evaluated a modified pix2pix architecture across prostate micro-ultrasound and contrast-enhanced liver MRI. Its reduced training time and cross-modality segmentation results address efficiency, although the use of two tasks does not prove generalization to unseen clinical domains. In [24], a deep convolutional and variational autoencoder network used a learned latent representation for cervical cancer prediction and classification. This use of a VAE illustrates representation learning as well as generation, so its performance cannot be attributed to synthetic sample count alone. A Parkinson’s drawing study compared conventional and GAN-based augmentation on a small set of 82 drawings [25]. The finding that conventional augmentation provided the stronger practical configuration demonstrates that a more complex generator does not guarantee a more useful disease representation.

**Figure 5:**
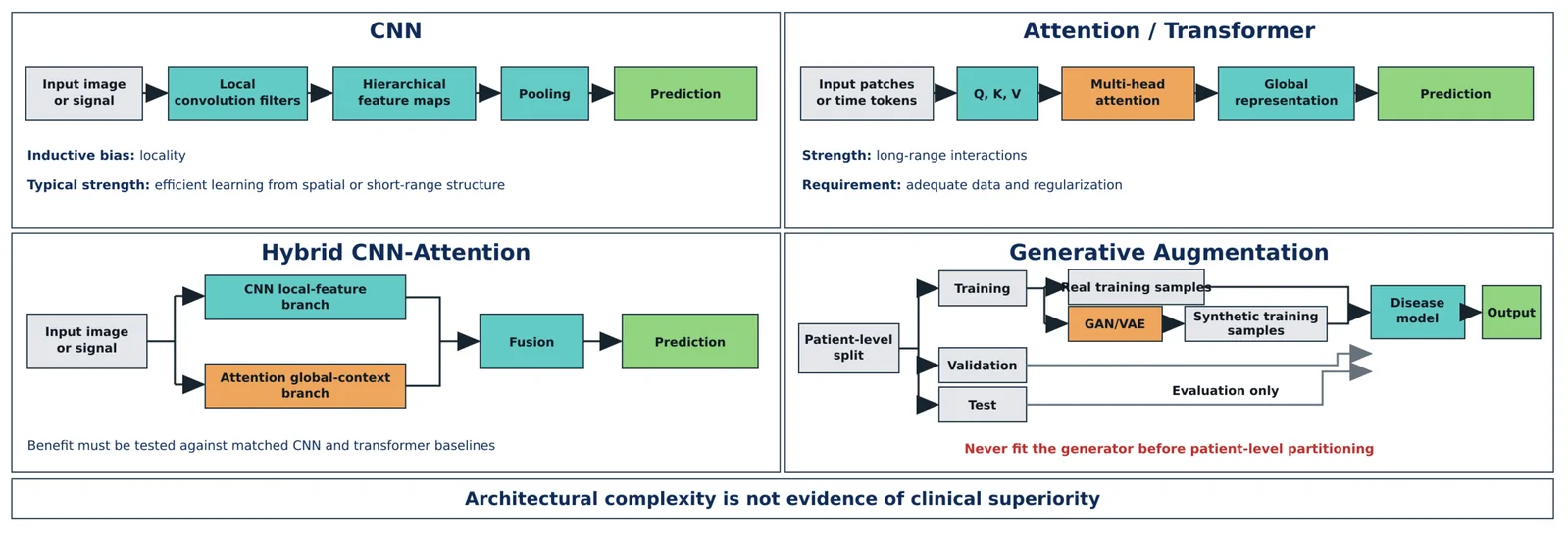
Selected representation patterns discussed in the survey. CNNs emphasize local structure; attention and transformers model feature interactions; hybrid systems combine local and global branches; and generative augmentation must remain confined to the training partition. The diagram only describes information flow, and is not an accuracy ranking.

Figure 5 summarizes the information flow and principal assumptions of the CNN, attention/transformer, hybrid, and generative augmentation patterns discussed above. It is intended as a structural comparison rather than a performance ranking.

### 6.5. Graph Learning and Multimodal Fusion

Graph neural networks represent relationships between brain regions, molecular entities, visits, patients, or anatomical structures. Their value depends on whether graph edges encode meaningful relationships available at deployment. A graph built using labels or the complete dataset can leak test information even when node features are separated.

Multimodal fusion can occur at the input, representation, decision, or temporal level. Early fusion assumes aligned and jointly available measurements. Late fusion can tolerate independently trained branches but may miss fine cross-modal interactions. Cross-attention offers adaptive interaction, yet it can overfit when the number of complete multimodal patients is small.

LiteCrossFusion combines 3D MRI with clinical features using cross-attention and reports only 1.3 million parameters [26]. Grad-CAM and SHAP are applied to the imaging and clinical branches, while uncertainty estimation is included in the architecture [26]. The framework illustrates a useful design direction, but evidence from the PPMI dataset alone cannot confirm transport across scanners and clinical populations.

## 7. Cross-Study Synthesis and Evidence Consistency

### 7.1. Classification and Segmentation

Classification and segmentation answer different questions and require different evidentiary chains. Classification reduces an examination to a class, probability, or risk score, whereas segmentation estimates the spatial extent of an anatomical or pathological region. A high Dice score demonstrates overlap with a reference mask but does not show that the mask changes patient-level sensitivity, referral decisions, or outcomes. DualA-Net demonstrates technical versatility across five segmentation datasets, yet its evaluation does not establish an early screening benefit [22]. Segmentation should be treated as an intermediate component unless the study also evaluates the downstream clinical decision.

The above distinction also changes what constitutes an adequate comparison. A segmentation model should be compared under identical annotation policies and report the boundary or overlap error together with failure cases involving small, diffuse, or low-contrast lesions. A classification model should be evaluated at a clinically meaningful operating point, and at the patient level when several images belong to one patient. When segmentation supplies a classifier or measurement pipeline, the analysis should propagate segmentation errors into the final decision rather than evaluating the mask in isolation. This end-to-end view prevents technical improvements in one module from being mistaken for clinical improvement in the complete pathway.

### 7.2. Conventional Augmentation and Label Preservation

Conventional transformations are most defensible when they preserve the target anatomy, physiology, acquisition geometry, and label semantics. A rotation that is harmless for a dermoscopic image may be implausible for an upright chest radiograph, while time warping that is useful for one physiological signal may alter a diagnostic interval in another. As such, augmentation policy forms part of the clinical task definition rather than a generic recipe. All transformations must be fitted or sampled after patient-level partitioning so that correlated descendants cannot cross between training and evaluation sets.

The Parkinson’s drawing study provides a useful matched comparison because conventional augmentation produced the stronger practical configuration despite explicit evaluation of GAN-generated data [25]. This result shows that greater visual novelty and architectural complexity do not guarantee label-preserving variation. Conventional augmentation remains a strong baseline because its transformation space is explicit, inspectable, and easier to constrain using domain knowledge.

### 7.3. Generative Augmentation: Fidelity, Diversity, and Leakage

Generative augmentation adds a learned data-producing system in which errors can be inherited by the disease model. A meta-analysis of adversarial networks in cancer imaging found substantial heterogeneity in tasks, evaluation practices, and evidence used to support synthetic-data claims [27]. A broader healthcare review likewise identifies fidelity, utility, privacy, and diversity as separate evaluation dimensions rather than interchangeable indicators of synthetic-data quality [28]. A visually realistic synthetic image can be redundant, memorized, clinically inconsistent, or unhelpful for the decision boundary.

A defensible generative pipeline partitions patients before fitting the generator, restricts generator training to the development partition, checks near-duplicate and nearest-neighbor similarity, assesses coverage of clinically relevant subgroups, and evaluates the final disease model on untouched real patients. Improvement on a source-cohort test set demonstrates an optimization effect, not the creation of new independent clinical evidence. External real-data evaluation is especially important because a generator may amplify site-specific acquisition signatures while appearing to increase sample diversity.

### 7.4. CNNs, Attention, and Transformers

The reviewed evidence supports conditional rather than universal architectural conclusions. CNNs encode local structure efficiently and remain strong when local morphology dominates or training cohorts are limited. Attention can reweight features within a CNN, whereas a transformer uses attention as a central representation mechanism; these designs should not be grouped as if they made the same assumptions. While transformers can connect distant image regions, channels, time points, or modalities, this flexibility increases dependence on sample size, regularization, pretraining, and tuning.

A systematic review comparing vision transformers with CNNs in medical imaging found that conclusions depend strongly on the task, dataset, pretraining, and experimental design [29]. STCWTNet offers a physiologically organized use of attention by preserving EEG electrode topology, but its evidence remains internal to the evaluated source [10]. The cfDNA–cfRNA study provides a stronger within-study argument for fusion, as the integrated AUC of 0.890 exceeded both single-modality values under the same test setting [16]. However, none of these findings implies that architectural complexity alone produces better clinical performance.

### 7.5. Within-Study Comparisons and Claims of Superiority

Architecture attribution is credible only when competing models use the same patients, endpoint, preprocessing, split, optimization budget, and evaluation procedure. Cross-paper comparisons confound architecture with cohort spectrum, prevalence, label quality, image resolution, data cleaning, and test set reuse. The most informative comparisons report tuned classical, CNN, transformer, and hybrid baselines within one protocol and include uncertainty around their differences. A small matched gain with external confirmation is stronger evidence of architectural value than a large numerical gap assembled from unrelated datasets.

### 7.6. Consistency Across Datasets and Clinical Tasks

Reported gains are not consistently reproduced across datasets and clinical tasks. The ECG-based CKD model declined from an internal AUC of 0.885 to an external AUC of 0.861 [5]. The multimodal coronary artery disease model similarly declined from 0.89 internally to 0.83 externally [4]. These reductions are not methodological failures; they quantify transport difficulty that an internal split cannot expose. Near-perfect performance on one convenience dataset provides no corresponding estimate of robustness to scanner, site, prevalence, or workflow shift.

A claim of consistent improvement should require the same direction of effect across independent cohorts, comparable endpoints, clinically relevant operating points, and patient-independent evaluation. Stability of AUROC alone is insufficient if sensitivity falls at the threshold used for referral, calibration deteriorates, or the model abstains on a growing fraction of cases. Consistency should also be separated by task, as an architecture may be reliable for lesion localization yet unstable for patient-level screening, or may be useful for retrospective ranking yet poorly calibrated for prospective risk. This definition shifts synthesis from counting favorable studies to identifying where, for whom, and under which validation conditions an improvement persists.

Table 2 consolidates these recurring strengths, limitations, and evidence boundaries across the principal method families.

## 8. Comparative Evidence and Validation Context

### 8.1. Metric Interpretation Across Heterogeneous Clinical Tasks

Accuracy, sensitivity, specificity, precision, F1 score, AUROC, AUPRC, Dice score, and calibration answer different questions. Their numerical values depend on the clinical endpoint, class balance, decision threshold, unit of analysis, and sampling design. AUPRC is especially informative under strong class imbalance, whereas AUROC summarizes ranking over thresholds and may remain favorable when positive predictive value is operationally poor. Dice score evaluates spatial overlap and is not interchangeable with patient-level classification performance. No metric should be interpreted independently of the decision and population for which it was measured.

Discrimination and calibration can lead to different judgments about the same model. In a cohort of 1518 patients with a 72.3% prevalence of lung cancer, evaluated probability models produced AUROCs from 0.66 to 0.74, while their Brier scores and calibration behavior also differed [30]. This example shows why ranking ability cannot establish that predicted probabilities are suitable for counseling or threshold-based management. Prevalence and referral spectrum must accompany interpretation of both discrimination and calibration.

### 8.2. Dataset Size, Independence, and Validation Depth

The number of images, windows, or visits is not necessarily the number of independent observations. Thousands of segments derived from a small number of patients can produce a large nominal sample while retaining strong within-person correlation. Dataset size must be interpreted together with patient count, event count, and the unit used for splitting. Validation depth progresses from a single internal split through patient-level cross-validation and temporal testing to independent-site and prospective evaluation. These designs differ in statistical precision; in addition, they expose increasingly realistic sources of deployment variation.

TRIPOD+AI emphasizes transparent reporting of the participants, predictors, outcomes, model development process, evaluation, and intended use of clinical prediction models [31]. PROBAST+AI addresses a different question by evaluating methodological quality, risk of bias, and applicability [32]. Complete reporting makes it possible to assess a study, but does not ensure that the design is unbiased; conversely, a potentially strong design cannot be trusted if key methods remain opaque. Therefore, comparative synthesis should consider reporting completeness and evidence quality as related but distinct dimensions.

Table 3 applies this interpretation to selected anchor studies by placing performance beside sample size, validation design, and the clinical boundary of each result.

## 9. Disease-Oriented Applications

Disease-oriented interpretation is necessary because an identical model architecture can support different decisions. The sections below synthesize what the modality-specific evidence means for major clinical groups. Detailed study values are retained in the modality-specific tables presented in the corresponding sections, where they are reported as within-study results.

### 9.1. Cancer Screening and Early Oncological Detection

Cancer studies dominate the imaging literature because lesions can be localized in mammography, CT, MRI, endoscopy, dermoscopy, histopathology, and retinal-like optical fields. The clinical target ranges from precursor detection to malignancy classification of an already sampled lesion. These tasks must not be treated as equivalent.

Polyp detection is a genuine precursor screening problem when the system operates during colonoscopy before malignant transformation. The PolypGen study trained object detectors on 7900 still and video images from a multicenter dataset and externally tested on Kvasir [37]. YOLOv8x achieved an F1 score of 0.9058 and accuracy of 0.949, while EigenCAM supplied regional visualization [37]. The external dataset improves robustness evidence, but frame-level performance does not establish whether the model increases adenoma detection without unacceptable false alarms during live procedures.

A separate multi-dataset strategy trained polyp detectors on four databases and externally evaluated on ETIS-LaribPolypDB [38]. YOLOv8m achieved precision of 92.4%, recall of 85.4%, F1 of 88.7%, and mAP@0.5 of 91.1% before the external test [38]. The use of heterogeneous development data is a strength, although the reported results do not quantify the external metrics needed to measure the remaining shift.

The ClinExpPICCOLO benchmark contributes a different form of evidence by using 65 unedited endoscopic images and establishing performance from 146 gastroenterologists [35]. Expert accuracy was 77.74%, sensitivity was 86.47%, and specificity was 74.33% [35]. While this benchmark does not replace a clinical trial, it does supply a human reference under images designed to resemble clinical localization difficulty.

For indeterminate pulmonary nodules, the clinically relevant question is not ordinary nodule classification but whether invasive work-up can be targeted safely. A two-path model integrated CT, clinical variables, and blood biomarkers using 1284 development patients and three external cohorts of 155, 136, and 96 patients [33]. External AUCs ranged from 0.712 to 0.918 and exceeded the reported clinical or image-only baselines within each cohort [33]. Variation between sites remains substantial, which shows why a single pooled AUC would conceal clinically important transport differences.

Uncertainty-aware lung nodule analysis evaluated Monte Carlo dropout, deep ensembles, and their combination for referral [39]. Referral based on uncertainty increased accuracy to 0.959 after uncertain cases were removed from automated decisions [39]. This selective accuracy cannot be interpreted without reporting the retained coverage and the workload transferred to experts.

Breast imaging spans risk estimation, screening, lesion detection, and pathology classification. Deep-LIBRA measures breast density and case–control risk rather than detecting a tumor [1]. MammoDG targets cross-center screening variation through domain generalization [21]. The ultrasound uncertainty study reported 100% accuracy under five-fold cross-validation on BUSI, but such a perfect estimate from one public dataset requires independent testing before clinical interpretation [40].

Skin lesion systems similarly range from diagnosis of photographed lesions to screening of unselected patients. An uncertainty-aware skin model reported accuracy of 85.65, uncertainty accuracy of 83.03, and expected calibration error of 1.93 under its evaluation [41]. The moderate discrimination accompanied by explicit uncertainty is more informative for referral than a deterministic accuracy value alone, although external demographic validation was not established in the analyzed evidence.

Multi-cancer approaches move beyond site-specific screening by combining biomarkers or imaging signals across malignancies. The reviewed evidence identifies data sparsity, generalizability, false positives, false negatives, affordability, regulatory approval, and access as central bottlenecks [7]. A system that detects more cancer types also creates a more complex confirmatory pathway because the origin and urgency of a positive signal may remain uncertain.

### 9.2. Cardiovascular and Metabolic Disease

Cardiovascular early detection includes rhythm screening, structural or atherosclerotic risk, acute event triage, and prediction of later complications. ECG and heart sounds provide inexpensive physiological signals, while clinical records provide established risk factors and longitudinal context. The label often determines whether the system detects current disease or predicts future risk.

An ECG-based deep model for coronary calcification was developed using nearly 200,000 ECGs and evaluated across three health-check centers and the UK Biobank [42]. The model achieved AUCs of 0.741 for any calcification and 0.837 for severe calcification [42]. Reclassification analyses suggest a possible role in targeted CT or statin decisions, but the authors appropriately state that treatment implications require further clinical evaluation.

The CAD multimodal model obtained an internal AUC of 0.89 and an external AUC of 0.83 while tolerating substantial missingness [4]. Its external decline demonstrates that modality fusion does not remove site dependence. The model’s practical value will depend on whether CCTA is already available, because a system requiring CCTA cannot function as a low-cost pre-imaging screen.

Single-lead ECG arrhythmia detection is attractive for wearable deployment. The multicenter fusion model reported an overall F1 score of 99.57%, exceeding a cascade reference by 4.36 percentage points [43]. The study recognizes bias from intra-patient evaluation, but the reported results do not provide the exact inter-patient and cross-dataset values needed to assess transport.

Cardiac auscultation provides an inexpensive signal, but is sensitive to recording conditions. Data augmentation and generalization produced sensitivity of 96%, specificity of 81%, and F1 of 90% for abnormal heart sounds [44]. The imbalance between sensitivity and specificity could be acceptable for screening if the downstream false positive burden is manageable.

Metabolic and vascular risk models often use tabular variables. The liver fibrosis study demonstrates multi-cohort screening with interpretable predictors [14], while the pulmonary embolism study demonstrates external evaluation against a clinical score in suspected patients [15]. These examples show that traditional ML can generate stronger translational evidence than a higher-capacity architecture confined to one public dataset.

### 9.3. Neurological and Cognitive Disorders

Neurological early detection is difficult because disease may develop gradually, labels may be based on imperfect clinical criteria, and patients can occupy intermediate states. Binary classification between established disease and healthy controls is usually easier than separating prodromal disease from normal aging or predicting progression.

STCWTNet evaluates MCI rather than established Alzheimer disease and uses cross-subject nested validation [10]. Its sensitivity of 86.87% is lower than its specificity of 94.76%, which matters if the intended use is screening [10]. The remaining question is whether the learned EEG pattern transports across acquisition systems and clinical definitions of MCI.

IMBALMED distinguishes contemporaneous Alzheimer classification from 12-, 24-, 36-, and 48-month early detection targets [2]. This design aligns the task with a prediction horizon. External evaluation using a later ADNI phase is stronger than a random internal split, but remains closer to dataset continuation than independent healthcare deployment.

Voice-domain generalization provides an instructive negative tradeoff. Cognitive impairment accuracy decreased from 0.96 to 0.90 after the model was forced to suppress center-specific information, while center-classification accuracy fell from 0.96 to 0.64 [45]. The reduced headline accuracy is evidence that the model became less dependent on recording center, which may improve real transportability.

The Parkinson voice study similarly prioritizes subject-level evaluation and reports accuracy between 0.827 and 0.868 [11]. These values are substantially below several public-dataset reports synthesized later, but the methodological controls are stronger. This contrast supports evaluating the independence structure before comparing model names.

Wearable Parkinson’s assessment provides longitudinal multicenter evidence for digital phenotyping, but its modest cohort does not establish population screening across other movement disorders and common comorbidities [17]. The full performance and validation interpretation is presented in the dedicated wearable neurological subsection.

TransformEEG explicitly targets inter-subject generalizability in EEG-based Parkinson’s detection [46]. The importance of this objective is methodological: a model can classify recordings accurately while failing on unseen people if person-specific spectral structure is shared across splits. External device and site evaluation would extend this control beyond participants.

### 9.4. Respiratory and Infectious Disease

Respiratory and infectious disease studies frequently use chest imaging, vital signs, blood tests, or EHR time series. While rapid diagnostic assistance is valuable during an outbreak, dataset construction can encode hospital, time, or scanner information. A model that distinguishes datasets rather than diseases may appear accurate under pooled random splitting.

The complete blood count COVID-19 study externally validated six models in two hospitals and examined both AUC and calibration [18]. This design addresses transport more directly than image classification using cases and controls collected from different repositories. Privacy-aware COVID-19 prediction further showed that sensitive demographic information can be suppressed through adversarial training without a reported loss of hold-out and external generalization [47].

UncertaintyFuseNet combines CT and radiographic representations, reporting accuracy results of 99.08% and 96.35% on the respective datasets [48]. The use of ensemble Monte Carlo dropout addresses confidence, but the results still depend on whether unseen data represent independent clinical sites or merely withheld samples. Noise robustness is not equivalent to epidemiological or institutional robustness.

The pediatric abdominal sepsis study provides stronger clinical evidence. Development used 6566 retrospective patients, while prospective external evaluation included 308 patients from seven hospitals [34]. External AUC was 0.928 with a 95% confidence interval from 0.895 to 0.961 [34]. The prospective multicenter cohort and routine nine-variable input move the work beyond many retrospective sepsis models, although clinical outcome benefit still requires a workflow trial.

Malaria parasite detection illustrates the need for patient-level as well as object-level evaluation. The uncertainty-guided attention model was assessed under both parasite-level and patient-level schemes across two datasets [49]. This distinction prevents thousands of patches from being mistaken for thousands of independent patients.

### 9.5. Renal, Hepatic, Musculoskeletal, and Rare Disease Applications

CKD detection from ECG is an example of cross-organ inference. The model was developed on 49,632 patients and externally evaluated on 10,476 community hospital patients [5]. A positive screen among people without baseline CKD predicted later incident disease, which suggests a pre-laboratory risk signal rather than simple reproduction of an eGFR label [5]. The mechanism may combine systemic cardiovascular effects with kidney-related physiology, so causal interpretation should remain cautious.

The liver fibrosis study uses conventional variables and independent cohorts for population pre-screening [14]. The rare tyrosinemia analysis seeks earlier HCC warning from follow-up biomarkers, but includes only 35 patients [19]. Together, they show how evidence expectations must reflect both clinical rarity and intended scale.

In a rare disease setting, accuracy can remain high when a model predicts the majority class for most patients. Sensitivity describes the proportion of detected disease patients, whereas specificity describes the proportion of correctly excluded non-disease patients. Positive predictive value additionally depends on disease prevalence; as prevalence decreases, false positive referrals can dominate even when sensitivity and specificity appear favorable. Rather than interpreting accuracy alone, rare disease evidence should report class counts, precision or positive predictive value, AUPRC when appropriate, and uncertainty intervals.

Infrared thermography for arthritis and arthrosis achieved only about 64% overall test accuracy despite near-perfect separation of healthy from diseased participants [50]. AUC between arthritis and arthrosis remained approximately 0.60–0.68 [50]. This apparently weak result is scientifically useful because it defines the prototype as a triage tool rather than claiming replacement of conventional diagnosis.

## 10. Imaging-Based Early Detection

Medical imaging is the largest evidence group in this survey. Table 4 retains the reported study-specific values together with the disease target, method, and dataset. The table should be read horizontally within each study, not vertically as a leaderboard.

### 10.1. Preprocessing, Segmentation, and Region Selection

Preprocessing decisions determine which information reaches the model. Cropping can remove irrelevant background, but can also encode lesion location supplied by an annotator; similarly, contrast enhancement can reveal subtle structure but may amplify acquisition-specific noise. While segmentation can improve lesion-focused classification, using ground-truth masks at test time creates a workflow that may not exist clinically.

The semi-supervised breast mass segmentation study combines residual attention with uncertainty-guided mean-teacher learning [155]. Its contribution addresses limited labels and ambiguous boundaries, but segmentation remains an intermediate task. The clinical value depends on whether the automated mask improves lesion classification, reading time, or patient management.

The OCT uncertainty-guided cross-transformer focuses on difficult boundaries between retinal fluid categories [156]. Semantic uncertainty is used to guide the segmentation representation. The approach is relevant to early detection only when the segmented fluid changes screening, grading, or referral decisions.

### 10.2. Generalization Across Scanners and Sites

Cross-domain shift can arise from scanner manufacturer, reconstruction kernel, image compression, protocol, patient positioning, and local annotation practice. Randomly mixing images from all sites into training and testing measures interpolation across a known mixture, not deployment at an unseen center. Leave-one-site-out or external validation is more informative.

MammoDG uses contrastive regularization to suppress center-specific mammography variation [21]. The domain-generalized voice study shows how explicitly removing center information can lower internal accuracy while improving invariance [45]. These studies support reporting both disease discrimination and the residual ability to predict acquisition site.

The knee osteoarthritis harmonization framework was trained on the Osteoarthritis Initiative dataset and externally evaluated on a Mendeley dataset [157]. It reported 98.43% accuracy and an AUC of 99.01% after structural harmonization [157]. While the values are strong, clinical validation requires confirmation that labels, severity definitions, and patient spectrum are comparable across the two sources.

### 10.3. Uncertainty and Referral in Imaging

Uncertainty estimation can convert a closed classifier into a selective system that refers difficult cases. The important outcome is the risk–coverage relationship, that is, performance among automated cases as coverage decreases. Reporting only the improved accuracy after referral omits the cost imposed on clinicians.

The lung CT uncertainty study compared Monte Carlo dropout, deep ensembles, and ensemble Monte Carlo dropout [39]. The vertebral metastasis study likewise used ensemble Monte Carlo dropout on 11,468 abdominal CT examinations [158]. Hercules evaluates an uncertainty-aware hierarchical fusion architecture across retinal OCT, lung CT, and chest radiography [159]. Cross-dataset breadth supports technical versatility, but each disease still needs calibration and referral evaluation at its clinical prevalence.

## 11. Biosignal-Based Early Detection

Biosignals provide temporally dense evidence from electrical, acoustic, hemodynamic, and motor processes. Table 5 summarizes the studies assigned primarily to this modality.

Signal preprocessing can become part of the learned shortcut. Filtering, beat extraction, artifact rejection, time–frequency transformation, and window length affect both performance and deployment requirements. A model tested on manually cleaned recordings may not remain accurate on continuous ambulatory data.

The arrhythmia multicenter study uses end-to-end multi-scale convolution and sequence learning to capture complementary temporal resolutions [43]. The Parkinson’s EEG transformer preserves electrode topology before modeling channel interactions [10]. The Parkinson’s voice framework uses explicit acoustic features and subject-level nested validation instead of end-to-end representation learning [11]. These three designs show that no single representation strategy is uniformly superior across signal types and cohort sizes.

Recording device shift is a central problem. The voice domain generalization study in [45] reduced the ability to identify the recording center while retaining cognitive classification. This type of adversarial check should be extended to ECG devices, EEG amplifiers, sensor placement, and sampling rate.

## 12. EHR and Clinical Tabular Data

EHR and clinical tabular studies are summarized in Table 6. The largest risk in this modality is temporal and process leakage.

EHR missingness is informative because clinicians order tests selectively. Treating missing values as random can erase useful workflow information, while exploiting them can make the model dependent on one institution’s practice. Both the biological variables and missingness indicators should be evaluated under external shift.

The CAD multimodal study explicitly tested performance with incomplete modalities [4]. The pulmonary embolism model used routine physical and laboratory variables, and performed comparison with established clinical scores [15]. The pediatric sepsis model used nine routine variables and a prospective seven-hospital external cohort [34]. These designs are closer to executable workflows than models based on convenience datasets with no defined index time.

Calibration is especially important for tabular risk prediction, where outputs are often interpreted as probabilities. The COVID-19 external validation study reports Brier score as well as AUC [18], while the liver fibrosis study reports confidence intervals across multiple external cohorts [14]; such reporting should become standard.

## 13. Omics and Molecular Early Detection

Omics studies are summarized in Table 7. Their promise lies in detecting molecular change before visible anatomy, while their principal shortcoming is dimensionality that far exceeds the number of independent patients.

Feature selection must be fitted inside each training fold. Selecting genes, proteins, or metabolites on the full dataset prior to cross-validation leaks outcome information into every fold. Batch correction must also avoid using test labels or future samples.

The preterm birth transformer provides a controlled comparison of cfDNA, cfRNA, and their integration [16]. The thyroid model integrates clinical variables with gene expression information, reporting 94.6% accuracy [234]. The tyrosinemia study uses a small longitudinal biomarker dataset to identify a potential early HCC signal [19]. These studies occupy different evidence levels, and should not be compared numerically.

Molecular models also require assay-level validation. A representation learned from one sequencing platform or laboratory may absorb batch identity; therefore, external validation should cross both patient population and laboratory process when possible.

## 14. Wearable and Continuous Monitoring

Wearable and continuously sampled data change early detection from a single diagnostic encounter into a longitudinal inference problem [235]. The studies in Table 8 were included only when a body-worn or continuously operating sensor contributed directly to the prediction.

### 14.1. Cardiovascular and Metabolic Monitoring

A prospective multicenter evaluation enrolled 117 patients with paroxysmal atrial fibrillation and compared continuous wrist photoplethysmography with a 14-day ECG reference monitor [235]. The convolutional model was assessed on 91,857 fifteen-minute intervals from 111 evaluable participants and achieved 96.1% sensitivity and 98.1% specificity [235]. Photoplethysmography-derived atrial fibrillation burden correlated strongly with the reference burden, with $R^{2}=0.986$ and a mean difference of 0.8% [235]. This design is stronger than validation on isolated public waveforms because it tests prolonged ambulatory acquisition, actual wear time, and burden estimation against a simultaneous reference device [235]. Nevertheless, its clinical scope remains monitoring of patients already known to have paroxysmal atrial fibrillation rather than population screening for previously undiagnosed disease [235].

A later prospective study used intermittent watch ECG segments as anchors for correcting continuous watch photoplethysmography in 1054 patients undergoing atrial fibrillation ablation [246]. The corrected CNN–LSTM system reported 98.60% sensitivity and 99.27% specificity against patch ECG [246]. Correction reduced the mean absolute percentage error of burden estimation from 1.11% to 0.85%, while the correlation with the reference remained 0.9988 [246]. The result supports complementary sensing: photoplethysmography supplies coverage, whereas intermittent ECG supplies a higher-specificity physiological anchor [246]. Because the cohort consisted of patients undergoing ablation, the evidence primarily supports post-diagnostic rhythm management and should not be interpreted as proof of screening performance in an asymptomatic population [246].

Wearable ECG can also be used to infer a laboratory abnormality rather than to classify rhythm [236]. Kardio-Net was trained on 293,557 ECG–potassium pairs from 152,508 patients, fine-tuned using 4337 ECGs from 1463 patients with end-stage renal disease, and tested at an external health system [236]. For single-lead ECG, the external AUC for severe hyperkalemia was 0.807 and the mean absolute error was 0.740 mEq/L [236]. A prospective international cohort of 40 patients produced a smartwatch AUC of 0.831 and a mean absolute error of 0.580 mEq/L [236]. The external and prospective evaluations make the study clinically informative, but the wide confidence interval around the smartwatch AUC reflects the small prospective sample [236]. This model should be viewed as a possible trigger for confirmatory blood testing rather than a replacement for serum measurement [236].

During Ramadan fasting, a free-living study combined continuous glucose monitoring with wrist-derived behavioral and physiological signals from 33 adults with type-1 diabetes [244]. Patient-wise splits were used on 1164 retained participant-days, and hypoglycemia represented approximately 4% of hourly observations [244]. The best multimodal model achieved an ROC AUC of 0.867, a precision–recall AUC of 0.341, and 77% recall for events one hour ahead [244]. At that sensitivity-focused operating point, precision was only 0.14, showing that apparently strong ROC discrimination can coexist with a substantial alert burden when events are rare [244]. The reported Brier score of approximately 0.03 supports probability calibration in the held-out data, but external and prospective interventional validation remain necessary [244].

### 14.2. Respiratory and Infectious-Disease Warning

An early COPD exacerbation system prospectively followed 67 patients for a mean of four months using a Fitbit device, home air quality sensing, and symptom reporting [242]. Twenty-five exacerbation episodes were observed, and the seven-day prediction model reported 92.1% accuracy, 94% sensitivity, 90.4% specificity, and an AUC above 0.9 [242]. Daily steps, stairs climbed, and distance moved were the most influential variables, suggesting that reduced activity may provide an early behavioral signal [242]. The event count is small relative to the dense longitudinal stream, so the effective evidence base is 25 clinical episodes rather than the much larger number of daily observations [242]. This study demonstrates feasibility, but does not establish transport across devices, climates, or healthcare systems [242].

A complementary COPD study analyzed 707 week-long armband recordings from 423 patients in the COSYCONET cohort to estimate forced expiratory volume in one second [247]. Regularized and feature-selected models achieved a mean absolute error of approximately 15%, with accelerometry variability, age, and walking measures among the most informative predictors [247]. The larger cohort strengthens estimation of the association between daily activity and lung function, but predicted FEV_1_ is not equivalent to detecting an acute exacerbation [247]. Clinical use would require a prospective study showing that changes in the wearable estimate precede deterioration and improve the timing of spirometry or treatment [247].

The WE SENSE controlled trial monitored 55 healthy adults before and after exposure to a live attenuated influenza vaccine using a smart ring, watch, and shirt [239]. Gradient-boosting models were trained on 35 participants, with validation and testing on ten participants each [239]. A night-time Oura Ring model achieved an ROC AUC of 0.73 for real-time prediction of inflammatory surges, and achieved 0.89 when a 24-h tolerance window was allowed [239]. Adding shirt-derived day and night signals increased the 24-h tolerance AUC to 0.91, whereas the symptom-only model achieved 0.79 [239]. The controlled challenge supports presymptomatic physiological detection; however, vaccine-induced inflammation is a surrogate challenge rather than naturally acquired infection, and the participant-level test set was small [239].

A prospective dengue study collected 1781 h of wearable photoplethysmography from 132 adults and 15 children admitted in Vietnam [237]. The best CNN achieved precision of 0.785 and recall of 0.771 for three-level adult severity classification [237]. In the smaller pediatric analysis, precision and recall were both 0.891 for distinguishing severe illness from post-disease follow-up [237]. The study shows that low-cost continuous sensing can encode severity information in an endemic clinical setting, but five-fold cross-validation at one hospital does not establish geographic transport [237]. The pediatric estimate is especially uncertain because it is based on only fifteen patients and compares illness with a later recovery state that may be easier to separate than concurrent severity grades [237].

### 14.3. Neurological and Functional Detection

A fixed-and-frozen Apple Watch algorithm was evaluated prospectively across four epilepsy monitoring units and an ambulatory group using accelerometry and photoplethysmography [238]. It detected all nineteen tonic–clonic seizures observed in the monitoring units and all ten ambulatory seizures, although the corresponding confidence intervals were wide [238]. Precision decreased from 68% in monitoring units to 22% in ambulatory use, while false alarms increased from 0.05 to 0.13 per 24 h [238]. The contrast illustrates why sensitivity measured in a controlled unit does not capture the alert burden created by uncontrolled daily movement [238]. The ambulatory result is also dominated by only three participants who experienced seizures, limiting conclusions about between-patient robustness [238].

A nocturnal armband study monitored 68 patients for as long as three months and accumulated 6,304 h across 788 nights [245]. Threshold pre-screening retained 94.0% of seizures while reducing the signal volume by 81%, after which an attention-based CNN–LSTM classified candidate events [245]. The final model reported mean sensitivity of 0.762, positive predictive value of 0.334, and 0.165 false alarms per hour [245]. The two-stage design is computationally practical, but the positive predictive value shows that most generated alerts were not seizures [245]. A complete home alert system should report alarms per night and caregiver workload alongside window-level accuracy [245].

Focal impaired awareness seizures are more difficult because their autonomic manifestations vary across patients [248]. A three-center study recruited 236 patients and evaluated wearable ECG against expert-labeled video-EEG events using leave-one-patient-out validation [248]. After technical exclusions and automatic data quality screening, analysis retained 161 seizures from 38 patients and produced a median sensitivity of 66.6%, with 5.2 false alarms per 24 h [248]. An exploratory subgroup of twenty responders reached median sensitivity of 100%, but selecting responders after observing performance cannot be treated as validation of a prospective personalization rule [248]. The reduction from the recruited cohort to the quality-eligible analytic cohort is itself a deployment result, as unreliable signals can make the algorithm unavailable precisely when monitoring is needed [248].

Ultra-long monitoring provides a different view of seizure risk [241]. A hybrid forecasting system used wrist heart rate and step count from eleven participants monitored for an average of 337 days, with seizures referenced by EEG [241]. The long-horizon system reduced time classified as high risk by 29% while increasing sensitivity by 11% relative to the comparison models [241]. Although long follow-up captures within-person cycles that short admissions cannot observe, eleven participants cannot establish general performance across differing epilepsy phenotypes [241]. Thus, the evidence supports individualized forecasting feasibility more strongly than a population-level model [241].

WATCH-PD collected consumer-grade measurements from 82 early-stage Parkinson’s patients and 50 age-matched comparison participants in a one-year multicenter longitudinal study [17]. Its random forest model reported 92.3% accuracy, 90.0% sensitivity, 100% specificity, and an AUC of 0.92 [17]. Longitudinal multicenter acquisition improves ecological relevance, but the modest number of controls makes perfect specificity sensitive to sample composition and threshold selection [17]. The evidence is promising for digital phenotyping, although broader screening claims require independent prospective cohorts [17].

A long-term-care study used a single accelerometer to obtain a five-meter gait recording and approximately one week of daily activity from 51 older adults [240]. Extreme gradient boosting achieved 86.3% accuracy and an AUC of 0.92 under leave-one-out cross-validation [240]. Explainability analysis associated frailty with greater stride length variability, sample entropy, and gait asymmetry [240]. The model addresses an underrepresented high-risk population, but the cross-sectional secondary analysis does not show that the identified patterns predict future transition into frailty [240]. Leave-one-out validation also reuses nearly the entire small cohort for each fitted model, making independent facility-level validation important [240].

### 14.4. Specialized Sensors and Edge Feasibility

A neck-worn accelerometer patch captured carotid flow vibrations and was evaluated in a flow phantom and 74 clinical participants [36]. Interpretable wavelet features classified at least 50% stenosis or another carotid abnormality with an AUROC of 0.97 and an AUPR of 0.947 [36]. At the prespecified threshold, sensitivity was 81.7% and specificity was 93.6% [36]. The combination of a mechanistically targeted sensor and a small feature set is attractive for screening, but independent-site validation was not reported [36]. The mixed pathology definition also requires disease-specific analysis before the device can be used to guide referral for stenosis [36].

Continuous inference must fit within the energy and latency limits of the device [243]. SleepLiteCNN classified four sleep stages from single-lead ECG with 89% accuracy and an F1 score of 89% [243]. Eight-bit quantization reduced energy to 5.48 J per inference while retaining 90% accuracy and F1 score [243]. This result demonstrates that model compression can improve deployability without an obvious loss in the reported discrimination metrics [243]. Sleep staging is not itself equivalent to diagnosing sleep apnea or insomnia, so clinical detection claims require evaluation against disease-specific endpoints rather than sleep stage labels alone [243].

### 14.5. Cross-Cutting Evidence Requirements

Wearable data create many correlated windows from comparatively few independent participants, as illustrated by the 91,857 atrial fibrillation intervals originating from 111 participants [235]. This makes participant-wise separation essential, as random window splitting can place the same person’s physiology in training and test sets [244]. Evaluation should additionally separate recording periods or devices when the intended use includes future hardware or firmware versions [235].

Missingness is clinically informative, but can also become a shortcut [248]. The focal seizure study shows that automated quality assessment can materially change sensitivity and reliability, demonstrating that signal availability is part of model performance rather than a preprocessing footnote [248]. Reports should quantify excluded time, non-wear time, charging gaps, and the fraction of patients for whom a prediction can actually be produced [248].

Alert burden must be evaluated at the timescale experienced by the patient or caregiver [238]. Despite a mean accuracy of 0.793, the nocturnal seizure system produced 0.165 false alarms per hour, illustrating how window-level accuracy can obscure operational burden [245]. The hypoglycemia study similarly achieved an ROC AUC of 0.867, while precision at the sensitivity-focused threshold was 0.14 [244]. Therefore, clinically meaningful reporting should combine event sensitivity, lead time, false alarms per day, positive predictive value, calibration, and the proportion of monitored time yielding usable signals [244].

The strongest translation evidence in this modality comes from prospective acquisition with an independent physiological or clinical reference [235]. The atrial fibrillation watch study used simultaneous continuous ECG as its comparator [235]. The potassium study added external-site testing and a prospective smartwatch cohort, but that prospective cohort included only 40 patients [236]. The next step is not merely a larger retrospective window count; prospective testing of whether wearable-triggered action improves time to confirmation, workload, treatment, or patient outcome is also needed [236].

Wearable deployment requires battery, adherence, privacy, and alert burden analyses as well. A model that produces accurate retrospective windows may still fail if patients do not wear the device during informative periods or if repeated false alerts cause disengagement.

## 15. Multimodal and Multi-Omics Learning

Table 9 contains studies where the principal contribution is the integration of more than one modality. Image feature fusion within a single modality is not classified as multimodal.

Multimodal evaluation should include unimodal baselines trained on the same patients, otherwise an apparent fusion gain may arise because the complete-case multimodal cohort differs from the unimodal cohort. Evaluation of missing modalities should distinguish random removal from clinically structured absence.

The indeterminate pulmonary nodule model can operate when a modality is unavailable, and uses three external sites [33]. The CAD model reports tolerance to as much as 50% missing data [4]. LiteCrossFusion combines MRI and clinical variables with only 1.3 million parameters [26]. These designs address complementary parts of the deployment problem, namely, external transport, incomplete data, and computational efficiency.

The pancreatic 3D-FuseNet model integrates CT, MRI, and clinical information for early-stage detection [256]. Its usefulness depends on whether every patient receives both imaging modalities at the proposed decision point. If MRI is obtained only after suspicious CT, the model may represent a second-stage diagnostic tool rather than initial screening.

Multi-omics integration differs from multimodal clinical fusion because molecular blocks may share batch effects and biological pathways. The cfDNA–cfRNA transformer demonstrates a within-study gain under a common test set [16]. External assay and population validation remain necessary before the gain can be considered generalizable.

## 16. Explainability, Calibration, Bias, and Robustness

### 16.1. Explainability Is Not Validation

Saliency maps, Grad-CAM, SHAP, LIME, attention maps, and prototype visualizations can help inspect model behavior; however, they do not prove that the highlighted region or feature caused the prediction, nor do they establish that the model will generalize. Explanation quality must be evaluated separately from discrimination.

STCWTNet applies attention-driven PageRank to identify channel relationships in EEG [10], while Deep-LIBRA uses an explicit intermediate representation of dense tissue rather than a fully opaque risk score [1] and the liver fibrosis study applies SHAP and LIME to externally validated tabular predictions [14]. These approaches all differ in what they explain, and should not be collapsed into a binary “XAI present” field.

Clinical explanations must also be stable. If small input perturbations change the highlighted region while the prediction remains fixed, then the explanation is unreliable. Agreement with expected anatomy is reassuring, but can be produced by broad heat maps even when the decision depends on a shortcut.

### 16.2. Calibration and Selective Prediction

Calibration determines whether a nominal risk corresponds to observed frequency. Because prevalence and spectrum shift can alter probability estimates, it should be assessed for the intended population. Calibration-in-the-large, calibration slope, Brier score, expected calibration error, and reliability diagrams can provide complementary information.

The Alzheimer ensemble study shows that the decision strategy changes both balanced accuracy and calibration error [12]. The COVID-19 external validation study identifies the SVM as both the strongest discriminator and the best-calibrated evaluated model [18]. The retinal self-supervised system adds an uncertainty-based referral rule instead of forcing a prediction for every image [13].

The diabetic retinopathy deep kernel study analyzes uncertainty specifically for grading and referral [257], while the skin cancer study optimizes predictive entropy to improve uncertainty accuracy and expected calibration error [41]. The heart disease hybrid model in [258] combines conformal prediction with SHAP and LIME. These techniques require evaluation of coverage, subgroup behavior, and threshold stability before they can support clinical abstention.

### 16.3. Demographic and Technical Bias

Bias can enter through disease prevalence, access to testing, acquisition devices, label quality, exclusion criteria, and missing data. Aggregate performance can remain high even when sensitivity is poor for an underrepresented group. Subgroup evaluation should report uncertainty, because small groups produce unstable estimates.

The diabetic foot ulcer study explicitly examines skin tone fairness across CNN architectures [259]. Its strongest model reported 97% accuracy, while MobileNetV2 offered lower computational demand [259]. The reported results emphasize architecture-level performance more than quantitative between-tone disparities, so a fairness claim requires detailed subgroup results.

The Parkinson’s voice analysis identifies different acoustic predictors for male and female participants [11]. The diabetic retinopathy self-supervised model reports a fairness audit stratified by image quality rather than demographic identity [13]. These analyses address different bias axes, and should not be treated as interchangeable.

The thyroid XGBoost study emphasizes the origin and quality of clinical variables while integrating gene expression [234]. Data quality vigilance is important, but algorithmic bias management must also quantify performance across clinically relevant groups. A title or methodological label alone is not evidence that unfairness was reduced.

### 16.4. Robustness and Domain Shift

Robustness to synthetic noise tests a narrow perturbation model. Clinical domain shift includes new scanners, hospitals, populations, prevalence, diagnostic criteria, and workflows. A model can be robust to pixel noise while failing under a different referral population.

Voice-domain generalization reduces center-identifying information while preserving cognitive classification [45]. MammoDG targets multi-center mammographic variation [21]. The multi-dataset polyp detector tests an unseen database [38]. These studies provide more relevant evidence than augmentation-only robustness, although prospective drift remains unmeasured.

## 17. Validation and Generalization

### 17.1. A Hierarchy of Evidence

This survey interprets validation using seven levels: cross-validation only, internal held-out testing, temporal testing, independent external validation at one or more centers, prospective silent evaluation, prospective clinical evaluation, and monitored deployment. Multicenter sampling is treated as a property of external or prospective validation rather than as a separate ordinal level, because a multicenter dataset is not necessarily independent of model development. The hierarchy describes evidence breadth rather than guaranteeing study quality. A poorly designed prospective study can remain biased, while a rigorous external retrospective study can provide valuable transport evidence.

Cross-validation estimates internal variability when all preprocessing and tuning are nested correctly. Nested cross-validation is particularly important for small cohorts because feature selection and hyperparameter tuning otherwise contaminate the evaluation. A separate test set is meaningful only if it remains untouched until the final model is fixed.

External validation should use patients outside the development source. An independent dataset created by the same archive or research program may test time or cohort shift but not full institutional transport. The CAD study uses an external dataset after a fixed internal 7:1.5:1.5 split [4]. The ECG CKD study uses a community hospital cohort after non-overlapping internal testing [5]. The liver fibrosis study tests both NHANES and a separate real-world cohort [14].

Multicenter prospective validation is uncommon. The pediatric abdominal sepsis study prospectively enrolled 308 patients across seven hospitals after retrospective development [34]. The periodontitis system used clinically labeled and radiographically labeled cohorts across four international centers, and compared performance with that of specialists [260]. These designs move closer to real use, although neither proves improved patient outcomes by itself.

### 17.2. From Retrospective Validation to Clinical Evaluation

External retrospective validation and prospective clinical evaluation test different failure mechanisms. External validation asks whether a locked model transports to new patients, institutions, time periods, or acquisition systems. Prospective silent evaluation additionally tests whether required inputs arrive reliably, whether exclusions and missingness change in real time and whether latency or alert volume makes operation impractical. An interventional study then asks whether showing the output changes clinician behavior, timeliness, safety, resource use, or patient outcomes.

DECIDE-AI provides reporting guidance for early-stage clinical evaluation of AI decision support systems, including the clinical setting, users, workflow, human–AI interaction, and observed safety issues [261]. This framework prevents a prospective label from being interpreted as evidence of benefit when the system merely runs alongside care without influencing a decision. For early detection systems, the decisive transition is not from internal to larger testing alone but from technical validity to observable performance within the intended pathway.

### 17.3. Leakage Audits

Leakage should be audited at the patient, visit, image, segment, site, and time levels. Duplicate images and augmented variants must remain in one split. Feature selection, normalization, imputation, harmonization, and resampling must be fitted only on training data. Pseudolabels generated using information from the full dataset can also leak evaluation structure.

The Parkinson’s drawing study enforced subject-wise cross-validation to avoid leakage [25], while the Parkinson’s voice study used nested subject-level folds and training-only resampling [11]. In the arrhythmia study, the authors recognized the optimism created by intra-patient paradigms [43]. These practices should be reported for every biosignal study rather than simply being inferred from the use of cross-validation.

The magnitude of leakage-induced optimism is illustrated by a lung-CT protocol using 110 patients from the IQ-OTH/NCCD dataset: patient-level fivefold cross-validation yielded accuracy results of 81.3%–83.1% with AUCs above 0.87, whereas slice-level splitting produced higher apparent performance [262]. This example is methodologically important because neighboring slices from one patient share anatomy, acquisition conditions, and disease characteristics; therefore, a model that encounters one patient’s slices during training is not being tested on a genuinely unseen patient.

### 17.4. Reporting Quality, Risk of Bias, and Applicability

Reporting quality, risk of bias, and clinical applicability should not be collapsed into one judgment. TRIPOD+AI supports transparent description of model development and evaluation so that readers can reconstruct what was done [31]. PROBAST+AI evaluates whether design and analysis choices are likely to produce biased estimates and whether the model addresses the intended clinical use [32]. A completely reported study can still have an inappropriate control group, participant overlap, inadequate event counts, or optimistic tuning; thus, a poorly reported study may be impossible to assess even when its underlying design is sound.

A systematic review of guidelines for the use of AI in medicine found a fragmented standards landscape spanning development, reporting, evaluation, ethics, and implementation [263]. The practical implication for this survey is that no single checklist exists that can substitute for clinical target definition, leakage control, external validation, calibration, subgroup analysis, and workflow evaluation. The maturity of evidence is strongest when all of these elements agree.

### 17.5. Clinician Comparison and Added Value

Comparing a model with clinicians is meaningful only when both evaluate the same cases under comparable information and time constraints. A model tested on curated images should not be compared with clinicians working in routine practice. Reader studies should also assess whether clinicians improve with assistance, not only whether the model exceeds an unaided average.

The periodontitis study reports AUROC of 94.2% for the model and 85.6% for specialists on its benchmark [260]. It further reports that junior dentists reached specialist-level performance with AI support [260]. The polyp benchmark establishes accuracy, sensitivity, and specificity for 146 gastroenterologists before a clinical trial [35]. These studies provide more actionable human comparison evidence than separate model and clinician results drawn from different case sets.

**Figure 6:**
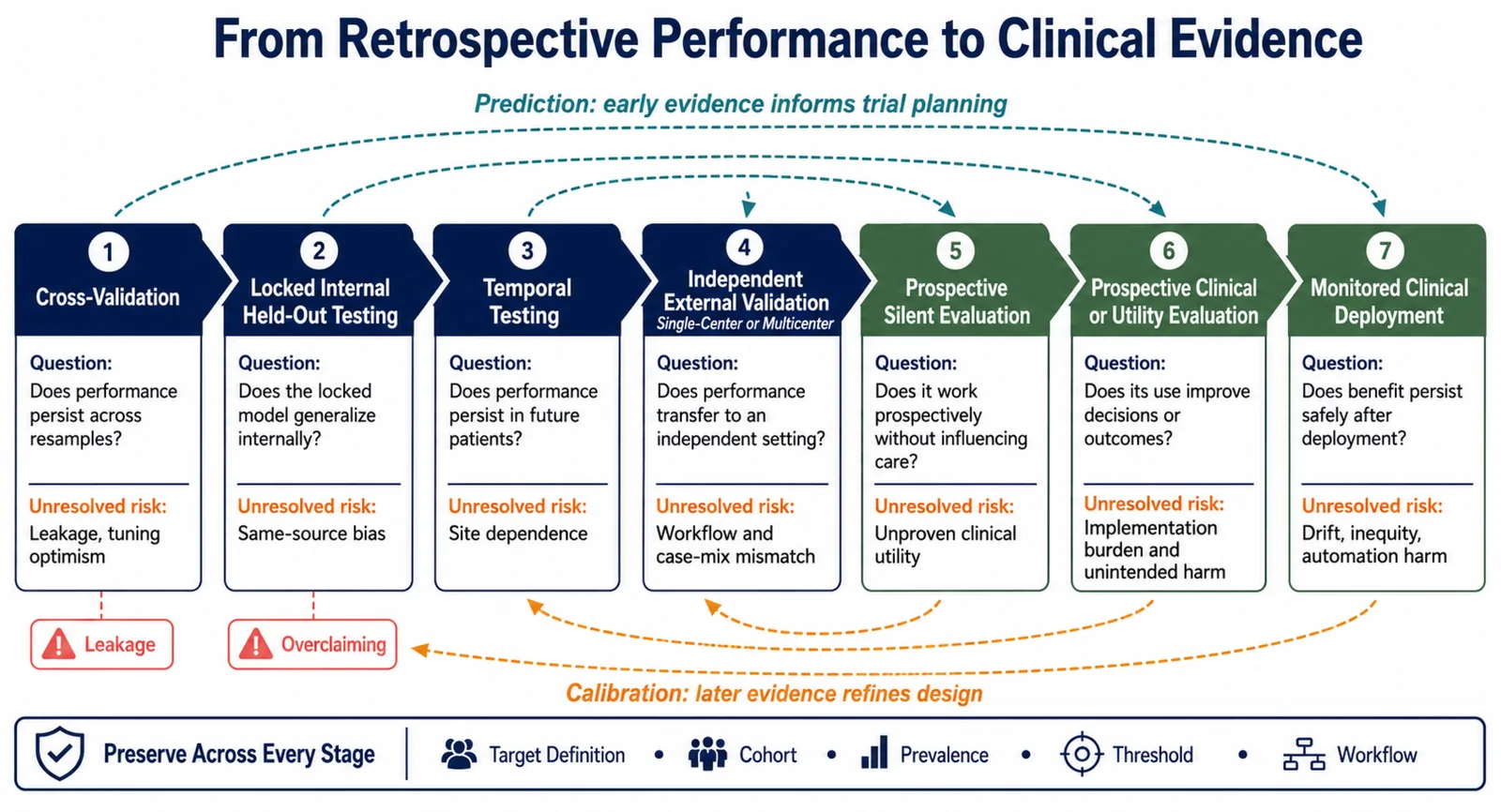
Validation maturity for early detection systems. Higher levels address broader sources of variation, but do not repair weaknesses in target definition or cohort construction.

Figure 6 distinguishes increasing validation breadth from study quality; higher levels expose additional sources of variation, but do not correct for an inappropriate target, biased cohort, or contaminated split.

## 18. Clinical Translation and Deployment

Clinical translation begins by defining who will receive the model, when it will run, what information is available, what action follows, and how errors are managed. A technically accurate system may still have no usable place in the workflow; conversely, a moderate model can be valuable if it safely prioritizes scarce confirmatory testing.

The indeterminate pulmonary nodule model addresses a defined decision involving invasive diagnostic work-up [33]. The ECG coronary calcification model addresses targeted CT screening and possible statin reclassification [42]. The arthritis thermography prototype addresses low-cost triage rather than definitive differentiation [50]. These examples connect output to action more clearly than generic statements about clinical decision support.

Prospective silent evaluation should run the locked model without showing outputs to clinicians. This stage measures data availability, missingness, drift, latency, and alert frequency without changing care. A subsequent interventional evaluation can test whether displaying the model improves decisions, timeliness, safety, or patient outcomes.

### From Model Output to a Testable Clinical Pathway

Clinical usefulness depends on the action attached to an output, not on discrimination alone; a screening threshold can determine number of people referred, disease cases missed, confirmatory tests consumed, and harms created by false alarms. These consequences vary with prevalence and capacity, so a threshold selected on a development cohort may not remain appropriate after deployment. Decision curve or utility analyses can clarify whether a model improves net benefit across plausible thresholds, but must use consequences that reflect the intended pathway.

Early-stage clinical evaluation should document who sees the output, how uncertainty is communicated, whether users can override the recommendation, and what occurs after disagreement or system failure [261]. This human–AI interaction is part of the intervention; the same probability can function as an automated rule, a second reader, a triage score, or a prompt for additional testing. Accordingly, performance studies should pre-define the workflow role and measure downstream behavior rather than assuming that a more accurate score will automatically improve care.

The gastrointestinal clinical validation review identifies a persistent gap between high controlled-study accuracy and patient-centered benefit [264]. It calls for multicenter randomized trials, generalizable algorithms, and effective human–AI collaboration [264]. This distinction is crucial because a rise in detection may also increase unnecessary biopsies, procedures, anxiety, or treatment.

Computational requirements also affect translation. A large ensemble may be feasible in a centralized imaging service but not on a wearable or low-resource clinic device. The retinal self-supervised system reports approximately 45 ms per ensemble inference on a consumer GPU and approximately 180 ms for one backbone pass on a mobile-class CPU [13]. LiteCrossFusion reports a 1.3 million parameter multimodal design [26]. These measurements are useful only when the complete preprocessing, data transfer, and referral latency are included.

Privacy must be considered during development and deployment; for example, the adversarial COVID-19 framework explicitly protects demographic attributes while retaining reported external performance [47]. Privacy-preserving representation does not remove governance requirements for access, audit, retention, or secondary use. Federated learning similarly limits centralization, but can introduce site imbalance, communication constraints, and privacy leakage through updates.

Post-deployment monitoring should track calibration, sensitivity at the operational threshold, referral volume, missingness, subgroup performance, and changes in acquisition or clinical practice. A rollback procedure is necessary when performance or data quality falls outside predefined limits. Continuous retraining should not occur without controlled validation, as an adaptive model can change its behavior after approval.

**Figure 7:**
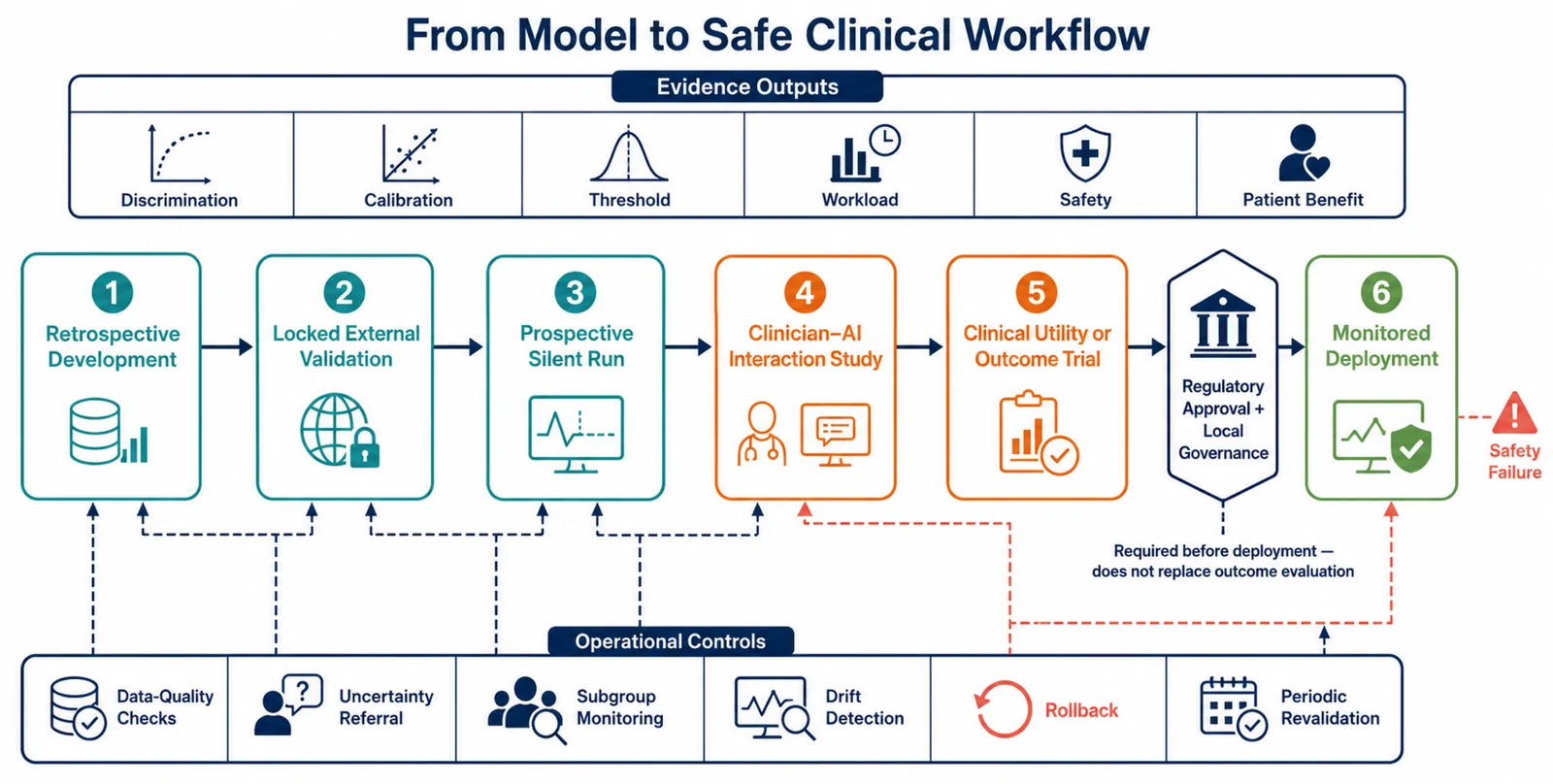
Clinical translation pathway separating model validation from workflow and outcome evidence.

Figure 7 complements the validation hierarchy by showing the later transition from a technically evaluated model to workflow integration, clinical action, and outcome assessment.

## 19. Open Challenges and Research Directions

### 19.1. Clinically Faithful Targets

Future work should replace convenient case–control labels with targets that correspond to screening, pre-diagnostic detection, or a defined risk horizon. Controls should represent the clinical differential rather than unusually healthy volunteers. Intermediate disease states should be included when they determine the real decision boundary.

### 19.2. Transport Rather than Benchmark Saturation

Repeated optimization on public datasets produces small benchmark gains while leaving site shift unresolved. Multi-site development should preserve at least one unseen center for evaluation. Domain generalization methods should report whether site information remains recoverable from the learned representation, as demonstrated in voice-based cognitive impairment detection [45].

### 19.3. Uncertainty Connected to Capacity

Uncertainty research should report accuracy and error among retained cases as a function of referral coverage. Lung, vertebral, retinal, breast, and skin studies demonstrate growing technical interest in uncertainty [39]. The next step is to evaluate whether referral rules fit specialist capacity and reduce harmful errors at the intended prevalence.

### 19.4. Fairness Beyond Aggregate Accuracy

Studies should pre-define clinically relevant subgroups and report confidence intervals rather than performing exploratory subgroup comparisons after observing results. Fairness interventions must preserve overall safety and should be tested under external demographic shift. Skin tone, sex, age, acquisition quality, and healthcare access can each create different failure mechanisms.

### 19.5. Missing Modalities and Tiered Testing

Multimodal systems should support tiered pathways in which inexpensive data trigger more costly acquisition. The pancreatic cancer review proposes EHR or biomarker triage followed by CT, MRI, or endoscopic ultrasound verification [8]. Such a pathway can be evaluated for net benefit and resource use, whereas a complete-case fusion model may not be deployable for first-line screening.

### 19.6. Reproducibility and Reporting

Every study should report patient counts, sample counts, event counts, split unit, preprocessing boundaries, hyperparameter selection, class distribution, role of the test set, missing data handling, thresholds, and confidence intervals. TRIPOD+AI supplies a structured reporting framework for prediction-model studies using regression or machine learning methods [31]. Code and model release improve computational reproducibility, but do not compensate for inaccessible cohorts, ambiguous endpoint construction, or undocumented exclusions. Reproducibility should include the data generation process and analysis decisions, not just executable model code.

**Figure 8:**
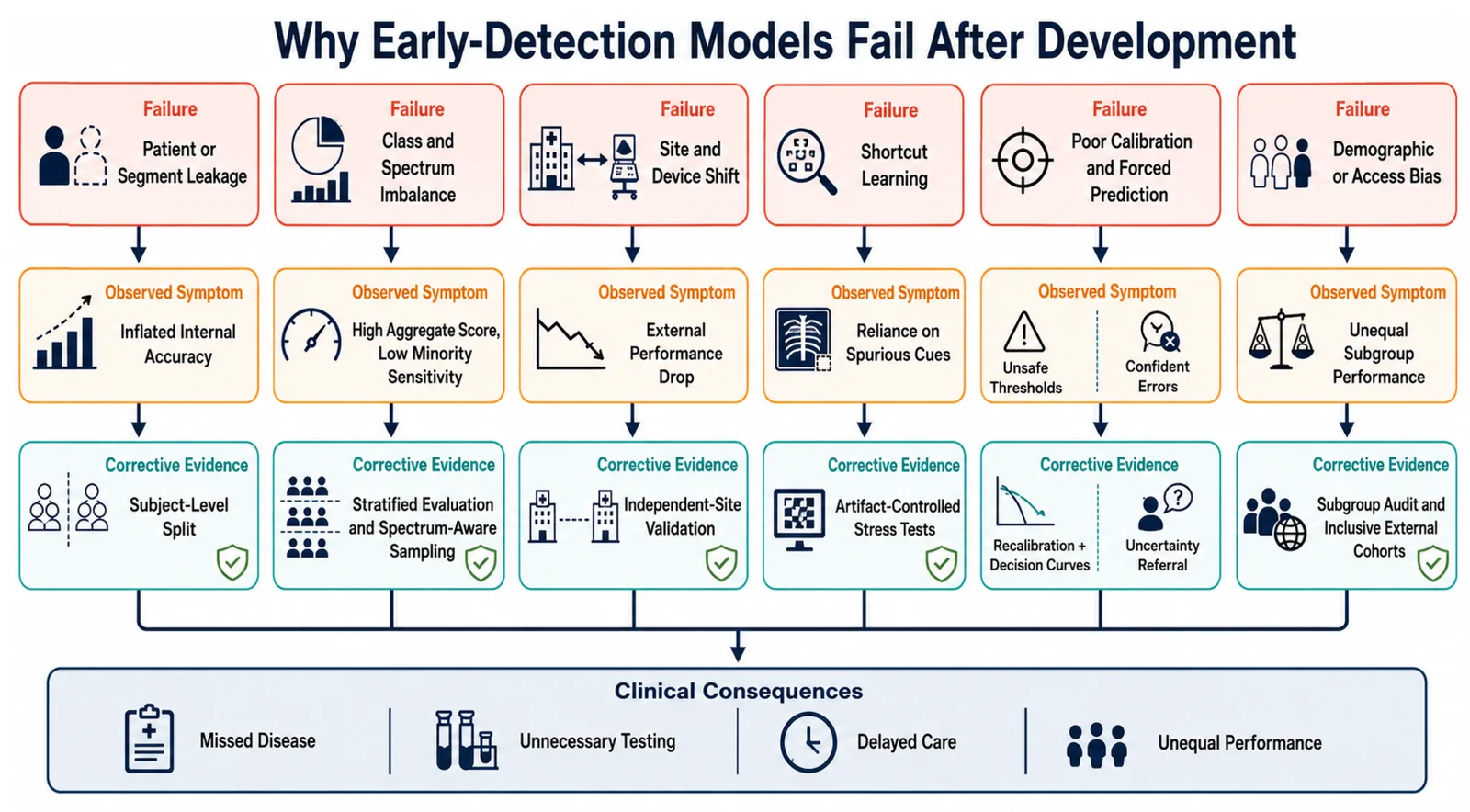
Failure mechanisms, observable symptoms, and the evidence needed to detect or mitigate them.

Figure 8 links recurrent failure mechanisms to their observable symptoms and the evidence needed for detection or mitigation.

## 20. Conclusions

ML and DL can support early disease detection when the clinical target, modality, representation, validation design, and downstream action are aligned. DL offers its clearest advantage for spatial, temporal, and cross-modal representation learning, while traditional ML remains strong for moderate-sized cohorts with informative clinical features. Reported performance cannot be interpreted independently of patient-level separation, cohort spectrum, external shift, calibration, uncertainty, and threshold consequences.

The evidence reviewed here shows that most studies remain retrospective and internally evaluated, while a smaller group provides external, multicenter, prospective, or workflow-oriented evidence. Therefore, high accuracy on a public dataset is not equivalent to clinical readiness; progress requires fewer claims based on benchmark saturation and more locked external testing, prospective silent evaluation, patient-centered utility trials, and monitored deployment. The central question is no longer whether an algorithm can classify disease-related data but whether it can provide reliable information at the right time, for the right population, and with a safer consequence than the pathway it is intended to improve upon.

## Figures and Tables

**Table 1:** Operational separation of disease-related ML and DL targets used in this survey.

Target	Decision Point	Typical Output	Primary Interpretive Requirement
Early detection	Before conventional recognition or at a documented early stage	Current probability of early disease or early pathological change	The positive class must represent an early state rather than established advanced disease.
Screening	Before definitive diagnostic work-up in an eligible population	Referral, repeat testing, or no referral	Sensitivity, specificity, prevalence-dependent predictive values, and downstream testing burden must be considered.
Diagnosis	During evaluation of suspected disease	Current diagnostic class	The sampled case–control spectrum must resemble the intended clinical differential.
Risk prediction	Before the future event or disease state	Probability over a stated time horizon	Temporal ordering, censoring, calibration, and clinically meaningful thresholds must be explicit.
Prognosis	After diagnosis	Outcome, survival, recurrence, or treatment response	The result should not be presented as evidence of earlier disease detection.
Progression prediction	After a precursor or disease state is established	Transition to a later stage	Baseline state, prediction horizon, and longitudinal independence must be defined.

**Table 2:** Cross-study synthesis of major method families; the comparison provides a summary of recurring evidentiary patterns.

Method Family	Frequent Role	Recurring Strength	Recurring Limitation	Evidence-Consistent Interpretation
Traditional ML	Tabular risk and engineered biosignal features	Effective with modest cohorts and interpretable variables	Sensitive to feature selection leakage and site-specific missingness	A necessary baseline; greater architectural complexity must demonstrate added value on the same split
CNNs	Image, waveform, and segmentation tasks	Efficient learning of local spatial or temporal structure	May exploit acquisition artifacts and repeated public dataset signatures	Strong internal accuracy does not establish cross-site robustness
Attention and transformers	Long-range, multichannel, and multimodal relationships	Flexible modeling of distant interactions	Greater data, tuning, and regularization demands	Benefits are credible mainly in matched within-study comparisons
Conventional augmentation	Small training cohorts	Simple and controllable label-preserving variation	Can become implausible or leak correlated samples if applied before splitting	Useful when transformations preserve clinical meaning and remain training-only
GANs and VAEs	Synthetic augmentation and latent representation learning	Potentially expands appearance or representation space	Memorization, artifacts, mode collapse, leakage, and uncertain diversity	Synthetic samples are not equivalent to new independent patients
Multimodal fusion	Complementary images, signals, records, or omics	Can combine non-redundant evidence	Complete-case selection, missing modalities, and alignment errors	Fusion gain must be measured against single-modality branches in the same cohort

**Table 3:** Evidence context for selected anchor studies. The table illustrates how reported performance must be interpreted together with sample size, validation design, and the clinical task.

Study and Task	Dataset Size	Validation Design	Reported Performance	Interpretive Boundary
[1] Breast density risk representation	15,661 development images from 4437 women; 6368 test mammograms from 414 cases and 1178 controls	Independent case–control evaluation	Adjusted case–control AUC 0.612, 95% CI 0.584–0.640; reader correlation 0.90	Risk discrimination and density estimation, not direct tumor detection
[33] Indeterminate pulmonary nodules	1284 development patients; three external cohorts of 155, 136, and 96 patients	Three external-site evaluations	External AUCs 0.712–0.918	Site variation remains visible; pooled performance would conceal transport differences
[5] ECG-based CKD detection	72,618 ECGs from 49,632 development patients; 16,955 internal and 10,476 external patients	Nonoverlapping internal patients and an external community hospital	AUC 0.885 internally and 0.861 externally	Retrospective risk stratification does not establish benefit from model-guided screening
[34] Pediatric abdominal sepsis	6566 retrospective development patients; 308 prospective patients	Prospective external evaluation across seven hospitals	External AUC 0.928, 95% CI 0.895–0.961; accuracy 0.873; precision 0.924	Strong transport evidence, but no interventional outcome trial
[35] Colorectal polyp benchmark	65 unedited images evaluated by 146 gastroenterologists	Controlled human benchmark before a clinical trial	Expert accuracy 77.74%, sensitivity 86.47%, specificity 74.33%	Human comparison on images does not replace live procedure evaluation
[36] Neck-worn carotid screening	Flow phantom and 74 clinical participants	Clinical cohort evaluation without an independent-site test	AUROC 0.97; AUPR 0.947; sensitivity 81.7%; specificity 93.6%; precision 85.4%; F1-score 83.5%; accuracy 89.8%	Mixed pathology endpoint requires disease-specific and external validation

**Table 4:** Representative medical imaging studies. Reported performance values are retained only as study-specific results, and are not directly ranked across datasets.

Study	Disease/Target Group	Method	Dataset	Reported Performance
[51]	Early Breast Cancer	CNN	CBIS-DDSM	Accuracy 99.17%
[52]	Early Breast Cancer	DenseNet	BreakHis	Accuracy 94.808%
[53]	Early Breast Cancer	AlexNet–ResNet–MobileNetV2 ensemble	BUSI	Accuracy 96.92% (abnormality)/94.62% (malignancy)
[54]	Early Breast Cancer	Xception	BreakHis	Accuracy 83.07%
[55]	Early Breast Cancer	Modified Xception TL	ICIAR 2018	Accuracy 99.0%; precision 99.003%; recall 98.995%; F1-score 99.0%; sensitivity 98.55%; specificity 99.14%
[56]	Early Breast Cancer	CNN	INbreast	Accuracy 96.53%
[57]	Early Breast Cancer	GRU	Kaggle	Accuracy 86.21%; precision 85.50%; sensitivity 85.60%; specificity 84.71%; F1-score 88%; AUC 0.89
[58]	Early Breast Cancer	DRDA-Net	BreaKHis	Accuracy 95.72–98.10% across magnifications
[59]	Early Breast Cancer	ResNet	MIAS	Accuracy 89.5%
[60]	Early Breast Cancer	EfficientNet	DDSM (cropped subset)	Accuracy 95.48%
[61]	Early Lung Cancer	Transformer	ACDC-LungHP	Detection accuracy 99.62%; mean IoU 49.81; validation accuracy 99.02%; validation mean IoU 49.52
[62]	Early Lung Cancer	Ensemble	LC25000	Lung-cancer accuracy 99.05%
[63]	Early Lung Cancer	SqueezeNodule-Net V1 (2D)	LUNA16	Accuracy 93.2%; specificity 94.6%; sensitivity 89.2%
[64]	Chronic Obstructive Pulmonary Disease	Graph convolutional network	Danish Lung Cancer Screening Trial	Accuracy 77%; AUC 0.81
[65]	Early Lung Cancer	ViT	LUNA16	Accuracy 98.03%
[66]	Early Lung Cancer	EfficientNet	LC25000	Test accuracy 99.72%
[67]	Early Lung Cancer	CNN-LSTM	IQ-OTH/NCCD	Accuracy 98.45%; precision 98.21%; recall 98.60%; F1-score 98.40%
[68]	Early Lung Cancer	ViT	IQ-OTH/NCCD	Accuracy 98.43%
[69]	Early Lung Cancer	CNN	IQ-OTH/NCCD	Accuracy 96%; precision 97%; sensitivity 96%; F1-score 96%; MCC 0.9406
[70]	Early Lung Cancer	DenseNet	LUNA16	Accuracy 98.7%; sensitivity 99.2%; F1-score 95.38%
[71]	Early Lung Cancer	Lung EffGhost V-Net	LIDC-IDRI; LC25000; IQ-OTH/NCCD	Accuracy 99.6%; DSC 97.87%; IoU 92.05%
[72]	Early Skin Cancer	SkinNet-14	HAM10000	Accuracy 97.85%
[73]	Early Skin Cancer	ResNet	ISIC 2018	Accuracy 91%
[74]	Early Skin Cancer	ViT	HAM10000	Accuracy 92.79%; sensitivity 91.09%; specificity 93.54%
[75]	Early Skin Cancer	Ensemble	HAM10000	Mean accuracy 99.53%
[76]	Early Skin Cancer	MobileNet	HAM10000	Accuracy 88.71%
[77]	Early Skin Cancer	EfficientNet	ISIC 2020	Accuracy 90.54%
[78]	Early Skin Cancer	Hybrid ANN	HAM10000	Top-1 accuracy 86%; top-2 accuracy 95%; AUC 96%
[79]	Early Skin Cancer	MobileNet	ISIC	Accuracy 88.39%
[80]	Early Skin Cancer	YOLOv5s–YOLOv8s fusion	ISIC (nine classes)	mAP@0.5 94.3%; precision 97.87%
[81]	Early Skin Cancer	CNN + MRFO	HAM10000	Accuracy 97.70%; loss 0.0626
[82]	Early Skin Cancer	Hybrid Capsule CNN	Nail Disease Detection	Validation accuracy 99.25%; precision 97.35%; recall 96.79%
[83]	Early Skin Cancer	Attention	ISIC 2019	Accuracy 97.4%
[84]	Early Skin Cancer	ViT	HAM10000	Accuracy 96.74%; precision 95.46%; recall 96.27%; specificity 96.00%; F1-score 95.86%
[85]	Early Brain Tumor	MobileNet	Kaggle	Accuracy 97%
[86]	Early Brain Tumor	Random Forest	Kaggle	Accuracy 99%
[87]	Early Brain Tumor	ML	BRaTs 2020	Classification accuracy 98%; multiclass segmentation Dice 92%
[88]	Early Brain Tumor	YOLOv5	BRATS 2018 subset	Accuracy 85.95%
[89]	Early Brain Tumor	CNN	Kaggle	Accuracy 97.8%; specificity 98.5%; recall 96.2%; F1-score 98.5%; precision 97.3%
[90]	Early Brain Tumor	CNN	Kaggle	Accuracy 97%
[91]	Early Brain Tumor	SVM	Kaggle	Accuracy 62%; sensitivity 82%
[92]	Early Brain Tumor	ResNet	Kaggle	Accuracy 90.2%
[93]	Early Brain Tumor	VGG	T1-weighted CE	Accuracy 95%
[94]	Early Brain Tumor	ResNet101	Kaggle	Accuracy 99.62%; precision 96%; recall 98%; F1-score 97%
[95]	Early Brain Tumor	Autoencoder + GBM	Brain Cancer Detection MRI	Accuracy 96.8%; precision 97.4%; recall 96.2%; F1-score 96.8%; AUC 0.99
[96]	Additional Cancer Types	CNN–DBN + PSOBER	Kaggle oral images	Accuracy 97.35%
[97]	Additional Cancer Types	GhostNet	SIPakMeD	Accuracy 96.39%; precision 96.42%; recall 96.39%; specificity 99.09%
[24]	Additional Cancer Types	Autoencoder	Herlev	Accuracy 99.2%
[98]	Additional Cancer Types	Six-classifier comparison	STRAMPN ovarian histopathology	Accuracy 99%
[99]	Additional Cancer Types	BiLSTM	Kaggle	Accuracy 98%; recall 99.7%; precision 93%; F1-score 98%
[100]	Additional Cancer Types	Attention	Kaggle	Accuracy 94.3%
[101]	Additional Cancer Types	CerviSegNet-DistillPlus	Cx22	Accuracy 94%; sensitivity 96.23%; specificity 92.19%; Dice 0.9855
[102]	Additional Cancer Types	Majority-voting ensemble	PICCOLO	Classification accuracy 94.25%; segmentation DSC 92.44%
[103]	Additional Cancer Types	EfficientNet	C-NMC	Accuracy, recall, precision, specificity, and F1-score: 97.34% each
[104]	Additional Cancer Types	CNN	PBS image	Initial accuracy 97.85%; augmented accuracy 99.32%; precision 99.35%; recall 99.33%; F1-score 99.58%
[105]	Additional Cancer Types	EfficientNet + NSGA-II	Colon histopathology images	Accuracy 99.97%
[106]	Additional Cancer Types	MobileNet	Sipakmed	Accuracy 97.9%
[107]	Additional Cancer Types	GoogLeNet	SUN colonoscopy	Accuracy 98%
[108]	Additional Cancer Types	CNN	KVASIR	Accuracy 98%
[109]	Additional Cancer Types	CNN	TCGA	Accuracy increased from 75% to 91%; reported AUC-ROC 95%
[110]	Diabetes and Diabetic Eye Disease	Attention	DFUC	Accuracy 98%; F1-score 90%
[111]	Diabetes and Diabetic Eye Disease	Dual-branch DL	APTOS 2019	Binary accuracy 98.50%; sensitivity 99.46%; specificity 97.51%; stage-grading accuracy 89.60%; sensitivity 89.60%; specificity 97.72%; quadratic weighted kappa 93.00%
[112]	Diabetes and Diabetic Eye Disease	3D-CNN + feature fusion	Six retinal datasets	Mean accuracy 97.71%
[113]	Diabetes and Diabetic Eye Disease	CNN	DIARETDB1	Accuracy 92%
[114]	Diabetes and Diabetic Eye Disease	CNN ensemble	Diabetic Retinopathy	Validation accuracy 87.31%
[115]	Diabetes and Diabetic Eye Disease	ResNet	EyePACS	Accuracy 86.36%
[116]	Diabetes and Diabetic Eye Disease	Inception	APTOS	Accuracy 86.54%
[117]	Diabetes and Diabetic Eye Disease	ResNet	Kaggle	Accuracy 91%
[118]	Diabetes and Diabetic Eye Disease	Ensemble of ensembles	Combined ocular-disease images	Accuracy 88%
[119]	Diabetes and Diabetic Eye Disease	ResNet	ODIR	Accuracy 98%; precision 98%
[120]	Diabetes and Diabetic Eye Disease	InceptionResNet V2	MURED	Accuracy 49.85%
[121]	Diabetes and Diabetic Eye Disease	EfficientNet	Kaggle	Accuracy 95.12%; precision 95.21%; recall 94.88%; F1-score 95.00%; Dice 94.91%; Jaccard 91.2%; MCC 0.925
[122]	Diabetes and Diabetic Eye Disease	ResNet	Mendeley OCT	Accuracy 99.5%
[123]	Diabetes and Diabetic Eye Disease	LSTM	DRISHTI-GS	Accuracy 97.4%; sensitivity 97%; specificity 97.9%
[124]	Diabetes and Diabetic Eye Disease	DenseNet	Messidor	Accuracy 88%
[125]	Neurological Disorder	VGG19	NIATS	Accuracy 96.67%
[126]	Early Dementia	Supervised ML	ADNI	Accuracy 92.5%
[127]	Neurological Disorder	ResNet	Kaggle	Test accuracy 97.31%
[128]	Alzheimer Disease	VGG	ADNI	Binary accuracies: 93.05% (AD/MCI), 86.39% (MCI/CN), and 92.00% (AD/CN); ternary accuracy 93.12%
[129]	Neurological Disorder	EfficientNet	ADNI	Accuracy 98.94% (CN vs. AD); 98.42% (MCI vs. CN)
[130]	Neurological Disorder	ViT	Kaggle	Accuracy 99.60%; precision 99.0%; recall 98.1%; F1-score 98.51%
[131]	Neurological Disorder	CNN	Kaggle	Accuracy 99.68%
[132]	Neurological Disorder	VGG	Kaggle	Accuracy 94%
[133]	Neurological Disorder	Multiple transfer-learning models	Kaggle AD MRI	maximum Accuracy 99.96%
[134]	Neurological Disorder	XGBoost	Kaggle	Accuracy 87%
[135]	Neurological Disorder	DenseNet	Kaggle	Accuracy 89.38%
[136]	Neurological Disorder	CNN with attention	ADNI	Accuracy 96.64%
[137]	Neurological Disorder	CNN	ADNI	Accuracy 81.80%; recall 82.50%; precision 81.80%; specificity 80.50%; validation AUC 91.5%
[138]	Neurological Disorder	DenseNet264	ADNI	sMCI vs. AD: training accuracy 82.5%, training AUC 87.63%, test accuracy 81.03%; AD vs. CN: training accuracy and AUC 100%, test accuracy 99.56%
[139]	Respiratory and Infectious Disease	DenseNet	COVIDx	Accuracy 99.44%; COVID-19 precision 98.40%; sensitivity 99.59%; specificity 99.50%
[140]	Respiratory and Infectious Disease	Ensemble	Montgomery	Accuracy 98.4%
[141]	Respiratory and Infectious Disease	EfficientNet	Kaggle	Accuracy 92%
[142]	Respiratory and Infectious Disease	kNN	Kaggle	Accuracy 98.07%
[143]	Respiratory and Infectious Disease	Multi-stage SqueezeNet/ResNet-50/EfficientNet-b0	COVID-QU-Ex	Stage accuracies: 99% (normal vs. abnormal), 98% (COVID-19 vs. non-COVID), and 97% (pneumonia/TB subclassification)
[144]	Respiratory and Infectious Disease	Xception	Kaggle + center CT data	Accuracy 90.34%; precision, recall, and F1-score 90.91% each; AUC 0.9668
[145]	Respiratory and Infectious Disease	DenseNet on COVID-19	5902 chest X-ray images	Accuracy 98.2%; sensitivity 97.6%; specificity 98.4%
[146]	Respiratory and Infectious Disease	OSegNet	QaTa-COV19	Accuracy 99.65%; precision 98.09%
[147]	Respiratory and Infectious Disease	VGG	Kaggle	Accuracy 86%
[148]	Kidney and Liver Disease	ViT	CKD	Classification accuracy 94.38%; segmentation IoU 82.34%; Dice 91.57%
[149]	Early-Stage Lung Cancer	Attention	LIDC-IDRI	Accuracy 94.2%; AUC 0.957
[150]	Autism Spectrum Disorder	InceptionV3 + CNN	ABIDE I	Accuracy 85.71%; precision 100%
[151]	Autism Spectrum Disorder	Multiple DL architectures	ABIDE MRI	Accuracy 80–84%
[152]	Cancer Type and Subtype	VMobileNet–Vception fusion	Eight cancer classes/26 subclasses	Accuracy 99.95/99.13% (major/subclass)
[153]	Autism Spectrum Disorder	CNN	ABIDE	Accuracy 99.39%; recall 98.80%; precision 99.85%; F1-score 99.32%
[154]	Autism Spectrum Disorder	YOLO	ABIDE	Accuracy 99.3%; precision 98.9%; recall 99.1%; F1-score 99.0%

**Table 5:** Representative biosignals studies. Reported performance values are retained only as study-specific results, and are not directly ranked across datasets.

Study	Disease/Target Group	Method	Dataset	Reported Performance
[160]	Cardiovascular Disease	HybDeepNet	MIT-BIH	Accuracy 98.6%
[161]	Heart Failure	ML	MIMIC	Accuracy 98%; sensitivity 97.60%; specificity 96.90%; precision 97.20%
[162]	Cardiovascular Disease	CNN	PTB	Training/validation/test accuracies 99.37%/99.15%/99.31%; top F1-score 0.99
[163]	Cardiovascular Disease	ML	Physionet Chal	Accuracy 98.03%; sensitivity 97.64%; specificity 98.43%
[164]	Cardiovascular Disease	BiLSTM	Shaoxing	Accuracy 98.57% (lead II); 98.34% (lead aVF)
[165]	Cardiovascular Disease	CNN	MIT-BIH	Binary accuracy 98.35%; multiclass accuracy 99.3%
[166]	Cardiovascular Disease	EfficientNet	MIT-BIH	Accuracy 98.78% and 99.22% on the two evaluated datasets
[167]	Cardiovascular Disease	Attention	MIT-BIH	Accuracy 99.18%; F1-score 99.18%
[168]	Cardiovascular Disease	CNN	MIT-BIH	Training/validation/test accuracies 99.50%/99.06%/99.34%; top F1-score 0.99
[169]	Cardiovascular Disease	LSTM	MIT-BIH	Accuracy 97%
[170]	Neurological Disorder	XGBoost	DementiaBank	Accuracy 75.59%
[171]	Neurological Disorder	SpectroCVT-Net	BrainLat	Accuracy 92.59% ± 2.3%
[172]	Neurological Disorder	Attention	IBIB-PAN	Accuracy 99.9%
[173]	Neurological Disorder	CNN	UCI	Accuracy 98.4%
[174]	Neurological Disorder	BiLSTM	CHB-MIT	Accuracy 95.03%
[175]	Neurological Disorder	Two-branch DL framework	CHB-MIT	Patient-specific mean accuracy 96.36%; F1-score 96.38%
[176]	Neurological Disorder	Lightweight CNN ensemble	MUMTAZ benchmark	Accuracy LSSOCV 94.1%
[177]	Neurological Disorder	ResNet	MODMA	Accuracy 95.63%; F1-score 95.97%; sensitivity 95.06%; specificity 96.31%
[178]	Neurological Disorder	SVM	Bonn EEG	Accuracy 100%
[179]	Respiratory and Infectious Disease	ViT	ICBHI	Accuracy 99.23%
[180]	Depression	ECAPA-TDNN	Chinese depression	Accuracy 90.4%; mean F1-score 90.8%

**Table 6:** Representative EHR and clinical tabular studies. Reported performance values are retained only as study-specific results, and are not directly ranked across datasets.

Study	Disease/Target Group	Method	Dataset	Reported Performance
[181]	Early Breast Cancer	SVM+PSO	Coimbra	Accuracy 91%
[182]	Early Breast Cancer	Optimized ensemble + XAI	Wisconsin	Accuracy 99.99%
[183]	Early Breast Cancer	Shallow ANN	WBCD	Accuracy 99.85%; specificity 99.72%; sensitivity 100%; precision 99.69%; F1-score 99.84%; AUC 99.86%
[184]	Early Breast Cancer	Logistic Reg.	Kaggle	Accuracy 99.65%
[185]	Early Lung Cancer	BiLSTM	MIMIC	Accuracy 98.1%; MCC 96.2%
[186]	Early Lung Cancer	XGBoost	Kaggle	Accuracy 98%; precision 98%; sensitivity 98%
[187]	Additional Cancer Types	Random Tree	Kaggle	Accuracy 98.33%
[188]	Cardiovascular Disease	Random Forest	Kaggle	Accuracy 99.12%
[189]	Cardiovascular Disease	Extra Trees	Kaggle	Accuracy 98.15%
[190]	Cardiovascular Disease	XGBoost	UCI	Accuracy 92.2%
[191]	Cardiovascular Disease	Ensemble	UCI	Accuracy 86.89%
[192]	Cardiovascular Disease	XGBoost	CVD	Accuracy 95.24%
[193]	Cardiovascular Disease	Random Forest	Cleveland	Accuracy 82.6%; precision 81.5%; recall 83.7%; F1-score 82.5%
[194]	Cardiovascular Disease	Gradient Boosting	Taiwanese adult cohort	Accuracy 76.2%; AUC 0.846; sensitivity 77.6%; specificity 75.9%
[195]	Cardiovascular Disease	Boosting Ensemble	Cleveland	Accuracy 93.44%
[196]	Cardiovascular Disease	Enhanced MLP	CDC cardiac disease	Accuracy 92%
[197]	Cardiovascular Disease	CardioSenseNet/ SADNet	Cleveland and CVD	Accuracy 99%
[198]	Cardiovascular Disease	XGBoost	Kaggle	Accuracy 94.34%; F1-score 95.19%
[199]	Diabetes and Diabetic Eye Disease	Neural Network	UCI	Accuracy 88.6%
[200]	Diabetes and Diabetic Eye Disease	Autoencoder	PIMA	Accuracy 92.31%
[201]	Diabetes and Diabetic Eye Disease	LSTM	Pima	Accuracy 99%
[202]	Diabetes and Diabetic Eye Disease	Logistic Reg.	PIMA	Accuracy 77.6%; sensitivity 72.4%; specificity 79.6%; Type I error 27.6%; Type II error 20.4%
[203]	Diabetes and Diabetic Eye Disease	XGBoost	Pima	Accuracy 96.84%; AUC 0.99
[204]	Diabetes and Diabetic Eye Disease	Autoencoder	PIMA	Accuracy 92.16%; F1-score 90.29%; ROC AUC 94%
[205]	Diabetes and Diabetic Eye Disease	Multilayer perceptron	PIMA Indian Diabetes dataset	Accuracy 86.08%
[206]	Diabetes and Diabetic Eye Disease	Extra Trees	PIMA	Accuracy 96.58%
[207]	Diabetes and Diabetic Eye Disease	Random Forest	Sylhet	Accuracy 97.5%; F1-score 97.5%; ROC AUC 99.80%
[208]	Diabetes and Diabetic Eye Disease	Gradient Boosting	PIMA	Accuracy 90.25%; ROC score 96.38%; F1-score 86.48%
[209]	Diabetes and Diabetic Eye Disease	XGBoost	Iraqi diabetes	Accuracy 99.50%; precision 99.52%; recall 99.50%; F1-score 99.51%
[210]	Diabetes and Diabetic Eye Disease	Proposed DL model	UCI diabetes	Accuracy 98.71%; precision 98.74%; recall 98.71%; F1-score 98.71%
[211]	Neurological Disorder	SVM	PPMI	Accuracy 86.3%
[212]	Neurological Disorder	MLP	OASIS	Accuracy 95.75%
[213]	Kidney and Liver Disease	Random Forest	CKD	Accuracy 96.8%; recall 1.00 for CKD cases
[214]	Kidney and Liver Disease	Deep ensemble	CKD longitudinal data	Accuracy 99.3/99.2% (6/12 months)
[215]	Kidney and Liver Disease	Robust ML model	UCI CKD	Accuracy 100%
[216]	Kidney and Liver Disease	Genetic Programming	UCI	Accuracy 99.33%; AUC 0.99
[217]	Kidney and Liver Disease	TrioNet	CKD	Accuracy 98.97%
[218]	Kidney and Liver Disease	LSTM	MIMIC	Accuracy 92.4%
[219]	Kidney and Liver Disease	Improved DBN	CKD	Accuracy 98%
[220]	Kidney and Liver Disease	Dense-layer DNN	CKD	Accuracy, precision, recall, and F1-score 99%; ROC AUC 0.9821; MCC 0.9727
[221]	Kidney and Liver Disease	BWOA feature selection	CKD	Accuracy 100%
[222]	Kidney and Liver Disease	CatBoost+HHO	ILPD	Accuracy 85%
[223]	Kidney and Liver Disease	Decision Tree	CKD	Accuracy 98.60%
[224]	Osteoporosis Risk	XGBoost	NHANES	Accuracy 93%; AUC 0.94; mean cross-validation score 0.929 ± 0.030
[225]	Depression	Ensemble	NHANES	Accuracy 95.4%; F1-score 0.976
[226]	Autism Spectrum Disorder	ML	UCI	Accuracy 99.77%
[227]	Autism Spectrum Disorder	Gradient Boosting	Clinical ASD	Accuracy 98.18%; sensitivity 98.16%; precision 98.16%
[228]	Autism Spectrum Disorder	LSTM	ABIDE	Accuracy 95%
[229]	Autism Spectrum Disorder	XGB/SVM + ET selection	ASD screening data	Accuracy, precision, recall, and F1-score 100%

**Table 7:** Representative omics and molecular studies. Reported performance values are retained only as study-specific results, and are not directly ranked across datasets.

Study	Disease/Target Group	Method	Dataset	Reported Performance
[230]	Additional Cancer Types	Random Forest	NCI PBSII	Accuracy 99.2%
[231]	Spinal Muscular Atrophy	Autoencoder + FCN	SMA transcriptomic data	Cross-validation accuracy 90.0%; ROC AUC 0.948; false discovery rate 5.0%
[232]	Genetic Disease	XGBoost	Kaggle	Accuracy 98%
[233]	Renal Cancer Stage	Weighted GCN	TCGA-KIRC	Accuracy 82%

**Table 8:** Representative wearable and continuous monitoring studies. Reported performance values are retained only as study-specific results, and are not directly ranked across datasets.

Study	Disease/Target Group	Method	Dataset	Reported Performance
[235]	Atrial Fibrillation	PPG-based CNN	111 evaluable participants; 91,857 intervals	Sensitivity 96.1%; specificity 98.1%
[236]	Severe Hyperkalemia in ESRD	Kardio-Net on smartwatch ECG	40-patient prospective smartwatch cohort	AUC 0.831; MAE 0.580 mEq/L
[237]	Dengue Severity	CNN on wearable PPG	132 adults and 15 children; 1781 h	Adult precision 0.785; recall 0.771; paediatric precision and recall 0.891
[238]	Tonic–Clonic Seizures	Fixed accelerometer–PPG algorithm	Four monitoring units plus ambulatory cohort	Monitoring units: sensitivity 100%, FAR 0.05/24 h, precision 68%, latency 32.07 s; ambulatory: sensitivity 100%, FAR 0.13/24 h, precision 22%, latency 37.38 s
[239]	Presymptomatic Inflammatory Response	Gradient boosting on ring/watch/shirt signals	55 healthy adults in controlled trial	Ring ROC AUC: 0.73 real-time, 0.89 at 24 h; shirt 24-h AUC 0.91, specificity 0.83, F1-score 0.65
[240]	Frailty	XGBoost on gait and activity	51 long-term-care residents	Accuracy 86.3%; AUC 0.92
[241]	Epileptic Seizure Risk	Hybrid ML and cycle model	11 participants; mean 337 days	High-risk time reduced 29%; sensitivity increased 11%
[242]	Seven-Day COPD Exacerbation	ML/DNN with wearable and environment data	67 patients; 25 exacerbations	Accuracy 92.1%; sensitivity 94%; specificity 90.4%
[243]	Four-Stage Sleep Classification	Quantized SleepLiteCNN	Single-lead ECG	Accuracy and F1 90%; 5.48 J/inference
[244]	One-Hour Hypoglycemia Risk	Multimodal BiLSTM	33 adults; 1164 participant-days	ROC AUC 0.867; PR AUC 0.341; event sensitivity 77%; precision 0.14; Brier score approximately 0.03
[245]	Nocturnal Major Seizures	Attention CNN–LSTM	68 patients; 6304 h	Accuracy 0.793; sensitivity 0.762; PPV 0.334; FAR 0.165/h
[246]	Atrial Fibrillation Burden	Corrected PPG CNN–LSTM	1054 ablation patients	Sensitivity 98.60%; specificity 99.27%; burden MAPE reduced from 1.11% to 0.85%; correlation 0.9988
[36]	Carotid Pathology	Interpretable ML on neck accelerometry	Flow phantom and 74 patients	AUROC 0.97; AUPR 0.947; sensitivity 81.7%; specificity 93.6%; precision 85.4%; F1-score 83.5%; accuracy 89.8%
[247]	COPD Lung Function Estimation	Regularized interpretable ML	707 recordings from 423 patients	FEV1 MAE approximately 15%
[248]	Focal Impaired-Awareness Seizures	ECG quality assessment plus DL	161 seizures from 38 analyzable patients	Median sensitivity 66.6%; FAR 5.2/24 h

**Table 9:** Representative multimodal and multi-omics studies. Reported performance values are retained only as study-specific results, and are not directly ranked across datasets.

Study	Disease/Target Group	Method	Dataset	Reported Performance
[249]	Cardiovascular Disease	Multimodal DL	Qatar Biobank	Accuracy 78.3%
[250]	Cardiovascular Disease	ResNet	PhysioNet	Accuracy 91.6%; AUC 0.962
[251]	Autism Spectrum Disorder	Autoencoder	ABIDE	Accuracy 64.9%
[252]	Early Cancer Detection	ViT	TCGA	Accuracy 96.4%; precision 95.8%; recall 95.1%; F1-score 95.4%; AUC-ROC 0.97; inference time 19.7 ms/image
[253]	Brain Stroke	Swin	BrSCTHD-2025	Accuracy 98%; AUC 1.00
[254]	Osteoporosis	Multimodal fusion	OsteoNet	Accuracy 92.4%; AUC 0.96
[255]	Pulmonary Embolism	Attention LSTM	RadFusion	Accuracy 86%

## Data Availability

No new data were created or analyzed in this study. Data sharing is not applicable to this article.

## Abbreviations

The following abbreviations are used in this manuscript:

AD	Alzheimer’s disease
AI	Artificial intelligence
ANN	Artificial neural network
AUC/AUROC	Area under the receiver operating characteristic curve
AUPR/AUPRC	Area under the precision–recall curve
CAD	Coronary artery disease
CCTA	Coronary computed tomography angiography
CI	Confidence interval
CKD	Chronic kidney disease
CNN	Convolutional neural network
COPD	Chronic obstructive pulmonary disease
CT	Computed tomography
DBN	Deep belief network
DL	Deep learning
DNN	Deep neural network
DSC	Dice similarity coefficient
ECG	Electrocardiography
EEG	Electroencephalography
EHR	Electronic health record
F1	Harmonic mean of precision and recall
GAN	Generative adversarial network
GPU	Graphics processing unit
IoU	Intersection over union
LIME	Local interpretable model-agnostic explanations
LSTM	Long short-term memory
MAE	Mean absolute error
MCC	Matthews correlation coefficient
ML	Machine learning
MCI	Mild cognitive impairment
MLP	Multilayer perceptron
MRI	Magnetic resonance imaging
NPV	Negative predictive value
OCT	Optical coherence tomography
PD	Parkinson’s disease
PPG	Photoplethysmography
PPV	Positive predictive value
RNN	Recurrent neural network
ROC	Receiver operating characteristic
SHAP	Shapley additive explanations
SVM	Support vector machine
UQ	Uncertainty quantification
VAE	Variational autoencoder
ViT	Vision transformer
XAI	Explainable artificial intelligence

## References

[1] Haji Maghsoudi O., Gastounioti A., Scott C., Pantalone L., Wu F.-F., Cohen E.A., Winham S., Conant E.F., Vachon C., Kontos D. (2021). Deep-LIBRA: An artificial-intelligence method for robust quantification of breast density with independent validation in breast cancer risk assessment. Med. Image Anal..

[2] Francesconi A., di Biase L., Cappetta D., Rebecchi F., Soda P., Sicilia R., Guarrasi V. (2025). Class balancing diversity multimodal ensemble for Alzheimer’s disease diagnosis and early detection. Comput. Med. Imaging Graph..

[3] Messica S., Cohen S., Hadad A., Gordon M., Katz O., Presil D., Dagan N., Tsumi E., Rokach L. (2026). Temporal Integrative Machine Learning for Early Detection of Diabetic Retinopathy Using Fundus Imaging and Electronic Health Records. IEEE J. Biomed. Health Inform..

[4] Tu Y., Yang X., Liu F., Ai R., Luo Y., Ran C., Yang G., Zhang T., Jiang L. (2026). Multimodal deep learning with missing data robustness for enhanced early diagnosis of coronary artery disease using CCTA, clinical, and ECG data. BMC Med. Inform. Decis. Mak..

[5] Tsai P.-H., Lee S.-Y., Wei C.-L.H., Hsu Y.-J., Lin C. (2025). ECG-based deep learning for chronic kidney disease detection and cardiovascular risk prediction. BMC Med. Inform. Decis. Mak..

[6] Kour T., Raina J.K., Gondhi N.K.r, Sharma A., Banerjee S., Kumar P., Sharma R., Panjaliya R.K. (2026). Transformative Impact of Deep Learning and Machine Learning in Oncology: A Comprehensive Review of AI-Based Approaches for Early Detection, Diagnosis and Therapeutics Across Different Cancer Types. Arch. Comput. Methods Eng..

[7] Huang Y.i, Lu S., Dai H., Wang W., Liu B., Abudoumijiti M., Jin Q., Wu J., Chen K., Wang Y. (2026). Artificial intelligence-driven multi-cancer screening: Achievements, challenges, and future prospects. Intell. Med..

[8] Apostolou V., Papageorgiou G., Tjortjis C. (2026). Artificial intelligence for early detection of pancreatic cancer: Pre-diagnostic detection across imaging, biomarkers, and EHRs: A systematic review. Evol. Intell..

[9] Miah A.S.M., Suzuki T., Shin J. (2025). A Methodological and Structural Review of Parkinson’s Disease Detection Across Diverse Data Modalities. IEEE Access.

[10] Liu Y., Deng J., Xu R., Zhou Y. (2026). STCWTNet: Spatio-temporal channel-wise transformer network for interpretable cross-subject mild cognitive impairment detection using EEG signals. Biomed. Signal Process. Control.

[11] Briscoe D.K., Castedo R.V., Clarke P., Stella D.D., Deng J.D. (2026). Interpretable voice-based parkinson’s detection: Sex-specific acoustic biomarkers with subject-level nested cross-validation. Biomed. Signal Process. Control.

[12] Hammonds S.K., Eftestøl T., Kurz K.D., Fernandez-Quilez A. (2026). Decision Strategies in AI-Based Ensemble Models in Opportunistic Alzheimer’s Detection from Structural MRI. J. Imaging Inform. Med..

[13] Kumar N.A., Madhusudan D., Ioannou I., Pradeep Ghantasala G.S., Vassiliou V. (2026). A self-supervised hybrid CNN with uncertainty-aware referral for diabetic retinopathy screening. Biomed. Signal Process. Control.

[14] Zhu G., Yi Q., Xu R., Xie Y., Song Y., Wang X., Huang L., Xiang Y. (2026). Explainable machine learning models predict liver fibrosis risk and outcome in the general population: Development and multi-cohort external validation. Comput. Methods Programs Biomed..

[15] Zhou Y., Shen L., Alimu A., You Y., Huang C., Lu X., Li L. (2026). Machine-learning-based models assist the early prediction of pulmonary embolism: A retrospective, multicenter study. Biomed. Signal Process. Control.

[16] Zhou S., Guan C., Deng S., Zhu Y., Yang W., Zhang X., Wang X., Yang J., Zhu S., Jiang H. (2025). A novel sequence-based transformer model architecture for integrating multi-omics data in preterm birth risk prediction. npj Digit. Med..

[17] Johnson S., Kantartjis M., Severson J., Dorsey R., Adams J.L., Kangarloo T., Kostrzebski M.A., Best A., Merickel M., Amato D. (2024). Wearable Sensor-Based Assessments for Remotely Screening Early-Stage Parkinson’s Disease. Sensors.

[18] Campagner A., Carobene A., Cabitza F. (2021). External validation of Machine Learning models for COVID-19 detection based on Complete Blood Count. Health Inf. Sci. Syst..

[19] Fuenzalida K., Leal-Witt M.J., Acevedo A., Muñoz M., Gudenschwager C., Arias C., Cabello J.F., La Marca G., Rizzo C., Pietrobattista A. (2025). Integrating Machine Learning and Follow-Up Variables to Improve Early Detection of Hepatocellular Carcinoma in Tyrosinemia Type 1: A Multicenter Study. Int. J. Mol. Sci..

[20] He Z., Zhang R., Qu P., Meng Y., Jia J., Wang Z., Wang P., Ni Y., Shan L., Liao M. (2025). Development and validation of an explainable model of brain injury in premature infants: A prospective cohort study. Comput. Methods Programs Biomed..

[21] Yang Y., Wang S., Liu L., Hickman S., Gilbert F.J., Schönlieb C.-B., Aviles-Rivero A.I. (2026). MammoDG: Generalisable Deep Learning Advances Cross-Domain Multi-Center Breast Cancer Screening. Int. J. Comput. Vis..

[22] Zhang Y., Han Z., Liu L., Wang S. (2024). DualA-Net: A generalizable and adaptive network with dual-branch encoder for medical image segmentation. Comput. Methods Programs Biomed..

[23] AG K.M., Venugopal A., Kavitha M.S., SP S.I. (2025). Lightweight GANs in domain generalized medical image segmentation. Signal Image Video Process..

[24] Khamparia A., Gupta D., Rodrigues J.J.P.C., de Albuquerque V.H.C. (2021). DCAVN: Cervical cancer prediction and classification using deep convolutional and variational autoencoder network. Multimed. Tools Appl..

[25] Vallejo M., Alty J., Smith S.L. (2026). Deep learning analysis of figure copying tasks for Parkinson’s disease detection with GAN-based data augmentation. Biomed. Signal Process. Control.

[26] Dino H.I., Hassan M.M. (2026). LiteCrossFusion: Lightweight Transformer-Based Multimodal Fusion with Explainability and Uncertainty Quantification for Parkinson’s Disease Detection. Signal.

[27] Osuala R., Kushibar K., Garrucho L., Linardos A., Szafranowska Z., Klein S., Glocker B., Diaz O., Lekadir K. (2023). Data synthesis and adversarial networks: A review and meta-analysis in cancer imaging. Med. Image Anal..

[28] Waseem H.M., Islam S.U., Matragkas N., Epiphaniou G., Arvanitis T.N., Maple C. (2026). Review of generative AI for synthetic data generation: A healthcare perspective. Artif. Intell. Rev..

[29] Takahashi S., Sakaguchi Y., Kouno N., Takasawa K., Ishizu K., Akagi Y., Aoyama R., Teraya N., Bolatkan A., Shinkai N. (2024). Comparison of Vision Transformers and Convolutional Neural Networks in Medical Image Analysis: A Systematic Review. J. Med. Syst..

[30] Pena S.M., Kammer M.N., Whatley S., Welty V.F., Godfrey C.M., Paez R., Knight M., Rowe D.J., Antic S., Deppen S.A. (2026). Evaluation of Lung Cancer Probability Models and Guideline Recommendations in Settings with a High Prevalence of Cancer. CHEST Pulm..

[31] Collins G.S., Moons K.G.M., Dhiman P., Riley R.D., Beam A.L., Van Calster B., Ghassemi M., Liu X., Reitsma J.B., Van Smeden M. (2024). TRIPOD+AI statement: Updated guidance for reporting clinical prediction models that use regression or machine learning methods. BMJ.

[32] Moons K.G.M., Damen J.A.A., Kaul T., Hooft L., Andaur Navarro C., Dhiman P., Beam A.L., Van Calster B., Celi L.A., Denaxas S. (2025). PROBAST+AI: An updated quality, risk of bias, and applicability assessment tool for prediction models using regression or artificial intelligence methods. BMJ.

[33] Gao R., Li T., Tang Y., Xu K., Khan M., Kammer M., Antic S.L., Deppen S., Huo Y., Lasko T.A. (2022). Reducing uncertainty in cancer risk estimation for patients with indeterminate pulmonary nodules using an integrated deep learning model. Comput. Biol. Med..

[34] Cao S., Cai D., Zhang S., He Y., Yuan X., Zhu Z., Miao X., Wu S., Zhong Y., Yang F. (2026). Development and multicenter validation of an explainable machine learning diagnostic criteria for pediatric abdominal sepsis. npj Digit. Med..

[35] Sánchez-Peralta L.F., Glover B., Saratxaga C.L., Ortega-Morán J.F., Nazarian S., Picón A., Pagador J.B., Sánchez-Margallo F.M. (2023). Clinical Validation Benchmark Dataset and Expert Performance Baseline for Colorectal Polyp Localization Methods. J. Imaging.

[36] Majditehran H., Sang B., Desai N., Nahab F., Kvantaliani N., Blanke D., Starnes D., Christopher H., Park J.-W., Ayazi F. (2026). Automated Detection of Carotid Artery Stenosis Using a Sensitive Accelerometer Wearable Sensor and Interpretable Machine Learning. Biosensors.

[37] Salam A., Chowdhury M.H., Murugappan M., Chowdhury M.E.H. (2025). Multicentered Data Based Polyp Detection Using Colonoscopy Images Using DNN. Int. J. Imaging Syst. Technol..

[38] Mert A., Hakan Buyuklu A., Bayram B., Erpolat Tasabat S. (2025). Multi-Dataset Training Strategy for Robust Polyp Detection with Clinical Validation Insights. IEEE Access.

[39] Zahari R., Cox J., Obara B. (2024). Uncertainty-aware image classification on 3D CT lung. Comput. Biol. Med..

[40] Muduli D., Kumar R.R., Pradhan J., Kumar A. (2025). An empirical evaluation of extreme learning machine uncertainty quantification for automated breast cancer detection. Neural Comput. Appl..

[41] Shamsi A., Asgharnezhad H., Bouchani Z., Jahanian K., Saberi M., Wang X., Razzak I., Alizadehsani R., Mohammadi A., Alinejad-Rokny H. (2023). A novel uncertainty-aware deep learning technique with an application on skin cancer diagnosis. Neural Comput. Appl..

[42] Han C., You S.C., Lee H.-C., Park J.Y., Lim H.-S., Park C., Pak H.-N., Kwon O.-S., Kim S., Kim J.-S. (2026). Electrocardiogram-based deep learning score for coronary artery calcification reclassifies cardiovascular risk and identifies screening candidates. BMC Med. Inform. Decis. Mak..

[43] Ma C., Lan K., Wang J., Yang Z., Zhang Z. (2022). Arrhythmia detection based on multi-scale fusion of hybrid deep models from single lead ECG recordings: A multicenter dataset study. Biomed. Signal Process. Control.

[44] Jeong Y., Kim J., Kim D., Kim J., Lee K. (2021). Methods for improving deep learning-based cardiac auscultation accuracy: Data augmentation and data generalization. Appl. Sci..

[45] Kim M., Youn Y.C., Won Y., Choi H., Shim Y., Ryoo N., Jeong H.T., Yun G., Lee H., Kim S. (2025). Domain generalization for voice-based cognitive impairment detection. BMC Med. Inform. Decis. Mak..

[46] Del Pup F., Brun R., Iotti F., Paccagnella E., Pezzato M., Bertozzo S., Zanola A., Tshimanga L.F., Müller H., Atzori M. (2026). TransformEEG: Towards improving model generalizability in deep learning-based EEG Parkinson’s disease detection. Neurocomputing.

[47] Rohanian O., Kouchaki S., Soltan A., Yang J., Rohanian M., Yang Y., Clifton D. (2023). Privacy-Aware Early Detection of COVID-19 Through Adversarial Training. IEEE J. Biomed. Health Inform..

[48] Abdar M., Salari S., Qahremani S., Lam H.-K., Karray F., Hussain S., Khosravi A., Acharya U.R., Makarenkov V., Nahavandi S. (2023). UncertaintyFuseNet: Robust uncertainty-aware hierarchical feature fusion model with Ensemble Monte Carlo Dropout for COVID-19 detection. Inf. Fusion.

[49] Xiong H., Wang Z., Sharan R.V., Berkovsky S. (2025). Uncertainty-guided attention learning for malaria parasite detection in thick blood smears. Neural Netw..

[50] Avila-Camacho F.-J., Moreno-Villalba L.-M., Cortes-Altamirano J.-L., Alfaro-Rodríguez A., Lara-Figueroa H.-N., Herrera-López M.-E., Romero-Morelos P. (2025). Infrared Thermography and Deep Learning Prototype for Early Arthritis and Arthrosis Diagnosis: Design, Clinical Validation, and Comparative Analysis. Technologies.

[51] Raiaan M.A.K., Fahad N.M., Mukta M.S.H., Shatabda S. (2024). Mammo-Light: A lightweight convolutional neural network for diagnosing breast cancer from mammography images. Biomed. Signal Process. Control.

[52] Noaman N.F., Kanber B.M., Smadi A.A., Jiao L., Alsmadi M.K. (2024). Advancing Oncology Diagnostics: AI-Enabled Early Detection of Lung Cancer Through Hybrid Histological Image Analysis. IEEE Access.

[53] Sahu A., Das P.K., Meher S. (2024). An efficient deep learning scheme to detect breast cancer using mammogram and ultrasound breast images. Biomed. Signal Process. Control.

[54] Rana M., Bhushan M. (2023). Classifying breast cancer using transfer learning models based on histopathological images. Neural Comput. Appl..

[55] Mukhlif A.A., Al-Khateeb B., Mohammed M.A. (2023). Breast cancer images Classification using a new transfer learning technique. Iraqi J. Comput. Sci. Math..

[56] Karthiga R., Narasimhan K., Amirtharajan R. (2022). Diagnosis of breast cancer for modern mammography using artificial intelligence. Math. Comput. Simul..

[57] Wang X., Ahmad I., Javeed D., Zaidi S.A., Alotaibi F.M., Ghoneim M.E., Daradkeh Y.I., Asghar J., Eldin E.T. (2022). Intelligent Hybrid Deep Learning Model for Breast Cancer Detection. Electronics.

[58] Chattopadhyay S., Dey A., Singh P.K., Sarkar R. (2022). DRDA-Net: Dense residual dual-shuffle attention network for breast cancer classification using histopathological images. Comput. Biol. Med..

[59] Alruwaili M., Gouda W. (2022). Automated Breast Cancer Detection Models Based on Transfer Learning. Sensors.

[60] Dehghan Rouzi M., Moshiri B., Khoshnevisan M., Akhaee M.A., Jaryani F., Salehi Nasab S., Lee M. (2023). Breast Cancer Detection with an Ensemble of Deep Learning Networks Using a Consensus-Adaptive Weighting Method. J. Imaging.

[61] Talib L.F., Amin J., Sharif M., Raza M. (2024). Transformer-based semantic segmentation and CNN network for detection of histopathological lung cancer. Biomed. Signal Process. Control.

[62] Talukder M.A., Islam M.M., Uddin M.A., Akhter A., Hasan K.F., Moni M.A. (2022). Machine learning-based lung and colon cancer detection using deep feature extraction and ensemble learning. Expert Syst. Appl..

[63] Tsivgoulis M., Papastergiou T., Megalooikonomou V. (2022). An improved SqueezeNet model for the diagnosis of lung cancer in CT scans. Mach. Learn. Appl..

[64] Li Z.i, Huang K., Liu L., Zhang Z. (2022). Early detection of COPD based on graph convolutional network and small and weakly labeled data. Med. Biol. Eng. Comput..

[65] Mastouri R., Khlifa N., Neji H., Hantous-Zannad S. (2024). Performance analysis of various deep learning models based on Max-Min CNN for lung nodule classification on CT images. Mach. Vis. Appl..

[66] Akinyemi J.D., Akinola A.A., Adekunle O.O., Adetiloye T.O., Dansu E.J. (2023). Lung and Colon Cancer Detection from CT Images Using Deep Learning. Mach. Graph. Vis..

[67] Lavanya Mathiyalagi A., Ravi D.R. (2026). Revolutionizing lung cancer detection using hybrid CNN-LSTM (HCLSTM) for accurate diagnosis and enhanced nodule classification. Biomed. Signal Process. Control.

[68] Balaha H.M., Ahmed R.A., Balaha M.H. (2025). LDD-VTS: An AI-based framework for lung disease diagnosis using vision transformers and SHAP. Health Inf. Sci. Syst..

[69] Islam M.R., Sarker M.N.H., Miah M.K.-E.-S., Hossain M.H. (2025). Enhancing Lung Cancer Screening with an Automated Deep Learning System: A Resource-Efficient Approach. Int. J. Imaging Syst. Technol..

[70] Kumari J., Sinha S., Singh L. (2026). Advanced hyperparameter optimization for lung cancer detection using DenseBeetle network. Chemom. Intell. Lab. Syst..

[71] Sujatha T., Mahendhiran P.D., Daniel E. (2026). LUNG EFFGHOST V-NET: An Enhanced Novel Model for Precise Lung Cancer Segmentation and Detection. Biomed. Signal Process. Control.

[72] Al Mahmud A., Azam S., Khan I.U., Montaha S., Karim A., Haque A., Zahid Hasan M., Brady M., Biswas R., Jonkman M. (2024). SkinNet-14: A deep learning framework for accurate skin cancer classification using low-resolution dermoscopy images with optimized training time. Neural Comput. Appl..

[73] Thwin S.M., Park H.-S. (2024). Skin Lesion Classification Using a Deep Ensemble Model. Appl. Sci..

[74] Gamage L., Isuranga U., Meedeniya D., De Silva S., Yogarajah P. (2024). Melanoma Skin Cancer Identification with Explainability Utilizing Mask Guided Technique. Electronics.

[75] Chanda D., Onim M.S.H., Nyeem H., Ovi T.B., Naba S.S. (2024). DCENSnet: A new deep convolutional ensemble network for skin cancer classification. Biomed. Signal Process. Control.

[76] Abd Elaziz M., Dahou A., Mabrouk A., El-Sappagh S., Aseeri A.O. (2023). An Efficient Artificial Rabbits Optimization Based on Mutation Strategy For Skin Cancer Prediction. Comput. Biol. Med..

[77] Reis H.C., Turk V., Khoshelham K., Kaya S. (2022). InSiNet: A deep convolutional approach to skin cancer detection and segmentation. Med. Biol. Eng. Comput..

[78] Tajjour S., Garg S., Chandel S.S., Sharma D. (2023). A novel hybrid artificial neural network technique for the early skin cancer diagnosis using color space conversions of original images. Int. J. Imaging Syst. Technol..

[79] Mabrouk A., Dahou A., Elaziz M.A., Díaz Redondo R.P., Kayed M. (2022). Medical Image Classification Using Transfer Learning and Chaos Game Optimization on the Internet of Medical Things. Comput. Intell. Neurosci..

[80] Aishwarya N., Yaythish Kannaa G.S., Seemakurthy K. (2025). YOLOSkin: A fusion framework for improved skin cancer diagnosis using YOLO detectors on Nvidia Jetson Nano. Biomed. Signal Process. Control.

[81] Adamu S., Alhussian H., Aziz N., Abdulkadir S.J., Alwadin A., Abdullahi M., Garba A. (2025). Unleashing the power of Manta Rays Foraging Optimizer: A novel approach for hyper-parameter optimization in skin cancer classification. Biomed. Signal Process. Control.

[82] Shandilya G., Gupta S., Bharany S., Rehman A.U., Kaur U., Som H.M., Hussen S. (2024). Autonomous detection of nail disorders using a hybrid capsule CNN: A novel deep learning approach for early diagnosis. BMC Med. Inform. Decis. Mak..

[83] Naveed A., Naqvi S.S., Khan T.M., Razzak I. (2024). PCA: Progressive class-wise attention for skin lesions diagnosis. Eng. Appl. Artif. Intell..

[84] Ali A., Shahbaz H., Damaševičius R. (2025). xCViT: Improved Vision Transformer Network with Fusion of CNN and Xception for Skin Disease Recognition with Explainable AI. Computers.

[85] Mijwil M.M., Doshi R., Hiran K.K., Unogwu O.J., Bala I. (2023). Mobilenetv1-based deep learning model for accurate brain tumor classification. Mesopotamian J. Comput. Sci..

[86] Kumar K., Jyoti K. (2024). Machine learning for brain tumor classification: Evaluating feature extraction and algorithm efficiency. Discov. Artif. Intell..

[87] Nazir M., Ali M.J., Tufail H.Z., Shahid A.R., Raza B., Shakil S., Khurshid K. (2022). Multi-task learning architecture for brain tumor detection and segmentation in MRI images. J. Electron. Imaging.

[88] Dipu N.M., Shohan S.A., Salam K.M.A. (2021). Deep Learning Based Brain Tumor Detection and Classification. 2021 International Conference on Intelligent Technologies.

[89] Soewu T., Singh D., Rakhra M., Chakraborty G.S., Singh A. (2022). Convolutional Neural Networks for MRI-Based Brain Tumor Classification. Proceedings–2022 3rd International Conference on Computation.

[90] Pikulkaew K. (2023). Enhancing Brain Tumor Detection with Gradient-Weighted Class Activation Mapping and Deep Learning Techniques. Proceedings of the JCSSE 2023–20th International Joint Conference on Computer Science and Software Engineering.

[91] Arowolo M.O., Ajayi W.O., Olawoye P.O., Babajide O.S., Aigbogun H.E., Salawu M.D., Adebiyi M.O., Adebiyi A.A. (2024). Empowering Healthcare with AI: Brain Tumor Detection Using MRI and Multiple Algorithms. 2024 International Conference on Science, Engineering and Business for Driving Sustainable Development Goals (SEB4SDG).

[92] Najeeb R.S.M., Dahl I.O.A.M. (2022). Brain Tumor Segmentation Utilizing Generative Adversarial, Resnet and Unet Deep Learning. 2022 8th International Conference on Contemporary Information Technology and Mathematics.

[93] Hardjodipuro R.D.R., Sugiyarto M.A., Wigati F.K., Gunawan A.A.S., Setiawan K.E. (2024). Brain Cancer Classification based on T1-Weighted Images Using Deep Learning. International Conference on Artificial Intelligence and Mechatronics System.

[94] Sharma A., Guleria K., Sharma S. (2025). Optimized Brain Tumor Detection Using Transfer Learning-Based EfficientNet, ResNet101, and DenseNet121 Techniques. 2025 International Conference on Information.

[95] Kirti A., Meel P. (2025). Enhanced Brain Cancer Detection Using a Radiomics-Informed Hybrid Autoencoder and GBM Model. Proceedings of the ACCTHPA 202–Conference on 2025 Advanced Computing and Communication Technologies for High Performance Applications.

[96] Myriam H., Abdelhamid A.A., El-Kenawy E.-S.M., Ibrahim A., Eid M.M., Jamjoom M.M., Khafaga D.S. (2023). Advanced Meta-Heuristic Algorithm Based on Particle Swarm and Al-Biruni Earth Radius Optimization Methods for Oral Cancer Detection. IEEE Access.

[97] Chen W., Shen W., Gao L., Li X. (2022). Hybrid Loss-Constrained Lightweight Convolutional Neural Networks for Cervical Cell Classification. Sensors.

[98] Singh S., Maurya M.K., Singh N.P. (2024). STRAMPN: Histopathological image dataset for ovarian cancer detection incorporating AI-based methods. Multimed. Tools Appl..

[99] Mukhedkar M., Rohatgi D., Vuyyuru V.A., Ramakrishna K.V.S.S., El-Ebiary Y.A.B., Daniel V.A.A. (2023). Feline Wolf Net: A Hybrid Lion-Grey Wolf Optimization Deep Learning Model for Ovarian Cancer Detection. Int. J. Adv. Comput. Sci. Appl..

[100] Hameed U., Ur Rehman M., Rehman A., Damaševičius R., Sattar A., Saba T. (2024). A deep learning approach for liver cancer detection in CT scans. Comput. Methods Biomech. Biomed. Eng. Imaging Vis..

[101] Kang J., Li N. (2024). CerviSegNet-DistillPlus: An Efficient Knowledge Distillation Model for Enhancing Early Detection of Cervical Cancer Pathology. IEEE Access.

[102] Saad A.I., Maghraby F.A., Badawy O.M. (2024). PolyDSS: Computer-aided decision support system for multiclass polyp segmentation and classification using deep learning. Neural Comput. Appl..

[103] Abd El-Aziz A.A., Mahmood M.A., Abd El-Ghany S. (2025). A Robust EfficientNetV2-S Classifier for Predicting Acute Lymphoblastic Leukemia Based on Cross Validation. Symmetry.

[104] Thiriveedhi A., Ghanta S., Biswas S., Pradhan A.K. (2025). ALL-Net: Integrating CNN and explainable-AI for enhanced diagnosis and interpretation of acute lymphoblastic leukemia. PeerJ Comput. Sci..

[105] Saba N., Zafar A., Suleman M., Zafar K., Zafar S., Saleem A.A., Siddiqui H.U.R., Iqbal M., Ullah S.S. (2024). A Synergistic Approach to Colon Cancer Detection: Leveraging EfficientNet and NSGA-II for Enhanced Diagnostic Performance. IEEE Access.

[106] Maurya R., Rajput L., Mahapatra S. (2024). Deep and Domain Specific Feature-Based Cervical Cancer Classification Using Support Vector Machine Optimized with Particle Swarm Optimization. IEEE Access.

[107] Sai Rakshana K., Ananth A.D., Gowri L. (2025). A Novel Ensemble Deep Learning-Based Polyp Detection Using Colonoscopy Dataset. Artif. Intell. Appl..

[108] Pratik S., Sharma P., Nayak D.R., Balabantaray B.K. (2025). WMCF-Net: Wavelet pooling-based multiscale contextual fusion network for polyp classification. Biomed. Signal Process. Control.

[109] Pavithra S., Kumar L.A., Phaniraj H. (2024). Deep Learning Perspective for Preliminary Detection and Classification of Ovarian Cancer. 2024 IEEE 9th International Conference for Convergence in Technology.

[110] Khan M.A., Zaman M., Kehkashan T., Ivkovic N., Uros V., Akhunzada A. (2025). Early Detection of Diabetic Foot Ulcers through CoAtnet Model: A Scalable Solution for Healthcare Integration. ICHORA 2025–2025 7th International Congress on Human-Computer Interaction.

[111] Shakibania H., Raoufi S., Pourafkham B., Khotanlou H., Mansoorizadeh M. (2024). Dual branch deep learning network for detection and stage grading of diabetic retinopathy. Biomed. Signal Process. Control.

[112] Maqsood S., Damaševičius R., Maskeliūnas R. (2021). Hemorrhage detection based on 3d cnn deep learning framework and feature fusion for evaluating retinal abnormality in diabetic patients. Sensors.

[113] Liu T., Chen Y., Shen H., Zhou R., Zhang M., Liu T., Liu J. (2021). A Novel Diabetic Retinopathy Detection Approach Based on Deep Symmetric Convolutional Neural Network. IEEE Access.

[114] Kale Y., Sharma S. (2023). Detection of five severity levels of diabetic retinopathy using ensemble deep learning model. Multimed. Tools Appl..

[115] Herrero-Tudela M., Romero-Oraá R., Hornero R., Gutiérrez Tobal G.C., López M.I., García M. (2025). An explainable deep-learning model reveals clinical clues in diabetic retinopathy through SHAP. Biomed. Signal Process. Control.

[116] Oulhadj M., Riffi J., Khodriss C., Mahraz A.M., Bennis A., Yahyaouy A., Chraibi F., Abdellaoui M., Andaloussi I.B., Tairi H. (2023). Diabetic Retinopathy Prediction Based on Wavelet Decomposition and Modified Capsule Network. J. Digit. Imaging.

[117] Devi Y.S., Kumar S.P. (2022). A deep transfer learning approach for identification of diabetic retinopathy using data augmentation. IAES Int. J. Artif. Intell..

[118] Patil Y., Shetty A., Kale Y., Patil R., Sharma S. (2024). Multiple ocular disease detection using novel ensemble models. Multimed. Tools Appl..

[119] Shoaib M.R., Emara H.M., Mubarak A.S., Omer O.A., Abd El-Samie F.E., Esmaiel H. (2025). Revolutionizing diabetic retinopathy diagnosis through advanced deep learning techniques: Harnessing the power of GAN model with transfer learning and the DiaGAN-CNN model. Biomed. Signal Process. Control.

[120] Gualsaqui M.G., Cuenca S.M., Rosero I.L., Almeida D.A., Cadena C., Villalba F., Cruz J.D. (2023). Multi-class Classification Approach for Retinal Diseases. J. Adv. Inf. Technol..

[121] Alsohemi R., Dardouri S. (2025). Fundus Image-Based Eye Disease Detection Using EfficientNetB3 Architecture. J. Imaging.

[122] Mathews M.R., Anzar S.T.M. (2023). A lightweight deep learning model for retinal optical coherence tomography image classification. Int. J. Imaging Syst. Technol..

[123] Veena H.N., Patil K.K., Vanajakshi P., Ambore A., Gowda N.C. (2024). An Enhanced RNN-LSTM Model for Fundus Image Classification to Diagnose Glaucoma. SN Comput. Sci..

[124] Ajith Kumar S., Kumar J.S., Bharadwaj Mahabaleswara S.C. (2025). Efficient diabetic retinopathy detection using deep learning approaches and Raspberry Pi 4. Bull. Electr. Eng. Inform..

[125] Huang Y., Chaturvedi K., Nayan A.-A., Hesamian M.H., Braytee A., Prasad M. (2024). Early Parkinson’s Disease Diagnosis through Hand-Drawn Spiral and Wave Analysis Using Deep Learning Techniques. Information.

[126] Herzog N.J., Magoulas G.D. (2021). Brain asymmetry detection and machine learning classification for diagnosis of early dementia. Sensors.

[127] Asaduzzaman M., Alom M.K., Karim M.E. (2025). ALZENET: Deep learning-based early prediction of Alzheimer’s disease through magnetic resonance imaging analysis. Telemat. Inform. Rep..

[128] Kumar S.S., Nandhini M. (2021). Entropy Slicing Extraction and Transfer Learning Classification for Early Diagnosis of Alzheimer Diseases with sMRI. ACM Trans. Multimed. Comput..

[129] Şener B., Acici K., Sümer E. (2024). Categorization of Alzheimer’s disease stages using deep learning approaches with McNemar’s test. PeerJ Comput. Sci..

[130] Demirtaş Alpsalaz S., Aslan E., Özüpak Y., Alpsalaz F., Uzel H. (2025). Alzheimer’s Classification with a MaxViT-Based Deep Learning Model Using Magnetic Resonance Imaging. J. Appl. Sci. Technol. Trends.

[131] Dardouri S. (2025). An efficient method for early Alzheimer’s disease detection based on MRI images using deep convolutional neural networks. Front. Artif. Intell..

[132] Shukla A., Tiwari R., Tiwari S. (2023). Alz-ConvNets for Classification of Alzheimer Disease Using Transfer Learning Approach. SN Comput. Sci..

[133] Lokesh K., Challa N.P., Satwik A.S., Kiran J.C., Kumar Rao N., Naseeba B. (2023). Early Alzheimer’s Disease Detection Using Deep Learning. EAI Endorsed Trans. Pervasive Health Technol..

[134] Vanaja T., Shanmugavadivel K., Subramanian M., Kanimozhiselvi C.S. (2025). Advancing Alzheimer’s detection: Integrative approaches in MRI analysis with traditional and deep learning models. Neural Comput. Appl..

[135] Alwakid G.N., Tahir S., Humayun M., Gouda W. (2024). Improving Alzheimer’s Detection with Deep Learning and Image Processing Techniques. IEEE Access.

[136] Jumaili M.L.F., Sonuç E. (2025). An Attention-Based CNN Framework for Alzheimer’s Disease Staging with Multi-Technique XAI Visualization. Computers.

[137] Abbasian P., Hammond T.A. (2024). A Comprehensive Analysis of Early Alzheimer Disease Detection from 3D sMRI Images Using Deep Learning Frameworks. Information.

[138] Agarwal D., Berbis M.A., Martín-Noguerol T., Luna A., Garcia S.C.P., de la Torre-Díez I. (2022). End-to-End Deep Learning Architectures Using 3D Neuroimaging Biomarkers for Early Alzheimer’s Diagnosis. Mathematics.

[139] Xu Y., Lam H.-K., Jia G., Jiang J., Liao J., Bao X. (2023). Improving COVID-19 CT classification of CNNs by learning parameter-efficient representation. Comput. Biol. Med..

[140] Saif A.F.M., Imtiaz T., Shahnaz C., Zhu W.-P., Ahmad M.O. (2021). Exploiting cascaded ensemble of features for the detection of tuberculosis using chest radiographs. IEEE Access.

[141] Sathwik A.S., Naseeba B., Kiran J.C., Lokesh K., Deepthi Ch V.S., Challa N.P. (2023). Early Detection of Monkeypox Skin Disease Using Patch Based DL Model and Transfer Learning Techniques. EAI Endorsed Trans. Pervasive Health Technol..

[142] Gowri L., Pradeepa S., Panchada V., Amirtharajan R. (2024). Enhancing pneumonia detection with masked neural networks: A deep learning approach. Neural Comput. Appl..

[143] Deepak G.D., Bhat S.K. (2026). Detection of COVID-19 & non-COVID diseases from chest-X-rays using deep learning-based CNNs. Soft Comput..

[144] Zheng Y., Ren R., Zuo T., Chen X., Li H., Xie C., Weng M., He C., Xu M., Wang L. (2024). Prediction of early-phase cytomegalovirus pneumonia in post-stem cell transplantation using a deep learning model. Technol. Health Care Off. J. Eur. Soc. Eng. Med..

[145] Hussain K., Khan S.A., Muzaffar M., Khan Z.R., Farooq S.U. (2023). Performance evaluation of CNN architectures for COVID-19 detection from X-ray images. Comput. Methods Biomech. Biomed. Eng. Imaging Vis..

[146] Degerli A., Kiranyaz S., Chowdhury M.E.H., Gabbouj M. (2022). Osegnet: Operational segmentation network for COVID-19 detection using chest X-ray images. Proceedings–International Conference on Image Processing.

[147] Phine S. (2023). Pneumonia Classification Using Deep Learning VGG19 Model. IEEE Int. Conf. Control Autom..

[148] Islam M.S.B., Sumon M.S.I., Sarmun R., Bhuiyan E.H., Chowdhury M.E.H. (2024). Classification and segmentation of kidney MRI images for chronic kidney disease detection. Comput. Electr. Eng..

[149] Raju B.A., Veeramsetty V. (2026). Multi-Scale Spatial-Channel Attention for Early-Stage Lung CT Classification. Proceedings of the 9th International Conference on Inventive Computation Technologies.

[150] Purkayastha M., Maheswari S., Sasikumar P. (2025). Application of Transfer Learning in Autism Spectrum Disorder Detection Using Brain Imaging. Proceedings–2025 2nd International Conference on Artificial Intelligence.

[151] Mostafa S., Karim Z., Hossain M.I. (2023). Autism Detection Based on MRI Images Using Deep Learning. 2023 26th International Conference on Computer and Information Technology.

[152] Omar Bappi J., Rony M.A.T., Shariful Islam M., Alshathri S., El-Shafai W. (2024). A Novel Deep Learning Approach for Accurate Cancer Type and Subtype Identification. IEEE Access.

[153] Narayanan N., K R R., Varghese B.M. (2025). Early detection of autism spectrum disorder via deep-learning application of fMRI and machine learning for ASD children identifications. Int. J. Hybrid Intell. Syst..

[154] Gholami S., Motamed S., Askari E. (2026). Diagnosis of autism disorder from rs-fMRI brain images through hierarchical YOLO and mechanism of attention (HYMA). Appl. Soft Comput..

[155] Farooq M.U., Ullah Z., Gwak J. (2023). Residual attention based uncertainty-guided mean teacher model for semi-supervised breast masses segmentation in 2D ultrasonography. Comput. Med. Imaging Graph..

[156] Liu H., Gao W., Yang L., Wu D., Zhao D., Chen K., Liu J., Ye Y., Xu R.X., Sun M. (2024). Semantic uncertainty Guided Cross-Transformer for enhanced macular edema segmentation in OCT images. Comput. Biol. Med..

[157] Swathi Kumari R., Geetha S., Rajakumar P.S. (2026). Radiographic Variability-Adaptive Structural Harmonization (RV-ASH): An Adaptive Deep Learning Framework for Robust Knee Osteoarthritis Detection and Clinical Validation. Int. J. Comput. Inf. Syst. Ind. Manag. Appl..

[158] Ahn S.H., Baek S., Park J., Kim J., Rhee H., Chung Y.E., Kim H.g, Lee Y.H. (2025). Uncertainty Quantification in Automated Detection of Vertebral Metastasis Using Ensemble Monte Carlo Dropout. J. Imaging Inform. Med..

[159] Abdar M., Fahami M.A., Rundo L., Radeva P., Frangi A.F., Acharya U.R., Khosravi A., Lam H.-K., Jung A., Nahavandi S. (2023). Hercules: Deep Hierarchical Attentive Multilevel Fusion Model with Uncertainty Quantification for Medical Image Classification. IEEE Trans. Ind. Inform..

[160] Saravana Ram R., Akilandeswari J., Vinoth Kumar M. (2023). HybDeepNet: A Hybrid Deep Learning Model for Detecting Cardiac Arrhythmia from ECG Signals. Inf. Technol. Control.

[161] Victor O.A., Chen Y., Ding X. (2024). Non-Invasive Heart Failure Evaluation Using Machine Learning Algorithms. Sensors.

[162] Fradi M., Khriji L., Machhout M. (2022). Real-time arrhythmia heart disease detection system using CNN architecture based various optimizers-networks. Multimed. Tools Appl..

[163] Sadeghi Nia P., Danandeh Hesar H. (2024). Abnormal Heart Sound Detection using Time-Frequency Analysis and Machine Learning Techniques. Biomed. Signal Process. Control.

[164] Chopannejad S., Roshanpoor A., Sadoughi F. (2024). Attention-assisted hybrid CNN-BILSTM-BiGRU model with SMOTE–Tomek method to detect cardiac arrhythmia based on 12-lead electrocardiogram signals. Digit. Health.

[165] Panwar A., Narendra M., Arya A., Raj R., Kumar A. (2025). Integrated portable ECG monitoring system with CNN classification for early arrhythmia detection. Front. Digit. Health.

[166] Padhy S.K., Mohapatra A., Patra S. (2025). A lightweight efficientNetB3 explainable model for enhancing prediction of cardiac arrhythmia using ECG signals. Netw. Model. Anal. Health Inform. Bioinform..

[167] Elsheikhy D.H., Hassan A.S., Yhiea N.M., Fareed A.M., Rashed E.A. (2025). Lightweight Deep Learning Architecture for Multi-Lead ECG Arrhythmia Detection. Sensors.

[168] Fradi M., Khriji L., Machhout M., Hossen A. (2021). Automatic heart disease class detection using convolutional neural network architecture-based various optimizers-networks. IET Smart Cities.

[169] Albarrak A.M., Alharbi R., Ibrahim I.A. (2025). Detection and Classification of Unhealthy Heartbeats Using Deep Learning Techniques. Sensors.

[170] Tripathi T., Kumar R. (2024). Speech-based detection of multi-class Alzheimer’s disease classification using machine learning. Int. J. Data Sci. Anal..

[171] Bravo-Ortiz M.A., Guevara-Navarro E., Holguín-García S.A., Rivera-Garcia M., Cardona-Morales O., Ruz G.A., Tabares-Soto R. (2024). SpectroCVT-Net: A convolutional vision transformer architecture and channel attention for classifying Alzheimer’s disease using spectrograms. Comput. Biol. Med..

[172] Singh S., Singh S., Sahu G., Jadon J., Dutta M.K. (2023). SZ-RAN: A Residual Attention Network for Early Detection of Schizophrenia using EEG Signals. 2023 9th International Conference on Signal Processing and Communication.

[173] Saleem A., Khan M.A., Yousaf H.M. (2023). Advancing Epilepsy Disease Classification through Machine Learning and Deep Learning Models Utilizing EEG Data. 2023 17th International Conference on Open Source Systems and Technologies.

[174] Tripathy S., Kumar B., Rath A., Panda G. (2025). CACBH-Net: A CNN-Attention BiLSTM Hybrid Deep Learning Model for Enhanced Detection of Epileptic Seizure. ICoICC 2025–3rd International Conference on Intelligent and Cloud Computing.

[175] Chen L. (2023). A Deep Learning Method for Classification of Interictal and Preictal EEG Signals in Epileptic Seizure Prediction. Proceedings–2023 9th Annual International Conference on Network and Information Systems for Computers.

[176] Hussain F., Hussain M., AlAhmadi S. (2026). An Ensemble of Lightweight Convolutional Neural Networks for EEG-Based Major Depressive Disorder Detection. Lect. Notes Comput. Sci..

[177] Gulenc N.G., Ozturk M. (2025). Deep Learning–Based Depression Detection via Wavelet Coherence Analysis of EEG Signals. 2025 Innovations in Intelligent Systems and Applications Conference.

[178] Ogaili S.T., Al-Salman W. (2025). EEG Data Analysis Based on TFI and Machine Learning Approaches for Epileptic Seizure Detection. Lect. Notes Netw. Syst..

[179] Dong X., Chen X., Chen Q., Liu X., Chen H., Aarts R.M., Yin B. (2026). A Multi-Scale Hybrid Efficient Deep Learning Model for COPD Detection Using Respiratory Sounds. IEEE J. Biomed. Health Inform..

[180] Wang D., Ding Y., Zhao Q., Yang P., Tan S., Li Y. (2022). ECAPA-TDNN Based Depression Detection from Clinical Speech. Proc. Annu. Conf. Int. Speech Commun. Assoc..

[181] Kazerani R. (2024). Improving Breast Cancer Diagnosis Accuracy by Particle Swarm Optimization Feature Selection. Int. J. Comput. Intell. Syst..

[182] Munshi R.M., Cascone L., Alturki N., Saidani O., Alshardan A., Umer M. (2024). A novel approach for breast cancer detection using optimized ensemble learning framework and XAI. Image Vis. Comput..

[183] Alshayeji M.H., Ellethy H., Abed S., Gupta R. (2022). Computer-aided detection of breast cancer on the Wisconsin dataset: An artificial neural networks approach. Biomed. Signal Process. Control.

[184] Dalal S., Onyema E.M., Kumar P., Maryann D.C., Roselyn A.O., Obichili M.I. (2023). A hybrid machine learning model for timely prediction of breast cancer. Int. J. Model..

[185] Kesiku C.Y., Garcia-Zapirain B. (2025). AI-Enhanced Lung Cancer Prediction: A Hybrid Model’s Precision Triumph. IEEE J. Biomed. Health Inform..

[186] Zapata-Paulini J., Cabanillas-Carbonell M. (2025). Performance analysis of 10 machine learning models in lung cancer prediction. Indones. J. Electr. Eng. Comput. Sci..

[187] Ali M.M., Ahmed K., Bui F.M., Paul B.K., Ibrahim S.M., Quinn J.M.W., Moni M.A. (2021). Machine learning-based statistical analysis for early stage detection of cervical cancer. Comput. Biol. Med..

[188] Islam M.A., Majumder M.Z.H., Miah M.S.l, Jannaty S. (2024). Precision healthcare: A deep dive into machine learning algorithms and feature selection strategies for accurate heart disease prediction. Comput. Biol. Med..

[189] Asif D., Bibi M., Arif M.S., Mukheimer A. (2023). Enhancing Heart Disease Prediction through Ensemble Learning Techniques with Hyperparameter Optimization. Algorithms.

[190] Ganie S.M., Pramanik P.K.D., Malik M.B., Nayyar A., Kwak K.S. (2023). An Improved Ensemble Learning Approach for Heart Disease Prediction Using Boosting Algorithms. Comput. Syst. Sci. Eng..

[191] Majumder A.B., Gupta S., Singh D., Acharya B., Gerogiannis V.C., Kanavos A., Pintelas P. (2023). Heart Disease Prediction Using Concatenated Hybrid Ensemble Classifiers. Algorithms.

[192] Sianga B.E., Mbago M.C., Msengwa A.S. (2025). Predicting the prevalence of cardiovascular diseases using machine learning algorithms. Intell.-Based Med..

[193] Kanza R.A., Al Rasyid M.U.H., Sukaridhoto S. (2024). Efficient Early Detection of Patient Diagnosis and Cardiovascular Disease using an IoT System with Machine Learning and Fuzzy Logic. Int. J. Comput. Digit. Syst..

[194] Cheng C.-H., Lee B.-J., Nfor O.N., Hsiao C.-H., Huang Y.-C., Liaw Y.-P. (2024). Using machine learning-based algorithms to construct cardiovascular risk prediction models for Taiwanese adults based on traditional and novel risk factors. BMC Med. Inform. Decis. Mak..

[195] Dissanayake K., Johar M.G.M. (2023). Two-level boosting classifiers ensemble based on feature selection for heart disease prediction. Indones. J. Electr. Eng. Comput. Sci..

[196] Abdullah M. (2024). Artificial intelligence-based framework for early detection of heart disease using enhanced multilayer perceptron. Front. Artif. Intell..

[197] Subathra R., Sumathy V. (2025). A smart CardioSenseNet framework with advanced data processing models for precise heart disease detection. Comput. Biol. Med..

[198] Tabassum S., Muhammad F., Khan M.A., Khan M.U., Awan D., Gohar N., Khan S., Al-Rasheed A. (2025). A Machine Learning-Based Framework for Heart Disease Diagnosis Using a Comprehensive Patient Cohort. Computers.

[199] Khanam J.J., Foo S.Y. (2021). A comparison of machine learning algorithms for diabetes prediction. ICT Express.

[200] García-Ordás M.T., Benavides C., Benítez-Andrades J.A., Alaiz-Moretón H., García-Rodríguez I. (2021). Diabetes detection using deep learning techniques with oversampling and feature augmentation. Comput. Methods Programs Biomed..

[201] Alzubaidi A.A., Halawani S.M., Jarrah M. (2024). Integrated Ensemble Model for Diabetes Mellitus Detection. Int. J. Adv. Comput. Sci. Appl..

[202] Abdalrada A.S., Neamah A.F., Murad H. (2024). Predicting Diabetes Disease Occurrence Using Logistic Regression: An Early Detection Approach. Iraqi J. Comput. Sci. Math..

[203] Sayed H.E.F., Abdelhafeez A., Hassan M.N.M.M. (2026). Predicting Type 2 Diabetes based on Machine Learning and Deep Learning Models. Statistics.

[204] Aleesa R.S., Al-bermani N.k., Ismael H.A. (2025). Type 2 Diabetes Data Classification Using an Attention-based Stacked Autoencoder and TabNet Hybrid Model. Int. J. Intell. Eng. Syst..

[205] Sivasankari S.S., Surendiran J., Yuvaraj N., Ramkumar M., Ravi C.N., Vidhya R.G. Classification of Diabetes using Multilayer Perceptron. Proceedings of the 2022 International Conference on Distributed Computing and Electrical Circuits and Electronics (ICDCECE).

[206] Kate C., Deepika G., Sravya M.N., Bokka R., Kumar S.K., Ganesan S. (2025). Enhanced Diabetes Diagnosis Using Ensemble Classifiers with Explainable AI and Oversampling for Imbalanced Data. 2025 5th International Conference on Intelligent Technologies.

[207] Nurjahan, Rony M.A.T., Satu M.S., Whaiduzzaman M. (2021). Mining Significant Features of Diabetes through Employing Various Classification Methods. 2021 International Conference on Information and Communication Technology for Sustainable Development.

[208] Talukder M.S.H., Nur A.H., Zaman S., Noor M.R., Khan M.A.U., Amir F. (2024). Optimizing Diabetes Prediction Accuracy: A Comprehensive Approach with Advanced Preprocessing and Diverse Machine Learning Classifiers. 2024 3rd International Conference on Advancement in Electrical and Electronic Engineering.

[209] Sunny S., Pinky S., Jalal S., Kayser M.i, Wadud M., Mansoor N. (2024). Soft Voting Ensemble- Based Approach for Diagnosing Diabetes Mellitus. 2024 International Conference on Advances in Computing.

[210] Ozdemir R., Ozturk C., Tasyurek M., Aslantas V. (2023). A New Deep Learning Model for Early Stage of Diabetes Disease Prediction. 2023 Innovations in Intelligent Systems and Applications Conference.

[211] Martinez-Eguiluz M., Arbelaitz O., Gurrutxaga I., Muguerza J., Perona I., Murueta-Goyena A., Acera M., Del Pino R., Tijero B., Gomez-Esteban J.C. (2023). Diagnostic classification of Parkinson’s disease based on non-motor manifestations and machine learning strategies. Neural Comput. Appl..

[212] Chakraborty M., Naoal N., Momen S., Mohammed N. (2024). ANALYZE-AD: A comparative analysis of novel AI approaches for early Alzheimer’s detection. Array.

[213] Ramu K., Patthi S., Prajapati Y.N., Ramesh J.V.N., Banerjee S., Rao K.B.V.B., Alzahrani S.I., Ayyasamy R. (2025). Hybrid CNN-SVM model for enhanced early detection of Chronic kidney disease. Biomed. Signal Process. Control.

[214] Saif D., Sarhan A.M., Elshennawy N.M. (2024). Deep-kidney: An effective deep learning framework for chronic kidney disease prediction. Health Inf. Sci. Syst..

[215] Arif M.S., Mukheimer A., Asif D. (2023). Enhancing the Early Detection of Chronic Kidney Disease: A Robust Machine Learning Model. Big Data Cogn. Comput..

[216] Kumar A., Sinha N., Bhardwaj A., Goel S. (2022). Clinical risk assessment of chronic kidney disease patients using genetic programming. Comput. Methods Biomech. Biomed. Eng..

[217] Alturki N., Altamimi A., Umer M., Saidani O., Alshardan A., Alsubai S., Omar M., Ashraf I. (2024). Improving Prediction of Chronic Kidney Disease Using KNN Imputed SMOTE Features and TrioNet Model. CMES Comput. Model. Eng. Sci..

[218] Fathima Begum M., Narayan S. (2023). A pattern mixture model with long short-term memory network for acute kidney injury prediction. J. King Saud Univ. Comput. Inf. Sci..

[219] Pradeepa P., Jeyakumar D.M.k. (2024). Chronic kidney disease prediction using improved deep belief network with local search nearest neighbour optimization. Comput. Methods Biomech. Biomed. Eng. Imaging Vis..

[220] Al-Rasheed A., Saqib S.M., Asghar M.Z., Mazhar T., Osman A.S.A., Shahid M., Iqbal M., Khan M.A. (2025). Classifying kidney disease using a dense layers deep learning model. SLAS Technol..

[221] Sutikno, Kusumaningrum R., Sugiharto A., Wibawa H.A. (2024). Detection of chronic kidney disease using binary whale optimization algorithm. IAES Int. J. Artif. Intell..

[222] Mondal P.K., Byeon H. (2024). Classification of Liver Disease Using Conventional Tree-Based Machine Learning Approaches with Feature Prioritization Using a Heuristic Algorithm. Int. J. Adv. Comput. Sci. Appl..

[223] Shukla G., Dhuriya G., Pillai S.K., Saini A. (2023). Chronic Kidney Disease Prediction Using Machine Learning Algorithms and the Important Attributes for the Detection. 2023 IEEE IAS Global Conference on Emerging Technologies.

[224] Carvalho F.R., Gavaia P.J. (2025). Enhancing osteoporosis risk prediction using machine learning: A holistic approach integrating biomarkers and clinical data. Comput. Biol. Med..

[225] Tao X., Chi O., Delaney P.J., Li L., Huang J. (2021). Detecting depression using an ensemble classifier based on Quality of Life scales. Brain Inform..

[226] Nishat M.M., Faisal F., Hasan T., Nasrullah S.M., Bristy A.H., Minhajul Islam Shawon M., Ashraful Hoque M. (2022). Detection of Autism Spectrum Disorder by Discriminant Analysis Algorithm. Lect. Notes Data Eng. Commun. Technol..

[227] Singh U., Shukla S., Gore M.M. (2022). An Improved Feature Selection Algorithm for Autism Detection. 9th IEEE Uttar Pradesh Section International Conference on Electrical.

[228] Boughattas N., Jabnoun H. (2022). Autism Spectrum Disorder (ASD) Detection Using Machine Learning Algorithms. Participative Urban Health and Healthy Aging in the Age of AI.

[229] Shambour Q.Y., Shambour M.K., Alraba’nah Y., Abualhaj M.M. (2025). Leveraging Feature Selection and Explainable AI for Effective Autism Screening. Proceeding–12th International Conference on Information Technology: Innovation Technologies.

[230] Yesilkaya B., Perc M., Isler Y. (2022). Manifold learning methods for the diagnosis of ovarian cancer. J. Comput. Sci..

[231] Rajesh A.S., Bala Sai Sreeja Kumari G., Rahul Chiranjeevi V. (2025). Integrating Autoencoders for Detection of Spinal Muscular Atrophy Based on Transcriptomic Data. ICRTEECT 2025–2nd International Conference on Recent Trends in Electrical.

[232] Priya S.B., Mahendira Kumar S., Madeswaran T. (2024). Prediction of Genetic Diseases using Genomic Data and XGBoost Algorithm: A High-Accuracy Approach. 2024 IEEE International Conference on Intelligent Signal Processing and Effective Communication Technologies.

[233] Elmahy A., Aly S., Elkhwsky F. (2021). Cancer Stage Prediction from Gene Expression Data Using Weighted Graph Convolution Network. 2021 2nd International Conference on Innovative and Creative Information Technology.

[234] Dalal S., Lilhore U.K., Faujdar N., Simaiya S., Agrawal A., Rani U., Mohan A. (2025). Enhancing thyroid disease prediction with improved XGBoost model and bias management techniques. Multimed. Tools Appl..

[235] Poh M.-Z., Battisti A.J., Cheng L.-F., Lin J., Patwardhan A., Venkataraman G.S., Athill C.A., Patel N.S., Patel C.P., Machado C.E. (2023). Validation of a Deep Learning Algorithm for Continuous, Real-Time Detection of Atrial Fibrillation Using a Wrist-Worn Device in an Ambulatory Environment. J. Am. Heart Assoc..

[236] Chiu I.-M., Wu P.-J., Zhang H., Hughes J.W., Rogers A.J., Jalilian L., Perez M., Lin C.-H.R., Lee C.-T., Zou J. (2024). Serum Potassium Monitoring Using AI-Enabled Smartwatch Electrocardiograms. JACC Clin. Electrophysiol..

[237] Karolcik S., Manginas V., Chanh H.Q., Daniels J., Giang N.T., Huyen V.N.T., Hoang M.T.V., Phan Nguyen Quoc K., Hernandez B., Ming D.K. (2024). Towards a machine-learning assisted non-invasive classification of dengue severity using wearable PPG data: A prospective clinical study. eBioMedicine.

[238] Shah S., Gonzalez Gutierrez E., Hopp J.L., Wheless J., Gil-Nagel A., Krauss G.L., Crone N.E. (2024). Prospective multicenter study of continuous tonic-clonic seizure monitoring on Apple Watch in epilepsy monitoring units and ambulatory environments. Epilepsy Behav..

[239] Hadid A., McDonald E.G., Ding Q., Phillipp C., Trottier A., Dixon P.C., Jlassi O., Cheng M.P., Papenburg J., Libman M. (2025). Development of machine learning prediction models for systemic inflammatory response following controlled exposure to a live attenuated influenza vaccine in healthy adults using multimodal wearable biosensors in Canada: A single-centre, prospective controlled trial. Lancet Digit. Health.

[240] Zheng X., Zeng Z.i, van Schooten K.S., Yang Y. (2025). Machine Learning Approach for Frailty Detection in Long-Term Care Using Accelerometer-Measured Gait and Daily Physical Activity: Model Development and Validation Study. JMIR Aging.

[241] Nasseri M., Stirling R.E., Viana P.F., Cui J., Nurse E., Karoly P.J., Kremen V., Dumpelmann M., Worrell G.A., Freestone D.R. (2025). Forecasting epileptic seizures with wearable devices: A hybrid short- and long-horizon pseudo-prospective approach. Epilepsia.

[242] Wu C.-T., Li G.-H., Huang C.-T., Cheng Y.-C., Chen C.-H., Chien J.-Y., Kuo P.-H., Kuo L.-C., Lai F. (2021). Acute exacerbation of a chronic obstructive pulmonary disease prediction system using wearable device data, machine learning, and deep learning: Development and cohort study. JMIR MHealth UHealth.

[243] Mohammadi Z., Fazel P., Mohammadi S. (2026). Energy-efficient real-time 4-stage sleep classification at 10-s resolution. Med. Biol. Eng. Comput..

[244] Alkhateeb M., AlSaad R., Brahim Belhaouari S., Aziz S., Ahmed A., Ali H., Al-Mohanadi D., Mohamud K., Al-Naimi N., Alsaud A. (2026). Temporal and Behaviour-Aware Multimodal Modelling for Hour-Ahead Hypoglycaemia Prediction During Ramadan Fasting in Type 1 Diabetes. Sensors.

[245] Dong C., van Dijk J.P., Aarts R.M., Wang Y., Long X. (2026). Detection of nocturnal epileptic seizures using a wearable armband: A deep learning approach combining accelerometry and photoplethysmography signals. Comput. Methods Programs Biomed..

[246] Zuo S., Wang J., Wang X., Feng H., Zhao Y., Li F., Wei S., Zhou L., Zhao Z., Chen S. (2026). A Dual-Modal Wearable PPG Smartwatch with AI-Enhanced Correction for High-Accuracy and Continuous AF Burden Assessment. Adv. Sci..

[247] Bossi F., Laurino M., Carbonaro N., Waschki B., Watz H., Abdo M., Tognetti A., Greco A. (2026). FEV1 Prediction From Sensor Data of a Physical Activity Armband in COPD Patients: Results From the COSYCONET Cohort. IEEE Access.

[248] Alhaskir M., Kutafina E., Linke F., Fischer F.P., Schriewer E., Lauxmann S., Klett K., Hofmeister J., Lutz F., Burow L. (2026). Reliable detection of focal onset impaired awareness seizures in patients with epilepsy using wearable ECG: Development and validation study. Comput. Methods Programs Biomed..

[249] Al-Absi H.R.H., Islam M.T., Refaee M.A., Chowdhury M.E.H., Alam T. (2022). Cardiovascular Disease Diagnosis from DXA Scan and Retinal Images Using Deep Learning. Sensors.

[250] Zhu J., Liu H., Liu X., Chen C., Shu M. (2025). Cardiovascular disease detection based on deep learning and multi-modal data fusion. Biomed. Signal Process. Control.

[251] Chawla V., Rana B. (2025). Early fusion for Autism classification with biomarker detection using rs-fMRI and phenotypic data. Biomed. Signal Process. Control.

[252] Arumugam C., Ramesh P.S., Jagannathan J., Senthilvadivu S., Sudha I., Aparaj S. (2026). Transformers-Based Multi-Modal Deep Learning Framework for Early Cancer Detection Using ViT and Clinical Data Fusion. 2026 2nd International Conference on Computing for Sustainability and Intelligent Future.

[253] Ahmed M.M., Hossain M.M., Rakib M.R.H., Hashan R., Nirob M.T.H., Islam M.K. (2026). A hybrid swin transformer–BiLSTM framework and ensemble learning for multimodal brain stroke detection and risk prediction. Comput. Biol. Med..

[254] Narasimha Murthy G.K., Supriya M.C. (2026). Convolutional Radiographic Encoding Combined with Dense Clinical Feature Embedding for Osteoporosis Diagnosis. Proceedings of the 2nd International Conference on Multi-Agent Systems for Collaborative Intelligence.

[255] Amudha T.K., Sunitha R., Syam M.E. (2024). A Study on DL for Pulmonary Embolism Prediction Harnessing Multimodal Data. Lect. Notes Netw. Syst..

[256] Khan A.H., Li J., Sajid M. (2026). 3D-FuseNet: Attention-guided multi-modal fusion for early-stage pancreatic cancer detection. J. Big Data.

[257] Siebert M., Grasshoff J., Rostalski P. (2023). Uncertainty Analysis of Deep Kernel Learning Methods on Diabetic Retinopathy Grading. IEEE Access.

[258] Jahin M.A., Masud M.A., Mridha M.F., Aung Z., Dey N. (2025). KACQ-DCNN: Uncertainty-Aware Interpretable Kolmogorov–Arnold Classical–Quantum Dual-Channel Neural Network for Heart Disease Detection. Comput. Biol. Med..

[259] Reis S.S., Pinto-Coelho L., Sousa M.C., Neto M., Silva M., Sequeira M. (2025). Evaluating Skin Tone Fairness in Convolutional Neural Networks for the Classification of Diabetic Foot Ulcers. Appl. Sci..

[260] Li Y., Cui Z., Mei L., Xie Y., Marini L., Pelekos G., Gu W., Yu X., Wu X., Wei X. (2025). A novel AI-powered radiographic analysis surpasses specialists in stage II–IV periodontitis detection: A multicenter diagnostic study. npj Digit. Med..

[261] Vasey B., Nagendran M., Campbell B., Clifton D.A., Collins G.S., Denaxas S., Denniston A.K., Faes L., Geerts B., Ibrahim M. (2022). Reporting guideline for the early-stage clinical evaluation of decision support systems driven by artificial intelligence: DECIDE-AI. Nat. Med..

[262] Ahmad A., Hashmi M.U., Azam M., Sarwar R., Ali M. (2026). A Leakage-Free, Patient-Level Evaluation Protocol for Multi-Class Lung CT Diagnosis. Eng. Rep..

[263] Shiferaw K.B., Roloff M., Balaur I., Welter D., Waltemath D., Zeleke A.A. (2025). Guidelines and standard frameworks for artificial intelligence in medicine: A systematic review. JAMIA Open.

[264] Talebi M., Bozorgchami N., Mishra G., Mishra G., Almasi Ghale R. (2026). Clinical validation of artificial intelligence for gastrointestinal diseases. Inform. Med. Unlocked.

